# Identification and Experimental Validation of Marker Genes between Diabetes and Alzheimer's Disease

**DOI:** 10.1155/2022/8122532

**Published:** 2022-08-12

**Authors:** Cheng Huang, Xueyi Wen, Hesong Xie, Di Hu, Keshen Li

**Affiliations:** ^1^Department of Neurology and Stroke Center, The First Affiliated Hospital of Jinan University, Guangzhou, China; ^2^Clinical Neuroscience Institute of Jinan University, Guangzhou, China

## Abstract

Currently, Alzheimer's disease (AD) and type 2 diabetes mellitus (T2DM) are widely prevalent in the elderly population, and accumulating evidence implies a strong link between them. For example, patients with T2DM have a higher risk of developing neurocognitive disorders, including AD, but the exact mechanisms are still unclear. This time, by combining bioinformatics analysis and in vivo experimental validation, we attempted to find a common biological link between AD and T2DM. We firstly downloaded the gene expression profiling (AD: GSE122063; T2DM: GSE161355) derived from the temporal cortex. To find the associations, differentially expressed genes (DEGs) of the two datasets were filtered and intersected. Based on them, enrichment analysis was carried out, and the least absolute shrinkage and selection operator (LASSO) logistic regression and support vector machine-recursive feature elimination (SVM-RFE) algorithms were used to identify the specific genes. After verifying in the external dataset and in the samples from the AD and type 2 diabetes animals, the shared targets of the two diseases were finally determined. Based on them, the ceRNA networks were constructed. Besides, the logistic regression and single-sample gene set enrichment analysis (ssGSEA) were performed. As a result, 62 DEGs were totally identified between AD and T2DM, and the enrichment analysis indicated that they were much related to the function of synaptic vesicle and MAPK signaling pathway. Based on the evidence from external dataset and RT-qPCR, CARTPT, EPHA5, and SERPINA3 were identified as the marker genes in both diseases, and their clinical significance and biological functions were further analyzed. In conclusion, discovering and exploring the marker genes that are dysregulated in both 2 diseases could help us better comprehend the intrinsic relationship between T2DM and AD, which may inspire us to develop new strategies for facing the dilemmas of clinical or basic research in cognitive dysfunction.

## 1. Introduction

Alzheimer's disease (AD), the leading cause of dementia, is emerging as a major global health challenge. Clinically, patients show a cognitive decline, accompanied by significant psychobehavioral abnormalities and impaired social life [1]. However, the molecular mechanism that can effectively explain this abnormal alteration is not yet clear. Usually, several nonspecific factors, such as age, vascular disease, infection, and environmental changes, are thought to play a role [2]. Currently available drugs developed to target these factors only slow the progression of the disease, not cure or prevent it. The realistic quandary forces us to expand theoretical hypotheses. Now, dysglycaemia involving the central nervous system (CNS) appears to be the next frontier in AD research [3].

Approximately 6% of the global population is affected by type 2 diabetes mellitus, and the prevalence of this chronic endocrine disease is rising [4, 5]. In-depth research on glucose metabolism brings new insights into our understanding of AD-related mechanisms. At present, a close association between AD and T2DM has been found. Epidemiological evidence shows much greater impairments in executive function, processing speed, and verbal memory plague adults with T2DM [6], and they have a higher incidence of cognitive dysfunction compared with the general population [7, 8]. Insulin is a major polypeptide hormone that plays crucial roles in the brain, including the release or reuptake of neurotransmitters, the improvement of learning and memory abilities, and the activation of signal transduction cascades leading to long-term memory consolidation [9]. Besides, research shows the involvement of insulin in the activation of glycogen synthase kinase 3*β*, which leads to the phosphorylation of tau and the formation of neurofibrillary tangles [10]. It can be seen that the disorder of blood glucose metabolism in the brain may be closely involved in the pathological changes of Alzheimer's disease. So some people refer to Alzheimer's disease as “brain diabetes” [11]. On this basis, studies on specific brain regions are still preliminary.

Studies have demonstrated that impairment of executive ability and memory is associated with the reduced gray matter density and glucose metabolism in the temporal cortex (middle gyrus, parahippocampus, and uncinate lobe) [12]. Diabetics are at risk for brain structural changes [13], and the medial temporal structures are vulnerable to being involved, causing abnormal atrophy of the hippocampus and amygdala [14]. This has some similarities with Alzheimer's disease and maybe one of the neural mechanisms of type 2 diabetes patients' easy transformation to dementia. These suggest that temporal lobe abnormalities play an important role in type 2 diabetes-related cognitive impairment [15].

To figure out the association between Alzheimer's disease and type 2 diabetes as precisely as possible and determine the mechanisms and targets that potentially regulate their interrelationships in the temporal lobe, an exploratory method that combines high-throughput gene expression detection technology with bioinformatics was mainly employed to discover the molecular markers and quest their subtle physiological functions in this research. Based on the Gene Expression Omnibus (GEO) database (https://www.ncbi.nlm.nih.gov/geo/), we firstly identified the codysregulated genes in the temporal cortex, respectively, obtained from the patients of type 2 diabetes and AD to try to find a genetic bridge. The protein-protein interaction (PPI) and enrichment analysis were then performed. Besides, the machine learning algorithms were introduced to further screen the potential markers. With the validation in the external dataset and animal samples, targets were finally confirmed. Their disease-related risks and regulatory factors, such as miRNAs and interacting drugs, were predicted. These findings may provide a deeper insight into the molecular interactions between type 2 diabetes and Alzheimer's disease, assisting us in discovering new regimens for the disease transformation.

## 2. Materials and Methods

**Figure 1:**
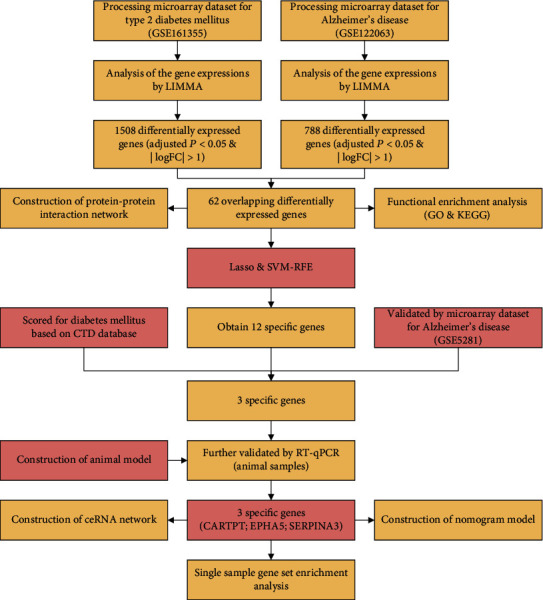
The whole analysis flow for this study.

The whole analysis flow of this study is shown in Figure 1.

### 2.1. Microarray Data

Gene expression profiling in this work was downloaded from the NCBI-GEO database [16]. Specifically, the GSE161355 [17] dataset for the human temporal cortex (T2DM: 6 cases; normal controls: 5 cases) was executed on the GPL570 platform; the GSE122063 [18] (AD: 28 cases; normal controls: 22 cases) and GSE5281 (AD: 16 cases; normal controls: 12 cases) datasets for the human temporal cortex were, respectively, based on the GPL16699 and the GPL570 platforms.

### 2.2. Data Processing

R software (version 4.0.2) and Bioconductor packages (http://www.bioconductor.org/) [19] were subsequently applied in the data processing.

For the .CEL format files (GSE161355), the “affy” [20] (version 1.66.0), and “affyPLM” [21] (version 1.64.0) packages are used to process the raw data by the RMA (robust multiarray average) function firstly [22]. Then, the probe identification numbers were converted into the official gene symbols according to the GPL570 platform. If multiple probes correspond to one gene, the average value was selected. After processing the missing value of the gene expression profile file by the KNN (k-nearest neighbor) algorithm [23], the “LIMMA” package [24] (version 3.44.3) built-in R was used to identify the differentially expressed genes (DEGs; adjusted *P* < 0.05 and |logFC| > 1 were set as the cutoff criteria).

For the .txt format files (GSE122063 and GSE5281), the probe identification numbers were converted into the official gene symbols according to the GPL16699 and GPL570 platforms. The average expression was taken when multiple probes corresponded to the one. After log2 transformation and normalization, the “LIMMA” package (version 3.44.3) was used to identify the DEGs (adjusted *P* < 0.05 and |*logFC*| > 1 were set as the cutoff criteria). The GSE5281 dataset served as the validation set in this research.

### 2.3. Gene Ontology and Pathway Enrichment Analysis

Gene Ontology (GO) and Kyoto Encyclopedia of Genes and Genomes (KEGG) pathway enrichment analyses [25] were executed by using clusterProfiler package [26] (version 3.16.0) in R software (version 4.0.2) for function annotating and pathway predicting. When the results met the cutoff criterion (*P* < 0.05), it was considered statistically significant.

### 2.4. Construction of Protein-Protein Interaction (PPI) Network and Module Analysis

STRING (Search Tool for the Retrieval of Interacting Genes/Proteins; https://www.string-db.org/) [27] integrating multiple databases that provide information on candidate genes was employed for predicting the potential PPI network and detecting the possible associations (confidence score 0.4). Furthermore, the MCODE (version 1.6.1) and cytoHubba (version 0.1) plugin [28] built in the Cytoscape software (http://cytoscape.org/; version 3.7.2) were, respectively, used to identify the significant module and hub genes in the constructed network.

### 2.5. Screening and Validation of the Specific Genes in the Disease

The least absolute shrinkage and selection operator (LASSO) logistic regression [29] with the “glmnet” package (version 4.1-1) and the support vector machine-recursive feature elimination (SVM-RFE) [30] with the “e1071” package (version 1.7-6) were applied to screen the specific genes. The obtained results of the two algorithms were intersected and displayed in a Venn diagram, and all of them were further screened through the combination of Comparative Toxicogenomics Database (CTD; http://ctdbase.org/) [31] and the GSE5281 dataset. Besides, we used the pROC package (version 1.18.0) [32] of R to analyze the receiver operating characteristic (ROC) curve to evaluate their performance.

### 2.6. Experimental Animals

Adult C57BL/6 mice (male; 4-week-old; *n* = 10) were purchased for type 2 diabetes model construction, and they were randomly divided into the control (*n* = 5) and the diabetic group (*n* = 5). The newly purchased animals were fed with regular chow diet for one week. In the following 4 weeks, the control mice were continued to be regular fed, while the model mice were given high-fat diet [33]. Eight-month-old APP/PS1 mice (male; *n* = 5) were used as AD model in vivo, and age-matched C57BL/6 mice were the controls (male; *n* = 5). All animals were housed in standard polypropylene cages. During the period, they were allowed to free diet under a stable condition (lights on: 08 : 00 am; lights off: 20 : 00 pm; optimum temperature: 23 ± 2°C; suitable humidity: 55 ± 5%). All the animal experiments were approved by the Institutional Animal Care and Use Committee of Jinan University.

### 2.7. Type 2 Diabetes Model

Before the start of the experiment, another week of environmental adaptation was carried out. For the diabetic group, 45 mg/kg streptozocin (STZ; Solarbio Beijing) was intraperitoneally injected for one week, while the same volume of saline was injected into the controls. During the week of drug injection, we trained the mice on the Y maze for the first six days and performed the final behavioral test on the seventh day. The blood glucose in caudal venous was detected every two days. When random blood glucose > 16.7 mmol/L [34], they were considered diabetic.

### 2.8. Behavioral Test

The Y maze was applied to detect the memory ability of mice. The maze consists of three arms divided into 1 start arm and 2 nonstart arms. Each arm was 30 cm long, 15 cm high, and 10 cm wide with an angle of 120 degrees. Markers were set around the maze. The first six days are the training period. Each mouse was placed at the end of one start arm and allowed to freely move through the maze for adaptation over the course of 5 minutes. One (target arm) was randomly selected in the two nonstart arms, with food placed on the end and well marked, and the another (nontarget arm) was left untreated. After the adaptation, put the animal back into the end of the start arm, timing was initiated, and the latency and times for the animal to correctly enter the food arm were recorded. Each animal repeated 6 times daily. On the seventh day, the mark of the target arm was changed, and no food was put in. After the animal was put into the start arm, the duration and times of the animal entering the target arm were recorded. The test period of each mice was 5 minutes. The maximum number of arm alternations was defined as the number of occurrences in all arms minus 2, and the percentage of arm alternations was (number of occurrences in the target arm/maximum number of alternations) × 100 [35].

### 2.9. Sample Collection

After completing all tests, the experimental mice were decapitated. The mice were anesthetized with 15% pentobarbital sodium solution (intraperitoneal injection; 0.4 ml/100 g). Then, cardiac perfusion was performed by irrigation with 0.9% sodium chloride solution [36]. The temporal cortex was collected and stored at −80°C until molecule experiments.

### 2.10. Reverse Transcription Quantitative Real-Time Polymerase Chain Reaction (RT-qPCR)

RNAs were extracted from the temporal cortex of mice using TRIzol reagent (Invitrogen, CA, USA), and the concentration and purity were detected by Nanodrop. According to the manufacturer's instructions, we reverse-transcribed the RNAs into cDNAs with the PrimerScript RT Reagent Kit (Takara). With the SYBR Premix Ex Taq (Takara), RT-qPCR proceeded in the Bio-Rad CFX96 TouchTM system. The primer of different genes needed in our research is shown in the Supplementary Table 1. Target genes were normalized to GAPDH using the comparative CT method.

### 2.11. ceRNA Network Construction

The miRNAs interacting with the DEGs were predicted by the StarBase (http://starbase.sysu.edu.cn) database [37] or miRSystem (http://mirsystem.cgm.ntu.edu.tw/) database [38]. StarBase integrates seven well known miRNA target gene prediction programs: PITA, RNA22, miRmap, microT, miRanda, PicTar, and TargetScan, while miRSystem integrates DIANA, miRanda, miRBridge, PicTar, PITA, RNA22, and TargetScan. After comprehensive evaluation, the miRNAs hitting the most programs will be included in our research. The interaction between miRNA and lncRNAs/circRNAs was also predicted by using the StarBase. During the operation, we refer to the ClipExpNum to remove the weak interactions with miRNAs for net simplifying.

### 2.12. Nomogram Model

A nomogram model (“rms” package; version 6.2-0) [39] was built to predict the risk of AD. Using the calibration curve, the predictive ability of nomogram model was evaluated. In addition, decision curve analysis and clinical impact curve were used to assess the clinical value of the model.

### 2.13. The Gene-Drug Interaction Analysis

The Drug Gene Interaction Database (DGIdb) [40] (https://www.dgidb.org) provides information about the association of genes with their known or potential drugs. We searched the specific genes in it to explore their possible drugs and their directions.

### 2.14. Assessment of Hallmark Gene Sets and Immune Cell Infiltration

The relative levels of the 50 hallmark gene sets and the 28 immune cells in the GSE122063 dataset (AD) were quantified using ssGSEA algorithm [41]. Plots were generated to present the differential expression levels between the controls and AD. In addition, Spearman's correlations for the 50 hallmark gene sets and the 28 immune cells with the specific genes were calculated, which were visualized by using the “ggplot2” package [42] (version 3.3.2).

### 2.15. Statistical Analysis

Statistical analyses were executed using SPSS 23.0 (Chicago, USA). The results for the behavior test and molecular experiments are presented as mean ± SEM. For data examination, the parametric Student's *t*-test was employed. All tests were two-tailed. When *P* < 0.05, it was considered statistically significant.

## 3. Results

### 3.1. DEG Identification

**Figure 2:**
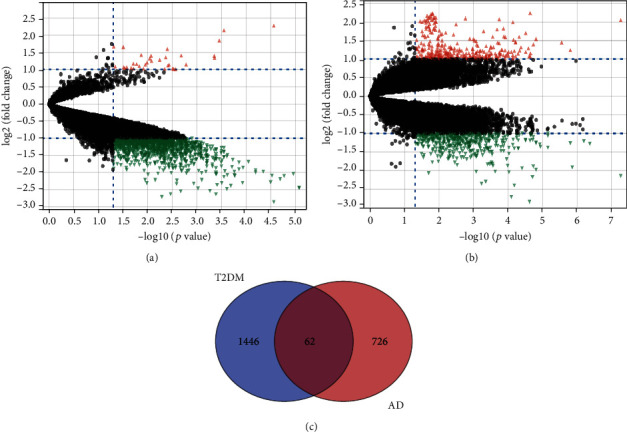
Volcano plot of differently expressed genes. (a) GSE161355 (T2DM), (b) GSE122063 (AD), and (c) the intersection of the two sets of DEGs: 62 genes.

The analysis of differentially expressed genes (GSE161355 or GSE122063) was executed by the “LIMMA” package (version 3.44.3) with the criteria of the |log2 FC| > 1 and adjusted *P* value < 0.05. In general, a total of 1508 DEGs (Supplementary file 1) were screened in human diabetes-associated temporal cortex, including 1473 downregulated genes and 35 upregulated genes, which were intuitively presented in a volcano map (Figure 2(a)). On the other side, 788 DEGs (Supplementary file 2) were identified in AD temporal cortex when compared to controls, including 475 downregulated genes and 313 upregulated genes, which were also exhibited in a volcano map (Figure 2(b)). Among the two sets of DEGs, there were 62 overlapping items (Figure 2(c)). Here, we used the overlapping for subsequent studies to explore the mechanisms linking diabetes to AD. Supplementary Figure 1 visualizes the expression level of these 62 genes in GSE122063 (AD) in the form of a heatmap.

### 3.2. Enrichment Analysis for the 62 Overlapping DEGs

**Figure 3:**
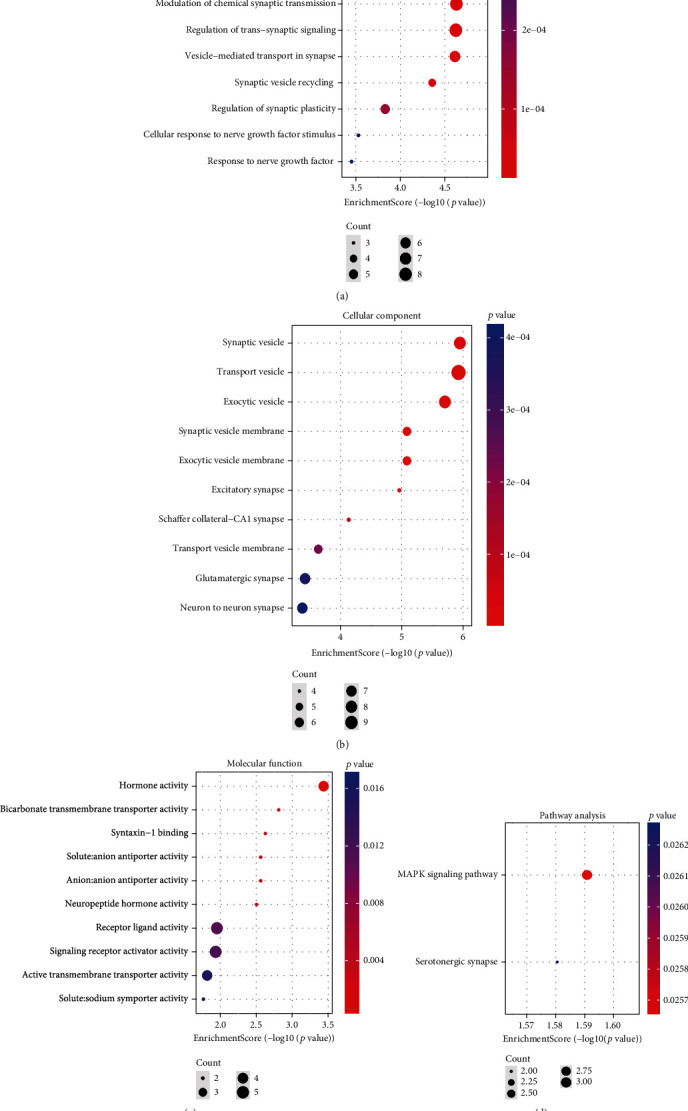
Bubble diagram displays the significant enrichment terms for the 62 DEGs. (a) BP terms, (b) CC terms, (c) MF terms, and (d) KEGG terms.

The terms of GO mainly consist of biological process (BP), cellular component (CC), and molecular function (MF). As shown in Figure 3(a), synaptic vesicle cycle (GO:0099504), synaptic vesicle endocytosis (GO:0048488), and presynaptic endocytosis (GO:0140238) were the most remarkable annotations in BP. For the CC (Figure 3(b)), most of the overlapping genes were enriched in synaptic vesicle (GO:0008021), transport vesicle (GO:0030133), and exocytic vesicle (GO:0070382). Among the significant MF enrichments (Figure 3(c)), hormone activity (GO:0005179), bicarbonate transmembrane transporter activity (GO:0015106), and syntaxin-1 binding (GO:0017075) were dominant. On the other hand, the MAPK signaling pathway (hsa04010) is highlighted in the KEGG pathway enrichments (Figure 3(d)).

### 3.3. PPI Network

**Figure 4:**
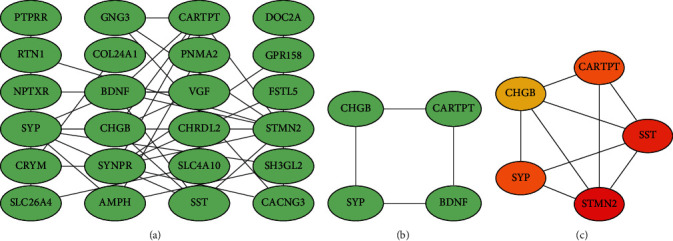
(a) PPI network constructed by the 62 DEGs (the disconnected nodes were hidden), (b) the most significant module in the network (score: 2.7), and (c) top 5 genes computed by the MCC algorithm (the darker the color, the higher the score).

All the 62 overlapping DEGs were then imported into the STRING for the PPI network construction, which were finally visualized by the Cytoscape (http://cytoscape.org/;version 3.7.2). This resulting network contained 24 nodes and 39 edges (Figure 4(a)) with a most significant module (Figure 4(b); score: 2.7) obtained by using the MCODE plugin (version 1.6.1) of the Cytoscape. Relying on the same software, we further captured the top 5 hub genes in the network through the MCC algorithm with the cytoHubba plugin (version 0.1) (Figure 4(c)).

### 3.4. Identification of the Specific Genes in Disease

**Figure 5:**
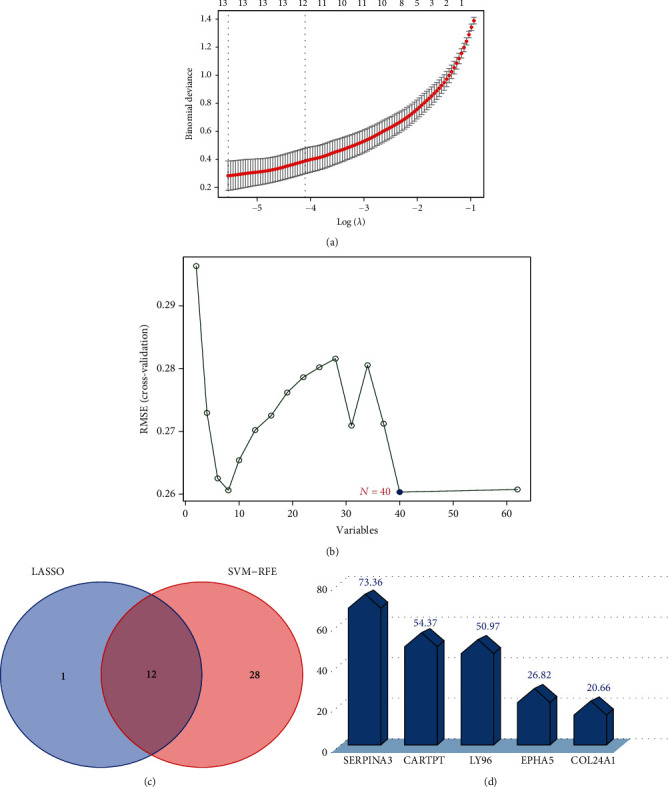
Machine learning algorithms for finding characteristic genes. (a) The LASSO logistic regression algorithm (13 genes). (b) The SVM-RFE algorithm (40 genes). (c) The intersection of the two algorithms (12 genes). (d) The inference score of T2DM based on the CTD database (of these overlapping 12 genes, the top 5 ranked were visualized).

We believe that these 62 overlapping genes are differentially altered in AD patients and susceptible to glycemic disturbances. Therefore, we extracted their expression values in the AD dataset for further study. Base on the gene expression matrix from the GSE122063 dataset, we identified 13 specific genes (IGLL5, COL24A1, C20orf195, LOC283737, SERPINA3, LPP-AS2, ZCCHC12, OSR1, CHRDL2, LY96, LOC100507165, EPHA5, and CARTPT) from the 62 overlapping DEGs with the LASSO logistic regression algorithm (Figure 5(a)). Furthermore, 40 specific genes (CARTPT, LOC283737, SERPINA3, RNF165, LOC100507165, COL24A1, TSPAN7, CHRDL2, C5orf55, DGKI, VGF, IGLL5, LY96, LOC100129973, KDM4D, SLC26A4, OSR1, C20orf195, SLC5A11, NPTXR, WDR54, MYOT, SST, LPP-AS2, ABCC12, BDNF, LY86-AS1, SYNPR, FSTL5, AMPH, ZWILCH, NRSN1, CHGB, CACNG3, PTPRR, COPG2IT1, CASQ1, NLGN4Y, C2orf80 and EPHA5) were also filtered using the SVM-RFE algorithm (Figure 5(b)). Subsequently, 12 genes (SERPINA3, CARTPT, LY96, EPHA5, COL24A1, OSR1, CHRDL2, IGLL5, LPP-AS2, C20orf195, LOC283737, and LOC100507165) were determined by the combination of the two algorithms (Figure 5(c)). We ranked these 12 genes according to their reference score involving diabetes in the CTD and chose the top five ranked genes (Figure 5(d)) for expression validation.

### 3.5. Verification of the Specific Genes in Datasets

**Figure 6:**
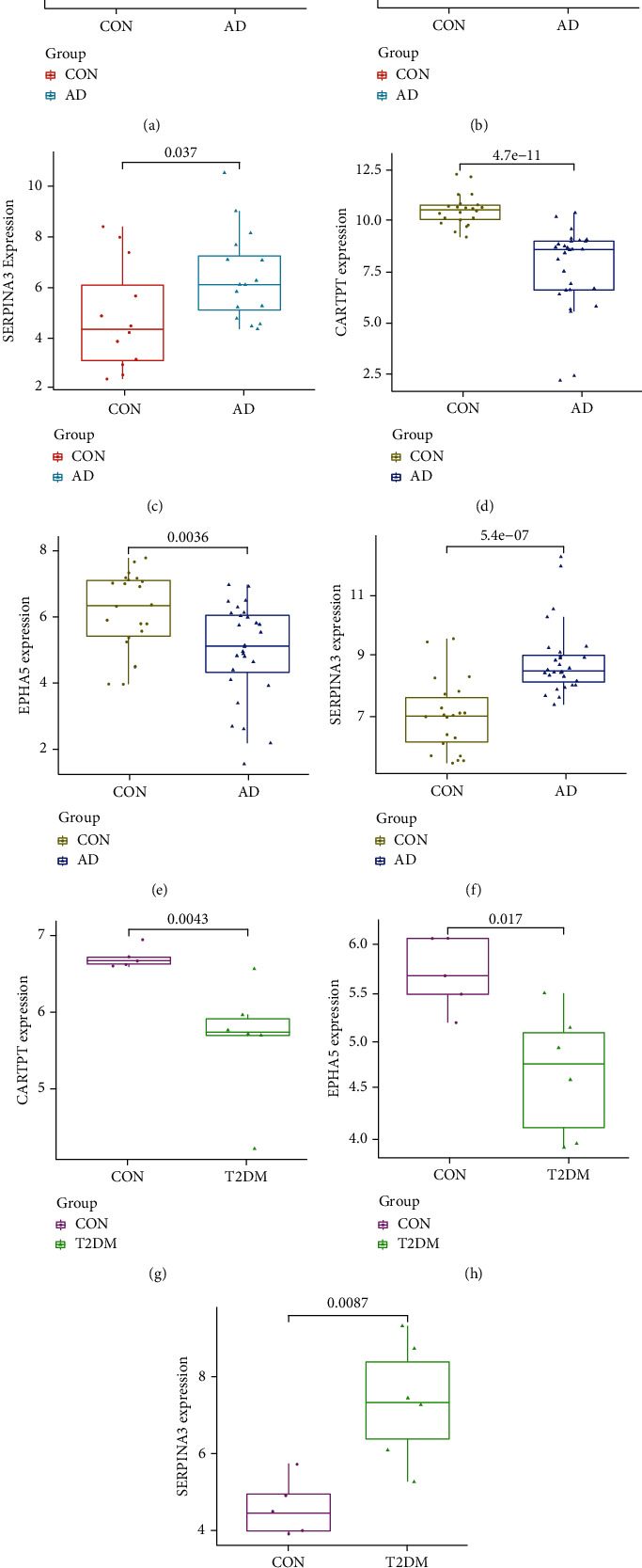
The expression value of CARTPT, EPHA5, and SERPINA3. (a) Validated in the GSE5281 (AD, *P* < 0.05), (b) expression value calculated based on the GSE122063 (AD, *P* < 0.05), and (c) expression value calculated based on the GSE161355 (T2DM, *P* < 0.05).

We validated the expression value of the five specific genes in the GSE5281 dataset (AD; the validation set; *P* < 0.05 was considered significant), and the results presented that CARTPT, EPHA5, and SERPINA3 met the criteria (Figures 6(a)–6(c)). Because the *P* value of COL24A1 (Supplementary Figure 2) and LY96 (Supplementary Figure 3) was 0.37 and 0.7, respectively, they were not available for the subsequent analysis. In addition, the expression value of CARTPT, EPHA5, and SERPINA3 in the GSE122063 (AD; the training set; Figures 6(d)–6(f)) and GSE161355 (T2DM; Figures 6(g)–6(i)) datasets was also calculated. The trend of the dysregulated expression for the three genes was consistent in the three different datasets.

**Figure 7:**
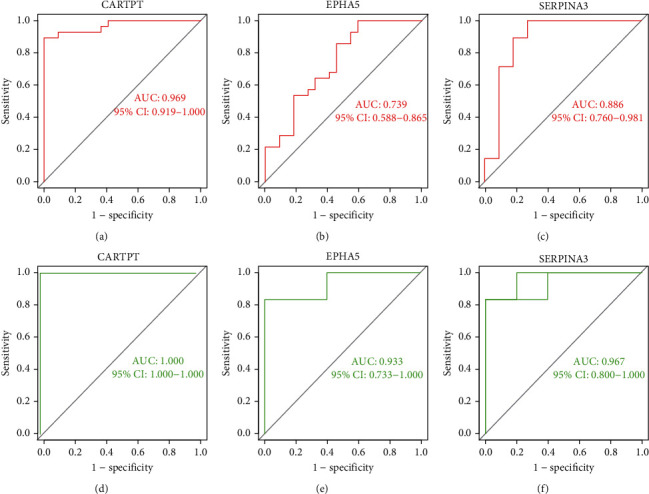
Diagnostic performance of CARTPT, EPHA5, and SERPINA3. (a–c) The ROC curves based on the GSE122063 (AD) and (d–f) the ROC curves based on GSE161355 (T2DM).

Consequently, we drew receiver operating characteristic (ROC) curve to further test their efficacy in the GSE122063 dataset (AD; the training set). For CARTPT, the area under the curve (AUC) was 0.969 and 95% CI: 0.919−1.000 (Figure 7(a)). For EPHA5, the area under the curve (AUC) was 0.739 and 95% CI: 0.588−0.865 (Figure 7(b)). For SERPINA3, the area under the curve (AUC) was 0.886 and 95% CI: 0.760−0.981 (Figure 7(c)). We also evaluated them in the GSE161355 dataset (T2DM). For CARTPT, the area under the curve (AUC) was 1.000 and 95% CI: 1.000−1.000 (Figure 7(d)); for EPHA5, the area under the curve (AUC) was 0.933 and 95% CI: 0.733−1.000 (Figure 7(e)); for SERPINA3, the area under the curve (AUC) was 0.967 and 95% CI: 0.800−1.000 (Figure 7(f)). All results indicated that CARTPT, EPHA5, and SERPINA3 had high diagnostic values in both AD and T2DM.

### 3.6. Animal Model Evaluation

**Figure 8:**
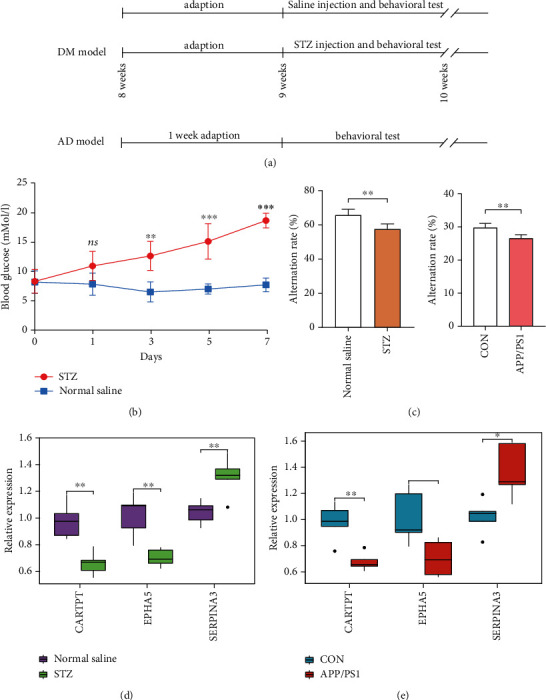
Temporal cortex tissue for external validation: (a) time-flow diagram; (b) changes in blood glucose; (c) results of behavioral test (Y maze); (d) RT-qPCR for CARTPT, EPHA5, and SERPINA3 (*n* = 5 in the control mice; *n* = 5 in the T2DM mice); and (e) RT-qPCR for CARTPT, EPHA5, and SERPINA3 (*n* = 5 in the control mice; *n* = 5 in the APP/PS1 mice). The significance of differences indicated in figures: ^∗^*P* < 0.05, ^∗∗^*P* < 0.01, and ^∗∗∗^*P* < .001.

The flow of the animal experiment is shown in Figure 8(a). After STZ injection within 1 week, the level of random blood glucose in C57BL/6 mice was significantly increased (Figure 8(b); >16.7 mmol/L; *P* < 0.05) when compared with the controls (normal saline injection), indicating that STZ treatment successively induced diabetic model. Besides, the percentage of correct alternation arm was significantly decreased in the diabetic and APP/PS1 mice when compared with the controls (P <0.05, Figure 8(c)), signifying that diabetic and 8-month-old APP/PS1 mice had already developed memory impairment.

### 3.7. RT-qPCR

Following our successfully constructed animal models, RT-qPCR was conducted to finally verify the specific genes in the mice temporal cortex of T2DM and AD. In Figure 8(d), CARTPT and EPHA5 showed a significant decrease (*P* < 0.05), while the expression of SERPINA3 statistically increased (*P* < 0.05) in STZ group when compared with the control. In the aspect of AD model (Figure 8(e)), CARTPT and EPHA5 decreased significantly (*P* < 0.05), while the expression of SERPINA3 was significantly enhanced (*P* < 0.05). Based on these data, we judge that the previous speculations are reliable.

### 3.8. ceRNA Network

**Figure 9:**
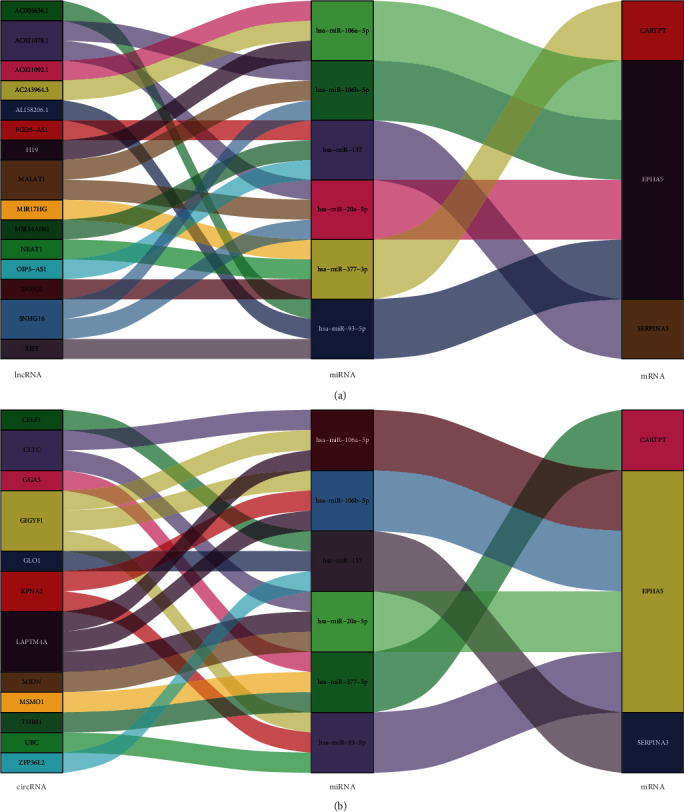
Sankey diagram for the ceRNA network of CARTPT, EPHA5, and SERPINA3. (a) lncRNA-miRNA-mRNA network and (b) circRNA-miRNA-mRNA network.

In miRSystem database, hsa-miR-377-3p was predicted to interact with CARTPT by 3 programs. In StarBase database, hsa-miR-20a-5p, hsa-miR-93-5p, hsa-miR-106a-5p, and hsa-miR-106b-5p were, respectively, predicted to interact with EPHA5 by 6 programs; hsa-miR-137 was predicted to interact with SERPINA3 by 4 programs. To better comprehend the regulation, we further constructed 2 ceRNA networks based on the StarBase database. The simplified lncRNA-miRNA-mRNA network and circRNA-miRNA-mRNA network are, respectively, exhibited in Figures 9(a) and 9(b).

### 3.9. Prediction of the Potential Drugs

DGIdb was utilized to quest the possible pharmaceutical compounds. Briefly, 4 compounds (amphetamine, insulin, dexamethasone, and progesterone) were recognized to interplay with CARTPT; the potential agents of EPHA5 may include vandetanib, hesperadin, and paclitaxel. Unfortunately, no drugs for SERPINA3 have been predicted. More details are shown in Table 1.

### 3.10. Construction of the Nomogram Model

**Figure 10:**
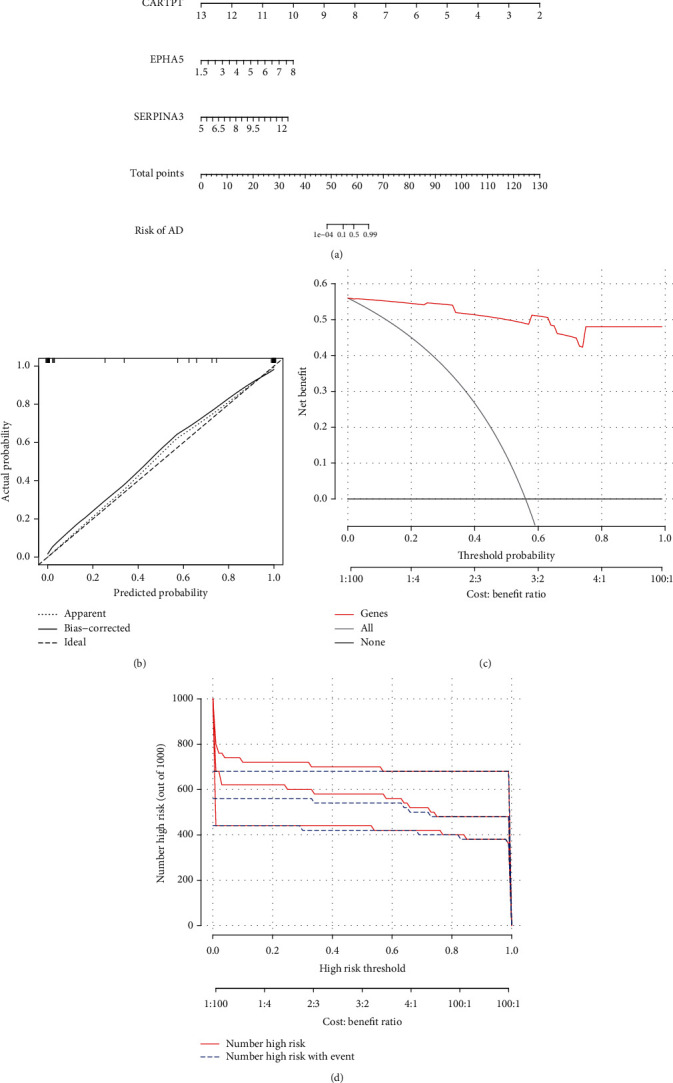
The nomogram model based on the gene expression in GSE122063 (AD). (a) The nomogram, (b) the calibration curve, (c) the DCA curve, and (d) the clinical impact curve.

Using the “rms” package (version 6.2-0) in R (version 4.0.2), a nomogram model based on the 3 specific genes (CARTPT, EPHA5, and SERPINA3) was constructed to predict the risk of Alzheimer's disease (Figure 10(a); GSE122063). As shown in Figure 10(b), the calibration curve suggested a high predictive accuracy of the nomogram model. From 0 to 1 on the abscissa (Figure 10(c)), the red line in the DCA curve is far from and consistently above the gray and black lines, manifesting that decision-making based on the nomogram model may benefit AD patients. At last, we evaluated the clinical impact of the nomogram model through a clinical impact curve (Figure 10(d)).

### 3.11. Hallmark Gene Sets and Immune Cell Infiltration

**Figure 11:**
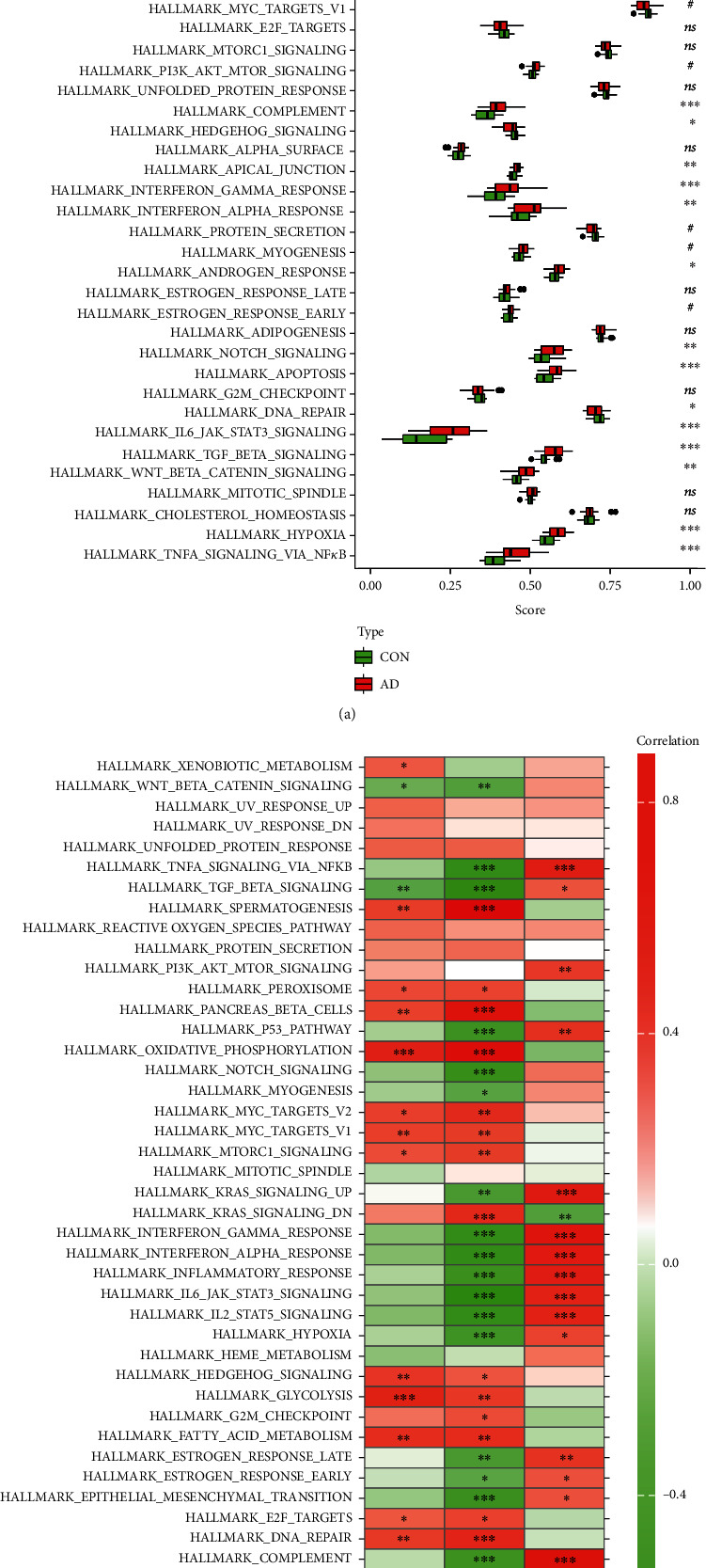
Analysis of hallmark gene sets associated with AD (GSE122063): (a) the specific distribution of the 50 hallmark gene sets in AD and (b) the correlation analysis of the 50 hallmark gene sets with CARTPT, EPHA5, and SERPINA3.

**Figure 12:**
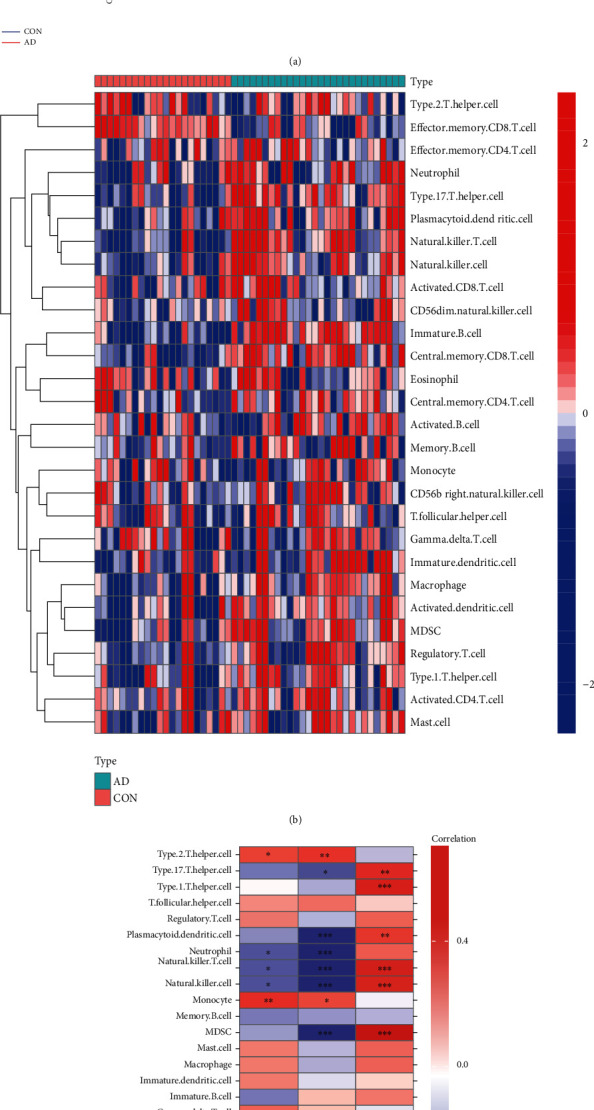
Analysis of immune landscape associated with AD (GSE122063) (a) violin plot: 14 types of immune cells were differently distributed between healthy control and AD (b) heatmap; (c) the relationship between 3 genes (CARTPT, EPHA5, and SERPINA3) and immune cell infiltration.

To further assess the differences in the hallmark gene sets and the immune cell infiltration between controls and AD, the ssGSEA algorithm was employed. The detailed distribution of the 50 hallmark gene sets between AD and control (GSE122063) was illuminated in Figure 11(a) (the significance in the figure as follows: ^ns^*P* < 1, ^#^*P* < 0.2, ^∗^*P* < 0.05, ^∗∗^*P* < 0.01, and ^∗∗∗^*P* < .001). In addition, the infiltration of 28 immune cells between the two groups is shown in Figures 12(a) and 12(b). Briefly, we can find that there are 14 differentially infiltrating immune cells between AD and control groups; they are activated dendritic cell, immature B cell, immature dendritic cell, MDSC, macrophage, natural killer T cell, natural killer cell, neutrophil, plasmacytoid dendritic cell, regulatory T cell, type 1 T helper cell, type 17 T helper cell, central memory CD8 T cell, and effector memory CD8 T cell.  Figure 11(b) shows the correlation of the hallmark gene sets with the specific genes (CARTPT, EPHA5, and SERPINA3), and Figure 12(c) shows the details of their related immune cells. *P* < 0.05 was considered statistically significant. We can find that CARTPT and EPHA5 are generally consistent, while SERPINA3 has the opposite. For instance, both CARTPT and EPHA5 are negatively correlated with the HALLMARK_APICAL_JUNCTION, but SERPINA3 is positively correlated with that; both CARTPT and EPHA5 are negatively correlated with the natural killer T cell, but SERPINA3 is positively correlated with that. These data will help us further appreciate the critical role of the specific genes.

## 4. Discussion

Alzheimer's disease is a neurodegenerative disease with insidious progression [43]. Despite nearly 100 years of research on it, the etiology, pathogenesis, and risk factors are far from being elucidated, which has also led to repeated setbacks in AD drug development. Therefore, finding the risk factors affecting AD, identifying AD high-risk populations, and intervening in novel therapeutic targets have become hot spots in AD research. Recent studies have shown that diabetes can accelerate the decline of executive function in patients [44, 45]. This impairment is significantly related to the time of suffering from diabetes and the level of aging glycosylated hemoglobin in the blood [46]. Due to the lack of a complete cure, it is urgent to reduce the risk of dementia. Understanding how diabetes affects cognition through which targets, and taking early interventions to delay its progression, is of great help to current research.

This time, our GEO-based research discovered 62 DEGs overlapping in the dataset of GSE122063 (AD) and GSE161355 (T2DM) by integrated bioinformatics. The resulting GO enrichments indicated that these genes were closely related to the synaptic function. Synapses are important mediators for maintaining connections between neurons, and their function and structure can change with the activity of neurons, that is, synaptic plasticity [47]. The loss of neurons and the destruction of synaptic plasticity in the brain are the key to cognitive defects. Studies have reported that the volume of the hippocampal CA1 region was significantly reduced in the streptozotocin-induced diabetic rats [48], accompanied by a decrease in the number of spinophilin-/neurabin II-positive cells; in addition, ultrastructural observations revealed widening of the synaptic cleft and reduction of vesicles, along with atrophy, cristae rupture, and ruffling of mitochondrial chromatin and nuclei [49]. Therefore, targeted regulation of synaptic plasticity may be an important mechanism of diabetes-related cognitive impairment [50].

As we all know, insulin can enter the CNS through the blood-cerebrospinal fluid barrier, regulating brain glucose metabolism and the brain structural plasticity to improve memory [51]. To exert this function, insulin mainly activates the mitogen-activated protein kinase (MAPK) [52] and phosphatidylinositol 3-kinase (PI3-K)/Akt [53] signaling pathways. In our pathway enrichment analysis, MAPK signaling (hsa04010) ranked at the top. MAPKs are a group of serine-threonine protein kinases that can be activated by diverse extracellular stimuli. Insulin resistance (IR) can cause strong MAPK immunoreactivity, leading to tau hyperphosphorylation, and a positive correlation between the number of MAPKs and tau protein accumulations was found in transgenic mice accompanied by hyperphosphorylated tau [54]. All this evidence points to the unique role of MAPK signaling in diabetes-related cognitive impairment, and the involved genes are equally worthy of our attention.

Among the overlapping 62 dysregulated genes, we finally identified 3 targets by the machine learning algorithm; they are CARTPT, EPHA5, and SERPINA3. To be specific, we found that CARTPT (logFC: -2.84) and EPHA5 (logFC: -1.18) were significantly downregulated in AD (GSE122063), and they had a good performance as a marker of the disease (GSE122063; the AUC of CARTPT was 0.969; the AUC of EPHA5 was 0.739). On the other side, the expression of SERPINA3 (logFC: 1.82) in samples from AD individuals was higher than that of controls (GSE122063). Likewise, its ROC curve performed well (the AUC of SERPINA3 was 0.886). After further calculation of the AD validation set (GSE5281), we spotted the same trend in the expression of these genes. Similarly, CARTPT (logFC: -1.09) and EPHA5 (logFC: -1.02) were downregulated in T2DM (GSE161355); the AUC of them was 1.00 and 0.933, respectively. As for SERPINA3, the expression of it (logFC: 2.76; GSE161355) in T2DM samples was also higher than the controls (the AUC of its ROC curve was 0.967). We further successfully constructed diabetic and AD animal models, after which brain tissue samples were collected and RT-qPCR was performed to verify our bioinformatics-based predictions. The experimental results (the mRNA level of CARTPT and EPHA5 was significantly decreased, while SERPINA3 increased; *P* < 0.05) were consistent with the previous analyses. Therefore, we concluded that CARTPT, EPHA5, and SERPINA3 might constitute a molecular bridge between T2DM and AD.

According to the ROC curve of the three genes (Figure 7), it is not difficult to recognize that CARTPT has the highest diagnostic efficacy, whether in diabetes or AD (the AUC of CARTPT was 0.969 and 1.00, respectively, in GSE122063 and GSE161355). More than this, CARTPT is also the core gene of the constructed PPI network (Figures 4(b) and 4(c)). We speculate that the role of the CARTPT in the transformation of diseases may be relatively more significant. Also as a metabolic disorder, middle-aged obesity may increase the risk of AD, and CARTPT was now identified as a target for antiobesity drugs, having a high value in connecting obesity and AD [55]. This provides a reference for us to study the relationship between diabetes and AD. CARTPT is capable of encoding the CART protein [56]. CART, fully known as the cocaine- and amphetamine-regulated transcript, is an endogenous neuropeptide, broadly expressed in the CNS [57]. It has also been documented that the expression of CART is decreased in the CSF of AD patients, and the treatment of exogenous CART can partially ameliorate the deficits of learning and memory in mice [58] by improving the synaptic ultrastructure [59]. This evidence fits well with our enrichment analysis and experimental results. Therefore, we believe that CARTPT has the potential to be a target for metabolic-related neurodegenerative changes.

Eph family proteins include Eph receptors with ephrin ligands and are mainly expressed in the CNS [60]. They bidirectionally regulate synaptic signal transmission with neuronal morphogenesis and participate in neural functions such as learning and memory [61]. EphA5 is mainly involved in the formation of dendritic spines, and EphA5 knockout mice exhibit abnormal dendritic spine morphology and neuronal aggregation in the cerebral cortex [62]. The experiments found that the use of EphA5 receptor agonists was able to improve spatial memory in mice [63]. As an acute phase response protein, SERPINA is thought to be a major component of neuritic plaques in the brain, which promotes the assembly of amyloid and its deposition, affecting individual cognition [64]. Their phenotype in the cognitive impairment is consistent with our findings. Nevertheless, the specific relationship between the 2 specific genes and glucose metabolism is also not clear so far. Therefore, EPHA5 and SERPINA3 were equally valuable in subsequent studies.

The relatively small sample size used for bioinformatics analysis and experimental validation in this study may limit our final conclusions to a certain extent. Consequently, the potential mechanisms for glucose metabolism in AD etiology deserve future investigation.

## 5. Conclusion

Gene expression data involving AD and type 2 diabetes were downloaded from the public GEO database platform and subjected to a comprehensive bioinformatics analysis with machine learning algorithms in our study, presenting us with the DEGs linking AD and T2DM. The subsequent enrichment and network analysis about these genes conveyed us their biological functions. Through external dataset validation, as well as construction of animal models, collection of brain tissues, and further verification by RT-qPCR, we located and captured the crucial targets among the DEGs. They are, respectively, CARTPT, EPHA5, and SERPINA3, which are perhaps of great value in studying the molecular regulatory mechanisms shared by type 2 diabetes and Alzheimer's disease. The key factors regulating them, such as miRNA and drugs, as well as the clinical prediction and diagnostic value in type 2 diabetes and Alzheimer's disease, were further analyzed. Our finding may shed new light on the treatment of Alzheimer's disease or diabetic cognitive impairment, but follow-up studies still need to be unfolded.

## Figures and Tables

**Table 1:** The potential drugs targeting the specific genes based on the DGIdb.

Gene	Drug	Sources	PMIDs	Query score	Interaction score
CARTPT	AMPHETAMINE	TdgClinicalTrial	15597110, 15661821, 15680473, 16713658, 15644956, and 15680478	2.04	7.43
	INSULIN	NCI	12883265	0.17	0.62
	DEXAMETHASONE	NCI	12591118	0.12	0.44
	PROGESTERONE	NCI	18598674	0.1	0.38
EPHA5	VANDETANIB	ChemblInteractions	/	0.23	1.09
	HESPERADIN	DTC	19035792	0.11	0.53
	PACLITAXEL	PharmGKB	26133776, 22843789, 26763541, and 26133777	0.08	0.39
SERPINA3	/	/	/	/	/

## Data Availability

The public gene data (GSE161355, GSE122063, and GSE5281) analyzed in this article were downloaded from the NCBI-GEO database (https://www.ncbi.nlm.nih.gov/geo/).

## Abbreviations

AD: Alzheimer's disease
T2DM: Type 2 diabetes mellitus
CNS: Central nervous system
GEO: Gene Expression Omnibus
DEGs: Differentially expressed genes
GO: Gene Ontology
MF: Molecular function
BP: Biological process
CC: Cellular component
KEGG: Kyoto Encyclopedia of Genes and Genomes
PPI: Protein-protein interaction
STRING: Search Tool for the Retrieval of Interacting Genes
LASSO: Least absolute shrinkage and selection operator
SVM-RFE: Support vector machine-recursive feature elimination
CTD: Comparative Toxicogenomics database
ROC: Receiver operating characteristic
STZ: Streptozocin.

## Supplementary Materials

Supplementary 1 Supplementary Table 1: primer pairs for RT-qPCR in mice temporal cortex.

Supplementary 2 Supplementary file 1: the DEGs identified in patients with T2DM compared with controls (GSE161355; a total of 1508 DEGs; including 1473 downregulated genes and 35 upregulated genes).

Supplementary 3 Supplementary file 2: the DEGs identified in patients with AD compared with controls (GSE122063; a total of 788 DEGs; including 475 downregulated genes and 313 upregulated genes).

Supplementary 4 Supplementary Figure 1: the heatmap for the expression level of 62 overlapping genes in GSE122063.

Supplementary 5 Supplementary Figure 2: the expression value of COL24A1 validated in the GSE5281 (no significance).

Supplementary 6 Supplementary Figure 3: the expression value of LY96 validated in the GSE5281 (no significance).
