# Supplementary material for: Identification and Experimental Validation of Marker Genes between Diabetes and Alzheimer's Disease
Source: Oxid Med Cell Longev. 2022 Aug 12;2022:8122532. doi: 10.1155/2022/8122532 (PMC9391608; doi:10.1155/2022/8122532)
Supplement: Supplementary 1 — Supplementary Table 1: primer pairs for RT-qPCR in mice temporal cortex. [file 8122532.f1.docx]

**Supplementary table 1** Primer pairs for RT-qPCR in mice temporal cortex

| **Gene** | **Forward primer (from 5’ to 3’)** | **Reverse primer (from 5’ to 3’)** |
| --- | --- | --- |
| CARTPT | GCTGCTACTGCTACCTTTGC | CTTGCAACGCTTCGATCAGC |
| EPHA5 | CTGCTACTCTGCACCTCGAC | CACCAATCTCTTCCCACCCA |
| SERPINA3 | GGCTCTTGATGGCTGGGATC | TGTAGGAGGTGCCCAAAGCC |
| GAPDH | ATCACTGCCACCCAGAAGAC | ACACATTGGGGGTAGGAACA |
