# Supplementary material for: Identification and Experimental Validation of Marker Genes between Diabetes and Alzheimer's Disease
Source: Oxid Med Cell Longev. 2022 Aug 12;2022:8122532. doi: 10.1155/2022/8122532 (PMC9391608; doi:10.1155/2022/8122532)
Supplement: Supplementary 2 — Supplementary file 1: the DEGs identified in patients with T2DM compared with controls (GSE161355; a total of 1508 DEGs; including 1473 downregulated genes and 35 upregulated genes). [file 8122532.f2.pdf]

Gene	logFC	AveExpr	t	P.Value	adj.P.Val	B
NACAP1	-2.454473	8.1697647	-14.74807	5.75E-10	8.38E-06	12.605671
LOC439938	-2.446467	3.5077813	-14.35946	8.20E-10	8.38E-06	12.317795
MOP-1	-2.047396	2.8611512	-13.64279	1.61E-09	1.10E-05	11.759295
CTDP1	-2.107118	6.2610536	-12.9133	3.32E-09	1.70E-05	11.151422
DNAAF2	-2.195586	3.5784863	-12.51279	5.00E-09	2.05E-05	10.799562
LOC1019285	2.2856235	5.0888377	12.005592	8.56E-09	2.76E-05	10.334348
YEATS4	-2.865605	3.3029557	-11.91318	9.45E-09	2.76E-05	10.247136
N4BP2L2-IT	-2.093214	3.5880967	-11.71554	1.17E-08	3.00E-05	10.057984
STAR	-2.275345	5.0452363	-11.55561	1.40E-08	3.18E-05	9.9022731
FADD	-1.978662	5.6898007	-11.16293	2.18E-08	4.46E-05	9.5095583
LOC1005073	-1.935001	4.9166832	-10.72394	3.63E-08	6.40E-05	9.0523827
GOLGA6L6	-2.002631	3.1922826	-10.69551	3.75E-08	6.40E-05	9.0220976
DCAF12L2	-1.691459	3.4355548	-10.3479	5.69E-08	8.96E-05	8.6448119
CCDC115	-1.860609	4.7790026	-10.07992	7.91E-08	0.0001155	8.3449813
TSSC1	-2.298641	4.9716805	-10.0065	8.66E-08	0.0001181	8.2614395
PIM2	-1.910164	5.3237066	-9.812558	1.10E-07	0.0001398	8.0378045
LINC01352	-1.706496	3.0385361	-9.773148	1.16E-07	0.0001398	7.9918314
SKA3	-2.104495	3.9834602	-9.575877	1.49E-07	0.0001699	7.7589737
LOC1019285	-1.978311	4.6025174	-9.484256	1.68E-07	0.0001736	7.6492573
LRRC55	-2.080674	4.6044493	-9.477844	1.70E-07	0.0001736	7.6415414
SYT6	-1.629629	4.4666521	-9.381833	1.92E-07	0.0001874	7.5254112
DCLK3	-1.84255	3.1608347	-9.319764	2.09E-07	0.000194	7.4497412
PIGH	-2.388039	4.8041261	-9.242987	2.31E-07	0.0002054	7.3554864
ZNF587	-1.481155	4.6349995	-9.094621	2.81E-07	0.0002398	7.1712812
FAM58A	-2.079039	6.9488971	-9.05742	2.96E-07	0.0002419	7.124664
ZNF85	-1.533173	3.891416	-9.000657	3.19E-07	0.0002468	7.0531964
NPY	-2.481415	6.3248128	-8.985489	3.26E-07	0.0002468	7.0340302
NUBPL	-1.962829	3.4915678	-8.888625	3.71E-07	0.0002714	6.9109431
USP44	-1.401011	3.0652096	-8.80308	4.17E-07	0.0002836	6.8012405
MBIP	-2.131248	4.0224884	-8.796122	4.21E-07	0.0002836	6.7922762
MGAM	2.1475279	4.8604245	8.7657088	4.39E-07	0.0002836	6.7530199
FAM53C	-1.547178	7.2607652	-8.716656	4.70E-07	0.0002836	6.6894517
LOC219690	-1.402636	3.3410546	-8.701272	4.80E-07	0.0002836	6.6694506
LOC1005062	-1.544994	4.1814762	-8.697098	4.83E-07	0.0002836	6.6640178
FFAR4	-1.286019	2.5250502	-8.693951	4.85E-07	0.0002836	6.6599211
SPG7	-1.647135	4.0716266	-8.6097	5.45E-07	0.0003099	6.549755
LINC00339	-2.304237	5.0064286	-8.580032	5.68E-07	0.0003143	6.5107398
EIF3H	-1.385451	7.6218572	-8.538919	6.02E-07	0.0003195	6.4564806
CDH13	-1.822223	4.7424344	-8.524866	6.14E-07	0.0003195	6.4378821
DYRK3	-1.288252	4.2585008	-8.512567	6.25E-07	0.0003195	6.4215833
MNS1	-1.700984	4.8172498	-8.481979	6.52E-07	0.0003231	6.3809618
KCNQ1-AS1	-1.438604	3.214181	-8.469929	6.63E-07	0.0003231	6.3649252
LINC01315	-1.60405	3.7948407	-8.387635	7.45E-07	0.0003533	6.2548838
MELK	1.8488518	3.8467402	8.3736966	7.60E-07	0.0003533	6.2361559
ARL14EP	-2.359666	5.5234236	-8.291598	8.54E-07	0.0003883	6.1253127
CYP4F62P	-1.604127	3.5798908	-8.232383	9.30E-07	0.0004032	6.0447966
KIAA1804	-2.052099	4.407425	-8.223685	9.41E-07	0.0004032	6.0329299
TBC1D8B	-1.337498	3.0323335	-8.22043	9.46E-07	0.0004032	6.0284862
NPY1R	-2.633893	4.362559	-8.141439	1.06E-06	0.0004093	5.9202062
UBE2E2	-1.871418	7.5529872	-8.135047	1.07E-06	0.0004093	5.9114062

LOC642776	-2.006008	5.7097359	-8.127355	1.08E-06	0.0004093	5.9008093
ARMC2	-2.046836	6.8841931	-8.12723	1.08E-06	0.0004093	5.9006369
RNF169	-1.535564	6.3006384	-8.126369	1.08E-06	0.0004093	5.899451
FSIP2	-1.466784	2.8877109	-8.123633	1.09E-06	0.0004093	5.8956787
LOC1019286	-1.599973	3.0795038	-8.103327	1.12E-06	0.0004093	5.8676553
BCL2L10	-1.328642	4.3468625	-8.103191	1.12E-06	0.0004093	5.8674677
KDELC2	1.3406951	4.4217485	8.0450921	1.22E-06	0.0004343	5.78697
COL23A1	-1.648126	4.3966561	-8.038393	1.23E-06	0.0004343	5.7776584
DISP1	-1.595352	5.7163059	-8.026219	1.25E-06	0.0004346	5.7607202
TAS2R13	1.4057802	3.46816	8.0026321	1.30E-06	0.0004404	5.7278444
NUDT9P1	-1.364111	4.0291955	-7.994373	1.31E-06	0.0004404	5.7163141
DSCAM-AS1	-1.573116	2.8300914	-7.92243	1.46E-06	0.0004767	5.615477
SPAG17	-1.241513	3.568962	-7.918489	1.47E-06	0.0004767	5.6099322
MAGEB3	-1.82586	3.5051588	-7.904488	1.50E-06	0.0004791	5.5902158
FAM76A	-1.766969	4.6090332	-7.890059	1.53E-06	0.0004819	5.5698678
APCS	-1.327921	4.0795766	-7.833275	1.67E-06	0.0005162	5.4895055
NUPL2	-2.539651	6.5481481	-7.792135	1.77E-06	0.0005398	5.4309985
AICDA	-1.238651	4.1632649	-7.783178	1.79E-06	0.0005398	5.4182284
LOC1005072	-1.529746	3.6592636	-7.724348	1.96E-06	0.0005808	5.3340712
LOC1019287	-1.523363	4.325615	-7.676528	2.10E-06	0.0006113	5.2652994
PTOV1	-1.669684	8.0996554	-7.671332	2.12E-06	0.0006113	5.2578082
ADAM3A	-1.253505	3.4050913	-7.659139	2.16E-06	0.0006139	5.2402109
USP43	-1.514861	5.7586687	-7.627338	2.27E-06	0.0006259	5.1942175
ZNF622	-1.82575	7.6406874	-7.614824	2.31E-06	0.0006259	5.1760788
APH1A	-1.210888	5.586509	-7.611508	2.32E-06	0.0006259	5.1712686
IGF2R	-1.388451	5.4328469	-7.608668	2.33E-06	0.0006259	5.1671466
SLC36A4	-1.488137	3.9704746	-7.59281	2.39E-06	0.0006259	5.1441146
ZNF426	-1.56627	3.0088961	-7.582784	2.42E-06	0.0006259	5.1295341
TRAPPC3L	-1.727257	3.4478933	-7.580302	2.43E-06	0.0006259	5.1259218
PTGER2	-1.352471	2.8716543	-7.563879	2.50E-06	0.0006259	5.1020015
SMKR1	-1.73283	3.6856642	-7.561676	2.50E-06	0.0006259	5.0987896
LOC1001291	-1.660899	4.9058933	-7.560366	2.51E-06	0.0006259	5.0968802
CYP4F2	-1.409895	4.0165134	-7.518184	2.67E-06	0.0006525	5.0352393
MSANTD2	-1.979865	3.9210032	-7.517206	2.68E-06	0.0006525	5.0338071
LOC1019271	-1.513929	4.1026203	-7.497596	2.76E-06	0.0006644	5.005061
MRGPRX2	-1.319505	3.1392515	-7.469314	2.88E-06	0.0006857	4.9635035
PPP3CB-AS1	-1.377677	4.134453	-7.429313	3.06E-06	0.0007207	4.9045287
DPP10-AS3	-1.157418	3.1947404	-7.408976	3.16E-06	0.0007351	4.8744561
KLF10	-1.607596	3.6586215	-7.401589	3.20E-06	0.0007352	4.8635179
GDF10	-1.580297	3.960122	-7.369171	3.36E-06	0.0007642	4.815421
ADTRP	-2.220043	4.1663055	-7.338797	3.52E-06	0.0007922	4.7702166
SLC4A10	-2.018697	4.9404554	-7.32863	3.58E-06	0.000796	4.7550548
USP19	-1.509596	5.4259693	-7.318985	3.63E-06	0.0007993	4.7406591
BC040901 /	-1.303756	2.592894	-7.300519	3.74E-06	0.0008137	4.7130574
LYSMD1	-1.457894	5.3791806	-7.28499	3.83E-06	0.0008213	4.6898059
LOC1019289	-1.3281	3.8478338	-7.279388	3.86E-06	0.0008213	4.6814105
C1orf159	-1.512073	3.7654921	-7.274384	3.89E-06	0.0008213	4.6739064
GS1-600G8.	-1.224323	3.1051832	-7.258461	3.99E-06	0.0008255	4.6500046
CLUL1	-1.56859	3.9204808	-7.253213	4.02E-06	0.0008255	4.6421179
ZNF354A	-1.420113	5.4911417	-7.240235	4.11E-06	0.0008255	4.6225994
FREM1	-1.326303	4.0573944	-7.22452	4.21E-06	0.0008255	4.5989308

LINC00629	-1.412531	3.1375473	-7.224215	4.21E-06	0.0008255	4.5984701
FAM81B	-1.132248	2.5968614	-7.223064	4.22E-06	0.0008255	4.5967358
ZNF674-AS1	-1.329524	3.1228723	-7.22305	4.22E-06	0.0008255	4.5967143
PTPRK	-1.568527	6.1734863	-7.213002	4.28E-06	0.0008255	4.5815602
C5orf64	-1.259321	3.4670102	-7.204076	4.35E-06	0.0008255	4.568085
TMEM57	-1.287626	6.3541088	-7.203988	4.35E-06	0.0008255	4.5679526
YPEL1	-1.238006	6.8142845	-7.197693	4.39E-06	0.0008255	4.5584415
LINC00669	-1.156945	2.4399659	-7.196359	4.40E-06	0.0008255	4.5564257
CSTF2	-1.20496	5.4344852	-7.153455	4.70E-06	0.00087	4.4914421
C21orf15	-1.468119	5.6068686	-7.151329	4.72E-06	0.00087	4.4882155
LINC01234	-1.333173	3.3306979	-7.133683	4.85E-06	0.0008865	4.4614047
LOC10192960	-1.200892	2.3227702	-7.104091	5.08E-06	0.0009166	4.4163383
SLC25A17	-1.220318	5.0582862	-7.098466	5.13E-06	0.0009166	4.4077563
SLC25A38	-1.951665	5.4163437	-7.095728	5.15E-06	0.0009166	4.4035782
MXD3	-1.126088	5.6760469	-7.079217	5.29E-06	0.0009327	4.3783565
SLC22A4	-1.389489	3.4553497	-7.073029	5.34E-06	0.0009338	4.3688934
LOC1005060	-1.471548	2.7512149	-7.062317	5.43E-06	0.0009393	4.3524979
LOH12CR2	-1.256466	3.9277213	-7.057002	5.48E-06	0.0009393	4.3443572
LINC01097	-1.492221	3.2652415	-7.052376	5.52E-06	0.0009393	4.3372675
LOC153546	-1.286334	5.5101952	-7.048104	5.56E-06	0.0009393	4.3307193
SPIN4	-2.467554	3.7562561	-7.038183	5.64E-06	0.0009464	4.3154975
LOC1019277	-1.622156	5.0692317	-6.981328	6.18E-06	0.0010128	4.2279893
LOC1019278	-1.117587	2.8074065	-6.98122	6.18E-06	0.0010128	4.2278239
CSK	-1.280078	5.9373351	-6.97755	6.21E-06	0.0010128	4.2221584
SLC25A15	-1.112369	4.4313658	-6.974668	6.24E-06	0.0010128	4.217708
SOX17	-1.60581	3.6231216	-6.970324	6.29E-06	0.0010128	4.2109974
MUC7	-1.258372	3.2463359	-6.954523	6.45E-06	0.0010227	4.1865649
FRMD6-AS1	-1.133521	3.716731	-6.954473	6.45E-06	0.0010227	4.1864885
DCP1B	-1.167012	4.4563435	-6.931585	6.69E-06	0.0010526	4.1510316
DSTNP2	-1.473977	7.9564623	-6.915074	6.87E-06	0.0010726	4.1254061
LOC388456	-1.379322	3.2534533	-6.902409	7.01E-06	0.0010812	4.1057209
ARHGAP12	-1.62191	4.0191993	-6.898325	7.05E-06	0.0010812	4.0993692
ERVH-4	-1.489274	2.9092826	-6.892821	7.12E-06	0.0010812	4.0908036
ISY1	-1.162867	4.9793714	-6.891356	7.13E-06	0.0010812	4.088522
TEX30	-1.292889	5.414519	-6.880718	7.26E-06	0.0010918	4.0719523
RBM10	-1.176405	6.7402306	-6.873153	7.35E-06	0.0010937	4.0601576
ALOX15	-1.342374	2.8044116	-6.870515	7.38E-06	0.0010937	4.0560435
THAP7-AS1	-1.155009	3.9918103	-6.855273	7.56E-06	0.0011098	4.0322484
CHRNA1	-1.142984	3.4661425	-6.852531	7.59E-06	0.0011098	4.0279645
CCDC62	-1.175781	3.0518047	-6.838999	7.76E-06	0.0011262	4.0068053
PCDHB16	-1.739763	3.2378312	-6.831752	7.85E-06	0.0011315	3.9954614
TYRO3P	-1.293749	5.1403861	-6.823902	7.95E-06	0.0011379	3.9831646
CNTNAP1	-1.770745	8.3795413	-6.805903	8.19E-06	0.0011546	3.9549366
ASB5	-1.126337	2.1668746	-6.805423	8.19E-06	0.0011546	3.9541827
NLGN4Y	-2.321073	3.032104	-6.802041	8.24E-06	0.0011546	3.9488731
PIGN	-1.269754	4.331165	-6.794432	8.34E-06	0.001161	3.9369215
OR7C2	-1.537155	3.2299294	-6.773593	8.63E-06	0.0011927	3.9041426
ZSWIM5	-1.199988	5.5786374	-6.757188	8.86E-06	0.0012127	3.8782912
PKDCC	-1.298996	6.3381251	-6.751214	8.95E-06	0.0012127	3.8688675
CBFA2T3	-1.289445	6.329906	-6.751017	8.95E-06	0.0012127	3.8685573
GEN1	-1.351757	3.326237	-6.737792	9.14E-06	0.0012193	3.8476751

MRPL36	-1.63813	6.6141675	-6.736154	9.17E-06	0.0012193	3.8450869
ZP2	-1.227211	3.8048443	-6.735566	9.18E-06	0.0012193	3.8441583
RASGRP3	-1.116561	3.6395447	-6.722119	9.38E-06	0.0012359	3.822895
RNASEH1-AS	-1.137854	5.0463944	-6.719336	9.42E-06	0.0012359	3.8184896
C20orf194	-1.010661	7.8271796	-6.704953	9.65E-06	0.0012572	3.7957104
FNDC3B	-1.193148	2.4566066	-6.683783	9.99E-06	0.0012874	3.7621249
PSD	-1.664388	7.6817292	-6.682682	1.00E-05	0.0012874	3.7603769
LOC1019286	-1.271637	2.5013526	-6.674922	1.01E-05	0.0012956	3.748047
CHRM5	-1.728846	4.202253	-6.666291	1.03E-05	0.0013059	3.7343238
COX5B	-1.307481	9.1999964	-6.657039	1.04E-05	0.0013177	3.7195988
LOC1027237	-1.411981	4.8228652	-6.650699	1.05E-05	0.0013233	3.7095026
ADAM17	-1.151009	6.3195144	-6.633215	1.08E-05	0.0013446	3.681626
C1orf204	-1.410424	4.638897	-6.631722	1.09E-05	0.0013446	3.6792437
TRMT12	-1.163644	5.8158421	-6.629838	1.09E-05	0.0013446	3.6762369
FAM196A	-1.520024	2.5422994	-6.610306	1.13E-05	0.0013672	3.6450312
KIAA0408	-1.140069	3.1442568	-6.609255	1.13E-05	0.0013672	3.6433495
RAB33B	-1.424674	4.8161314	-6.605513	1.14E-05	0.0013672	3.637364
PRMT3	-1.570234	2.9705482	-6.605203	1.14E-05	0.0013672	3.6368688
SMCP	-1.216699	4.4959967	-6.571077	1.20E-05	0.0014378	3.582181
CCDC86	-1.37328	6.2407473	-6.562597	1.22E-05	0.0014496	3.5685646
SLC26A4	-1.80974	3.361863	-6.552353	1.24E-05	0.0014545	3.5521008
ALKBH8	-1.333752	3.3838788	-6.551178	1.24E-05	0.0014545	3.5502112
DTX1	-1.065311	6.4470994	-6.550097	1.24E-05	0.0014545	3.5484723
NAF1	-1.946977	3.4657864	-6.546541	1.25E-05	0.0014547	3.5427539
DLG5-AS1	-1.223377	3.7417421	-6.536931	1.27E-05	0.0014697	3.5272859
TUBGCP5	-1.365255	4.7128577	-6.518763	1.31E-05	0.001506	3.4980054
ZBED9	-1.14458	3.7097541	-6.511689	1.33E-05	0.0015153	3.4865917
LMO7DN	-1.222831	3.298756	-6.493899	1.37E-05	0.0015513	3.4578541
LOC1019275	-1.352518	3.4829899	-6.489111	1.38E-05	0.0015513	3.450111
LOC1019291	-1.229336	2.6055958	-6.487512	1.38E-05	0.0015513	3.4475243
FRAT1	-1.141169	5.1405319	-6.477464	1.40E-05	0.0015688	3.4312619
BAALCOS	-1.282736	4.8958995	-6.47387	1.41E-05	0.0015697	3.4254413
RELT	-1.151009	5.741939	-6.469736	1.42E-05	0.0015715	3.418744
ZCWPW2	-1.202191	2.8534852	-6.463472	1.44E-05	0.0015715	3.4085905
AP4E1	-1.2166	4.7135171	-6.46344	1.44E-05	0.0015715	3.4085392
RAB3A	-1.959564	8.6827299	-6.458112	1.45E-05	0.0015748	3.399897
NDUFAF1	-1.430439	5.3851558	-6.455813	1.45E-05	0.0015748	3.3961685
SYTL5	-1.135179	3.241579	-6.443412	1.49E-05	0.0015992	3.3760354
BTBD6	-1.301466	7.5648574	-6.43324	1.51E-05	0.001618	3.3595044
PIN1	-2.027697	6.8774278	-6.426082	1.53E-05	0.0016272	3.3478616
LINC00923	-1.268273	3.2787307	-6.423602	1.53E-05	0.0016272	3.3438267
C10orf67	-1.075027	6.5356826	-6.42005	1.54E-05	0.0016285	3.338045
DARS2	-1.27222	4.3358443	-6.413739	1.56E-05	0.0016302	3.3277688
CIDEC	-1.202122	3.6688183	-6.4133	1.56E-05	0.0016302	3.3270537
ZNF681	-1.031821	3.8610392	-6.403474	1.59E-05	0.001645	3.3110401
LINC01180	-1.385585	4.4046969	-6.401817	1.59E-05	0.001645	3.3083385
KRTAP19-1	-1.066613	2.0724414	-6.397557	1.60E-05	0.0016484	3.3013898
BTBD18	-1.448231	3.6265216	-6.3923	1.62E-05	0.0016547	3.2928129
LOC729083	1.1486812	3.1463899	6.3889222	1.63E-05	0.0016558	3.2872986
TIGD2	-1.666335	3.7631638	-6.377715	1.66E-05	0.0016591	3.268992
TCEAL4	-1.691167	7.3610386	-6.376419	1.66E-05	0.0016591	3.2668738

SCN4A	-1.489934	3.6039796	-6.369539	1.68E-05	0.0016591	3.2556252
TMEM186	-1.160351	5.5723767	-6.367171	1.69E-05	0.0016591	3.2517514
PDZRN3-AS1	-1.827851	4.8445887	-6.363046	1.70E-05	0.0016591	3.2450018
RPS6KA6	-1.098221	4.4710323	-6.361647	1.70E-05	0.0016591	3.2427127
DSCR9	-1.474842	3.8464576	-6.361124	1.70E-05	0.0016591	3.2418552
ENTPD3-AS1	-1.709333	3.8980715	-6.360036	1.71E-05	0.0016591	3.2400749
RALY-AS1	-1.105542	3.4089414	-6.359936	1.71E-05	0.0016591	3.2399109
KDM4D	-1.13626	3.8805101	-6.357626	1.71E-05	0.0016591	3.2361293
NF1	-1.308603	3.0313172	-6.355957	1.72E-05	0.0016591	3.2333959
PCDH10	-1.190453	4.891809	-6.350967	1.73E-05	0.0016641	3.2252231
SH3PXD2A	-1.024801	6.40461	-6.348585	1.74E-05	0.0016641	3.2213205
UBE4B	-1.105989	7.9133551	-6.345664	1.75E-05	0.0016645	3.216533
PRIM2	-1.245146	3.0288035	-6.320555	1.82E-05	0.0017187	3.1753262
WDR5B	-1.113583	5.1966864	-6.319453	1.83E-05	0.0017187	3.1735165
LINC00158	-1.019261	4.6480775	-6.316306	1.84E-05	0.0017187	3.168344
TEX41	-1.054086	2.6835504	-6.313904	1.85E-05	0.0017187	3.1643967
LOC1019282	-1.159531	6.5228875	-6.312234	1.85E-05	0.0017187	3.1616509
LARP7	-1.126423	4.0240987	-6.310263	1.86E-05	0.0017187	3.1584093
PSG5	-1.145458	4.1666254	-6.300461	1.89E-05	0.0017338	3.1422826
LRTOMT	-1.358811	4.35204	-6.298476	1.89E-05	0.0017338	3.1390149
MOK	-1.167641	5.4570171	-6.297106	1.90E-05	0.0017338	3.1367602
SDHAF1	-1.428054	5.8736166	-6.290658	1.92E-05	0.0017449	3.1261397
LOC1019277	-1.598958	4.7262057	-6.28539	1.94E-05	0.0017452	3.117459
MATN1-AS1	-1.450376	4.1538366	-6.280018	1.95E-05	0.0017452	3.1086024
DACT3	-1.094548	6.2191751	-6.278624	1.96E-05	0.0017452	3.1063031
FAM186A	-1.22974	2.9124119	-6.278087	1.96E-05	0.0017452	3.1054182
KIAA1586	-1.386028	3.8178953	-6.277554	1.96E-05	0.0017452	3.1045384
NUB1	-1.06434	5.1480241	-6.261531	2.02E-05	0.0017701	3.0780897
STRIP2	-1.324711	3.5415704	-6.256632	2.03E-05	0.0017712	3.0699951
C21orf128	-1.618927	4.6674347	-6.256096	2.03E-05	0.0017712	3.0691099
KRT84	-1.644054	3.6906825	-6.246364	2.07E-05	0.001793	3.0530177
SPRNP1	-1.073962	3.8362378	-6.224328	2.15E-05	0.0018475	3.0165311
LOC1001304	-1.040757	4.4102025	-6.223148	2.15E-05	0.0018475	3.0145752
MTMR14	-1.245929	5.5215574	-6.221272	2.16E-05	0.0018475	3.0114638
LINC00460	-1.492661	3.6081483	-6.211138	2.20E-05	0.0018718	2.9946553
TRMT10A	-1.013367	2.4480115	-6.20794	2.21E-05	0.0018729	2.9893464
RAP1GAP2	-1.864424	8.4353886	-6.202967	2.23E-05	0.0018745	2.9810908
DEPTOR	-1.137714	3.7596462	-6.189422	2.28E-05	0.0019104	2.9585827
LOC1019295	-1.295861	4.2752662	-6.171944	2.35E-05	0.0019497	2.9294974
CHST2	-1.082141	4.8224845	-6.169028	2.36E-05	0.0019497	2.9246408
LINC01089	-1.074652	4.6647866	-6.166834	2.37E-05	0.0019497	2.9209862
LOC440149	-1.468171	3.5474271	-6.159839	2.40E-05	0.0019497	2.9093272
NDUFB3	-1.5428	7.1946784	-6.158533	2.40E-05	0.0019497	2.9071493
MEIG1	-1.175097	3.3876422	-6.15525	2.41E-05	0.0019529	2.9016751
TPRN	-1.482729	6.2960355	-6.150594	2.43E-05	0.0019608	2.8939071
DAPK2	-1.19704	5.122683	-6.143515	2.46E-05	0.0019768	2.8820922
FAM155B	-1.292758	4.12225	-6.137059	2.49E-05	0.001991	2.8713096
PRKAG2-AS1	-1.246043	5.4420506	-6.122938	2.55E-05	0.0020317	2.8477026
LOC1027233	-1.176823	2.7663561	-6.118346	2.57E-05	0.0020357	2.8400206
ITK	-1.965906	3.3420464	-6.112448	2.60E-05	0.0020357	2.8301466
FSCN1	-1.301606	7.752495	-6.112407	2.60E-05	0.0020357	2.8300779

SF3A3	-1.194595	6.5899999	-6.109382	2.61E-05	0.0020357	2.825013
MYO1H	-1.656058	4.6790879	-6.109335	2.61E-05	0.0020357	2.8249347
IRG1	-1.157216	4.073862	-6.108309	2.62E-05	0.0020357	2.8232166
USP30-AS1	-1.296842	4.2872484	-6.099574	2.66E-05	0.0020415	2.8085796
UBE3D	-1.02072	2.7360475	-6.098258	2.66E-05	0.0020415	2.8063728
NAGK	-1.126398	6.8542383	-6.097668	2.66E-05	0.0020415	2.8053839
CEP135	-1.197	3.5046975	-6.095661	2.67E-05	0.0020415	2.8020188
LMO3	-1.081306	7.0275506	-6.092271	2.69E-05	0.0020458	2.7963341
C6orf195	-1.355524	3.2935282	-6.089169	2.70E-05	0.0020491	2.7911298
PNRC1	-1.93379	6.3139848	-6.078295	2.76E-05	0.00208	2.7728766
CD2AP	-1.160864	4.2160648	-6.071135	2.79E-05	0.0020804	2.7608472
LOC1005059	-1.342057	3.0054684	-6.068015	2.80E-05	0.0020804	2.7556032
TMCC1-AS1	-1.291862	3.1261428	-6.067703	2.81E-05	0.0020804	2.7550785
ACSL4	-1.793572	4.0333833	-6.062614	2.83E-05	0.0020804	2.7465223
LRRC75B	-1.081773	4.9553638	-6.061195	2.84E-05	0.0020804	2.7441351
MRPL44	-1.459034	5.2875784	-6.060121	2.84E-05	0.0020804	2.742329
MOB1B	-1.131788	5.0422311	-6.059091	2.85E-05	0.0020804	2.740596
MASTL	-1.577448	3.317702	-6.058795	2.85E-05	0.0020804	2.7400978
PTH	-1.015534	2.051717	-6.058413	2.85E-05	0.0020804	2.7394558
PNP	1.4118446	4.6212899	6.0571194	2.86E-05	0.0020804	2.7372786
RER1	-1.059535	7.2641324	-6.046466	2.91E-05	0.0021035	2.719344
NR4A2	-1.292445	4.9323149	-6.045688	2.91E-05	0.0021035	2.7180338
ZNF124	-1.561275	3.8904855	-6.044544	2.92E-05	0.0021035	2.7161061
MTMR6	-1.300245	4.723327	-6.037911	2.95E-05	0.0021202	2.70493
LOC1002886	-1.752717	4.284246	-6.032354	2.98E-05	0.0021325	2.6955606
DCK	-2.551514	4.9761957	-6.030495	2.99E-05	0.0021325	2.6924265
GTF3C4	-1.032921	5.4419085	-6.021021	3.04E-05	0.0021601	2.6764403
ZNF702P	-1.421268	2.6622976	-6.016853	3.06E-05	0.0021682	2.6694025
MTCH1	-1.491035	9.7296284	-6.010579	3.10E-05	0.0021767	2.6588045
TSEN2	-1.113178	4.4425458	-6.006768	3.12E-05	0.0021815	2.6523649
POP1	-1.203389	3.9689331	-6.005262	3.12E-05	0.0021815	2.6498189
TPBGL	-1.362841	4.4693177	-6.002846	3.14E-05	0.0021815	2.645735
DEGS1	-1.860481	4.6277383	-5.998521	3.16E-05	0.0021815	2.6384216
LOC339622	-1.172853	2.4028157	-5.997339	3.17E-05	0.0021815	2.6364209
BZRAP1	-1.783479	6.5983374	-5.995562	3.18E-05	0.0021815	2.6334163
SEPT7P9	-1.452791	4.2626216	-5.990604	3.20E-05	0.0021929	2.6250256
EGFL6	-1.094556	2.7309787	-5.985662	3.23E-05	0.0021983	2.6166586
MMP21	-1.100718	2.8306279	-5.98432	3.24E-05	0.0021983	2.6143855
EIF3F	-1.121367	4.3735867	-5.983417	3.24E-05	0.0021983	2.6128562
RABGEF1	-1.459457	4.5012708	-5.980677	3.26E-05	0.0022015	2.6082157
ZFP92	-1.063578	5.6619376	-5.9751	3.29E-05	0.0022155	2.5987647
FOXN3-AS2	-1.198969	2.9366404	-5.960601	3.38E-05	0.0022376	2.5741747
BRE	-1.106894	5.9406107	-5.958675	3.39E-05	0.0022376	2.5709063
BCL7A	-1.118522	5.871726	-5.956934	3.40E-05	0.0022376	2.5679505
NUP133	-1.225303	5.4963362	-5.956089	3.40E-05	0.0022376	2.566516
HBBP1	-1.319845	4.181516	-5.952778	3.42E-05	0.0022376	2.5608949
UHRF1	-1.118537	3.7499222	-5.952547	3.42E-05	0.0022376	2.5605025
FAM126A	-1.041739	3.7975831	-5.937888	3.51E-05	0.002288	2.5355906
PAK1IP1	-1.364161	6.1576183	-5.925647	3.59E-05	0.0023238	2.5147641
WHAMMP2	-1.240106	3.2665715	-5.92531	3.59E-05	0.0023238	2.5141904
LOC646522	-1.384217	3.9059368	-5.898478	3.76E-05	0.0024196	2.46846

ARNTL2	-1.003715	4.7055235	-5.895678	3.78E-05	0.0024238	2.4636821
NELFB	-1.087168	5.6857049	-5.88485	3.85E-05	0.002432	2.4451917
GAB2	-1.133547	7.2200354	-5.880826	3.88E-05	0.0024415	2.4383169
ZC3HAV1L	-1.156406	2.9110925	-5.877074	3.90E-05	0.00245	2.4319034
DEFB1	-1.530638	3.4831091	-5.86177	4.01E-05	0.002501	2.4057234
MAML1	-1.086667	5.5663324	-5.852227	4.08E-05	0.0025109	2.3893808
CPA1	-1.371873	3.1300401	-5.851463	4.08E-05	0.0025109	2.3880728
HDAC2	-1.383736	5.3894028	-5.849046	4.10E-05	0.0025109	2.3839311
MOSPD1	-1.019705	3.4431801	-5.848421	4.10E-05	0.0025109	2.3828606
LOC1019280	-1.191983	3.8769313	-5.847782	4.11E-05	0.0025109	2.3817641
LOC1019297	-1.341089	4.6981285	-5.847441	4.11E-05	0.0025109	2.3811801
ZMAT1	-1.358026	3.9088983	-5.841183	4.16E-05	0.002531	2.3704524
SLC2A1-AS1	-1.352537	3.8235761	-5.833677	4.21E-05	0.0025507	2.3575755
IL13RA2	-2.234433	4.1832894	-5.831687	4.23E-05	0.0025507	2.3541618
NCAM2	-1.605371	4.57893	-5.826642	4.26E-05	0.00256	2.3455015
DGKD	-1.042953	6.4757926	-5.82625	4.27E-05	0.00256	2.3448285
DPP10	-2.332511	5.5147281	-5.816373	4.34E-05	0.0025895	2.3278618
PVRL3	-1.300867	4.4409115	-5.813565	4.36E-05	0.0025915	2.3230357
RAB6B	-1.356283	9.2677222	-5.812614	4.37E-05	0.0025915	2.3214014
PPY	-1.243076	4.2219022	-5.808993	4.40E-05	0.0025931	2.315176
LOC1005067	-1.17361	3.3933071	-5.808985	4.40E-05	0.0025931	2.3151611
GIT1	-1.452622	8.9071078	-5.803504	4.44E-05	0.0026106	2.3057339
PGBD2	-1.233701	4.5639283	-5.797383	4.49E-05	0.0026237	2.2952015
NEURL4	-1.47902	6.6479983	-5.778196	4.64E-05	0.0026983	2.2621479
FBN3	-1.815996	3.9403239	-5.774549	4.67E-05	0.0027069	2.2558578
SLC22A24	-1.551969	3.0837338	-5.773174	4.68E-05	0.0027069	2.2534863
RARS	-1.864904	4.7655541	-5.768908	4.72E-05	0.0027141	2.2461275
PCDHGA8	1.0042373	4.4372951	5.7684579	4.72E-05	0.0027141	2.245351
TMEM91	-1.234791	6.2586508	-5.766052	4.74E-05	0.002718	2.2411991
OPRK1	-1.136248	3.7508546	-5.763981	4.76E-05	0.0027203	2.2376251
RAB3B	-1.36303	4.5490403	-5.756897	4.82E-05	0.0027296	2.2253928
LOC730139	-1.200292	4.5435245	-5.756218	4.83E-05	0.0027296	2.2242195
MREG	-1.706594	5.5356987	-5.735481	5.01E-05	0.0028007	2.1883686
CLK2	-1.453924	7.6867429	-5.721922	5.13E-05	0.0028425	2.1648924
ZNF804A	-1.680899	3.3383811	-5.717167	5.17E-05	0.0028483	2.1566539
BTF3P11	-1.544202	3.2553868	-5.716103	5.18E-05	0.0028483	2.1548087
AGPAT4	-1.01599	6.7991575	-5.714392	5.20E-05	0.0028483	2.1518436
LINC00382	-1.036502	2.20896	-5.713154	5.21E-05	0.0028483	2.1496972
LOC221946	-1.171946	3.5514272	-5.699469	5.33E-05	0.0029012	2.1259589
ADAMTS18	-1.066674	3.7809686	-5.69483	5.38E-05	0.0029034	2.1179047
MGC16025	-1.267986	4.3011335	-5.690801	5.42E-05	0.0029165	2.1109081
RAP2B	-1.058171	5.3883007	-5.687489	5.45E-05	0.0029218	2.1051545
C19orf12	-1.043201	6.6061394	-5.683918	5.48E-05	0.0029255	2.0989489
LNP1	-1.503323	6.5233453	-5.678256	5.54E-05	0.0029433	2.0891079
LOC1019276	-1.344468	3.0510231	-5.672861	5.59E-05	0.0029483	2.0797239
ELL3	-1.06165	5.3751279	-5.670105	5.62E-05	0.0029483	2.0749293
CLDN16	-1.28186	3.838688	-5.669419	5.63E-05	0.0029483	2.0737354
LYZL6	-1.143509	3.8668346	-5.668664	5.63E-05	0.0029483	2.0724234
NFE4	-1.454325	3.6401333	-5.668577	5.63E-05	0.0029483	2.0722719
LINC01362	1.0149544	4.3304465	5.6670001	5.65E-05	0.0029491	2.0695269
PIH1D2	-1.04804	2.5646233	-5.664483	5.68E-05	0.002952	2.0651452

COLEC11	-1.149655	5.5472419	-5.662036	5.70E-05	0.002952	2.0608851
VAPB	-1.189262	7.5843412	-5.647387	5.85E-05	0.003	2.0353654
TTTY15	-1.875928	4.9359455	-5.640687	5.92E-05	0.0030233	2.0236816
IGLL5	-1.097136	5.5142607	-5.640228	5.93E-05	0.0030233	2.0228811
NIT1	-1.29811	4.9690684	-5.637515	5.95E-05	0.0030304	2.0181485
DACH1	-1.13564	4.4691909	-5.632293	6.01E-05	0.0030474	2.0090363
LOC339803	-1.034113	3.6522531	-5.631578	6.02E-05	0.0030474	2.0077879
RBBP5	-1.532362	4.2475519	-5.628336	6.05E-05	0.0030474	2.0021286
ANKS1A	-1.254627	6.3831278	-5.626051	6.08E-05	0.0030474	1.9981373
LINC00610	-1.200195	3.4776681	-5.624598	6.09E-05	0.0030478	1.9956013
TRPV6	-1.092667	6.4873386	-5.614992	6.20E-05	0.0030928	1.9788173
NUAK1	-1.402703	9.6619004	-5.610882	6.24E-05	0.0030984	1.9716317
NDUFA8	-1.200118	7.5966166	-5.609892	6.25E-05	0.0030984	1.9699014
SCARNA2	-1.796819	7.8292065	-5.605937	6.30E-05	0.0031013	1.9629831
PTPRR	-1.873248	4.4634093	-5.603928	6.32E-05	0.0031013	1.9594693
KIR2DS5	-1.166667	4.381717	-5.603709	6.32E-05	0.0031013	1.9590854
THAP5	-1.333048	3.6262946	-5.602725	6.33E-05	0.0031013	1.957364
ANO10	-1.378212	6.8712973	-5.602617	6.34E-05	0.0031013	1.9571747
LOC1001299	-1.126014	3.7192754	-5.600512	6.36E-05	0.0031055	1.953491
LOC1019290	-1.434616	3.6793249	-5.598015	6.39E-05	0.003112	1.9491216
LOC284240	1.0294493	4.1130429	5.5951642	6.42E-05	0.0031204	1.9441311
ADRA2C	-1.211903	7.2190159	-5.592758	6.45E-05	0.0031221	1.9399174
ZCCHC11	-1.088412	3.8150467	-5.589665	6.48E-05	0.0031289	1.934501
LRRD1	-1.009486	2.2347024	-5.581586	6.58E-05	0.0031545	1.9203453
PTH2	-1.191484	4.8422247	-5.579866	6.60E-05	0.0031545	1.9173295
GBP5	-1.168453	3.7906669	-5.569495	6.72E-05	0.003191	1.8991422
LOC1019280	-1.096436	2.5426507	-5.567054	6.75E-05	0.0031976	1.8948591
ARL1	-1.247119	7.1713465	-5.563249	6.80E-05	0.0032024	1.8881811
LOC1019294	-1.044271	3.8969671	-5.559737	6.84E-05	0.0032024	1.8820148
FXYD7	-1.404491	5.0087525	-5.559709	6.84E-05	0.0032024	1.8819671
LOC283214	-1.47775	3.2369087	-5.558378	6.86E-05	0.0032024	1.8796296
PAM16	-1.18903	6.3551556	-5.555387	6.89E-05	0.0032024	1.8743755
GLYATL2	-1.221123	3.9644157	-5.554306	6.91E-05	0.0032024	1.8724777
TMPO-AS1	-1.08166	4.5308386	-5.552821	6.93E-05	0.0032024	1.8698691
LOC1005054	-1.042172	4.5073189	-5.551484	6.94E-05	0.0032024	1.8675195
LONP1	-1.213217	5.6159914	-5.550889	6.95E-05	0.0032024	1.8664738
ANKRA2	-1.773291	3.8702474	-5.54187	7.06E-05	0.0032473	1.8506197
GRAMD1B	-1.144408	9.0738278	-5.538397	7.11E-05	0.0032544	1.844513
UBXN2A	-1.090186	5.8437105	-5.537926	7.11E-05	0.0032544	1.8436849
KRT2	-1.214759	3.2871036	-5.5369	7.13E-05	0.0032544	1.8418784
MMD	-1.382806	7.5134016	-5.534455	7.16E-05	0.0032614	1.8375783
TMPRSS11E	-1.085542	3.568217	-5.531252	7.20E-05	0.0032625	1.8319413
CXXC4	-1.133053	5.7595861	-5.530875	7.20E-05	0.0032625	1.8312782
SLN	-1.383214	5.2960559	-5.527495	7.25E-05	0.0032625	1.8253281
SHISA8	-1.001386	3.6041099	-5.527382	7.25E-05	0.0032625	1.8251297
LOC1019290	-1.300701	4.7402232	-5.526445	7.26E-05	0.0032625	1.8234801
CD1B	-1.037486	2.6697951	-5.523021	7.31E-05	0.0032625	1.8174506
PPAP2C	-1.599398	5.660006	-5.521985	7.32E-05	0.0032625	1.8156264
LOC1019273	-1.023868	2.8907529	-5.519495	7.35E-05	0.003268	1.8112405
LINC01107	-1.213116	4.4172351	-5.515422	7.41E-05	0.003268	1.804064
AFM	-1.012118	3.4339483	-5.515301	7.41E-05	0.003268	1.8038506

ZNF667-AS1	-1.624608	8.1361629	-5.505026	7.55E-05	0.0033067	1.7857363
NDP	-1.25199	6.1919494	-5.504863	7.55E-05	0.0033067	1.7854487
IRF2BP1	-1.170041	5.0582571	-5.50243	7.58E-05	0.0033071	1.7811562
C6orf203	-1.000417	4.9237104	-5.491916	7.73E-05	0.0033261	1.7626023
TRPM2-AS	-1.072321	5.9578429	-5.491904	7.73E-05	0.0033261	1.7625802
C9orf43	-1.0656	3.5817874	-5.491508	7.73E-05	0.0033261	1.7618819
THEMIS	-2.014818	3.9137251	-5.491396	7.73E-05	0.0033261	1.7616838
JAKMIP1	-1.176	7.1038218	-5.488066	7.78E-05	0.0033367	1.7558032
RAB32	-1.598027	4.4865608	-5.486623	7.80E-05	0.0033384	1.7532546
CEP44	-1.212172	4.2191176	-5.483222	7.85E-05	0.0033518	1.7472468
OIT3	-1.144192	3.1448326	-5.479945	7.89E-05	0.0033647	1.741456
VWCE	-1.082984	5.8534153	-5.478574	7.91E-05	0.003366	1.739033
COL24A1	-1.374218	6.1235416	-5.476502	7.94E-05	0.0033715	1.7353714
STXBP5-AS1	-1.080948	4.3854997	-5.470828	8.02E-05	0.0033991	1.7253379
LYRM1	-1.673769	5.605584	-5.465241	8.11E-05	0.0034158	1.7154551
LOR	-1.213792	3.9514753	-5.463746	8.13E-05	0.0034158	1.7128092
KCNJ1	-1.163433	3.1014284	-5.463533	8.13E-05	0.0034158	1.712433
PCDHB15	-2.631009	4.9850111	-5.459466	8.19E-05	0.0034339	1.7052348
PNPLA4	-1.421868	5.2230106	-5.456231	8.24E-05	0.0034469	1.6995079
LINC01140	-1.189257	4.6974947	-5.45369	8.28E-05	0.0034486	1.6950082
FLJ31356	-1.042723	2.902536	-5.449168	8.34E-05	0.0034591	1.6869984
CTRL	-1.176894	3.9368515	-5.446461	8.38E-05	0.0034591	1.6822014
LOC1019296	-1.141088	3.1403285	-5.446397	8.39E-05	0.0034591	1.6820893
DIS3	-1.016792	4.1874894	-5.444935	8.41E-05	0.0034613	1.6794985
DHFRL1	-1.094229	4.1897936	-5.435279	8.56E-05	0.003508	1.6623783
SPG20OS	-1.133716	3.8960866	-5.430975	8.62E-05	0.0035283	1.6547441
PAQR7	-1.324279	5.8964308	-5.425333	8.71E-05	0.0035537	1.6447329
PIGM	-1.00821	4.2380009	-5.423689	8.74E-05	0.0035537	1.6418135
ROR2	-1.257081	3.2952986	-5.422458	8.76E-05	0.0035537	1.6396281
GDPD1	-1.603718	5.2394517	-5.421499	8.77E-05	0.0035537	1.6379254
VGF	-1.026628	5.9316422	-5.417739	8.83E-05	0.0035709	1.6312496
FGGY	-1.128463	3.7850827	-5.413405	8.90E-05	0.0035849	1.6235503
HIST1H2BJ	-1.09342	3.6779989	-5.405321	9.03E-05	0.0036305	1.6091835
ZFP37	-1.015401	2.911714	-5.397255	9.16E-05	0.0036762	1.5948405
CCDC12	-1.016329	7.2082679	-5.395903	9.19E-05	0.0036762	1.5924354
LOC1019281	1.1387421	3.8464756	5.3951683	9.20E-05	0.0036762	1.5911276
LOC1019304	-1.253961	3.8299287	-5.39005	9.29E-05	0.0036809	1.5820204
XKR4	-1.815186	6.198221	-5.388285	9.32E-05	0.0036809	1.5788785
TRAPPC5	-1.279553	6.5260338	-5.388138	9.32E-05	0.0036809	1.5786162
FAM71F1	-1.059372	4.6872685	-5.386842	9.34E-05	0.0036809	1.5763083
CCDC112	-1.741541	3.8615365	-5.384793	9.37E-05	0.0036809	1.5726613
PRR32	-1.070891	4.315243	-5.383172	9.40E-05	0.0036809	1.5697741
GMNC	-1.151448	3.0354049	-5.382572	9.41E-05	0.0036809	1.5687049
KRTAP7-1	-1.223328	3.1589299	-5.379931	9.46E-05	0.0036832	1.5640013
AQP3	-1.060765	4.7497143	-5.379242	9.47E-05	0.0036832	1.5627743
TENM2	-1.510644	8.0718912	-5.377691	9.50E-05	0.0036837	1.5600119
TNNI3K	-1.134625	2.3874898	-5.374734	9.55E-05	0.0036897	1.5547425
LOC1027236	-1.295301	4.2955465	-5.373939	9.56E-05	0.0036897	1.5533251
TMEM169	-1.860851	4.8742464	-5.373061	9.58E-05	0.0036897	1.5517611
COL15A1	-1.098916	2.2538609	-5.365068	9.72E-05	0.0037295	1.5375117
ERBB3	-1.242012	4.9518589	-5.360381	9.80E-05	0.0037544	1.5291513

LINC00167	-1.063931	2.8463029	-5.353858	9.92E-05	0.003792	1.5175108
KNCN	-1.08852	3.2913381	-5.34862	0.0001001	0.0038073	1.5081597
KBTBD7	-1.168776	3.5068843	-5.348575	0.0001001	0.0038073	1.508079
SCLY	-1.175592	5.5057922	-5.346077	0.0001006	0.0038175	1.5036176
LOC1019298	-1.067225	4.3540979	-5.344756	0.0001008	0.0038196	1.5012581
SLC45A3	-1.110509	3.9229295	-5.339347	0.0001018	0.0038502	1.4915955
C2orf44	-1.147172	4.8744212	-5.336714	0.0001023	0.0038547	1.4868887
PNO1	-1.305704	6.3430782	-5.336595	0.0001023	0.0038547	1.4866777
RRM1	-1.027845	5.8851439	-5.335677	0.0001025	0.0038547	1.4850356
SLC25A46	-1.78714	6.4204362	-5.331864	0.0001032	0.0038744	1.4782189
IL6	1.1162521	4.1756534	5.3292929	0.0001037	0.0038854	1.4736221
ADAM11	-1.179245	6.5608096	-5.325413	0.0001044	0.0038916	1.4666823
TAAR2	-1.556015	3.593641	-5.324264	0.0001046	0.0038927	1.4646274
GGCT	-1.617557	7.3939682	-5.318537	0.0001057	0.0039263	1.4543785
KCNK10	-1.089586	4.9948481	-5.31591	0.0001062	0.003935	1.4496769
ACBD4	-1.126584	5.8839575	-5.307528	0.0001079	0.0039842	1.434666
PRMT5-AS1	-1.343964	4.6709773	-5.306151	0.0001082	0.003987	1.4321995
TMEM119	1.6053942	3.8942298	5.3027144	0.0001088	0.0040049	1.4260427
PCDHB6	-1.402485	4.9026471	-5.301613	0.0001091	0.004005	1.424069
MAZ	-1.076813	6.1969525	-5.291586	0.0001111	0.0040534	1.4060916
THAP11	-1.056906	6.7448729	-5.291215	0.0001111	0.0040534	1.4054261
DHRS4-AS1	-1.078715	5.5968278	-5.284751	0.0001125	0.0040942	1.3938302
ZNF721	-1.072423	7.3628684	-5.282199	0.000113	0.0040977	1.3892508
SLC5A11	-1.075579	6.2140762	-5.279432	0.0001136	0.0040977	1.3842833
PRKAA2	-1.187733	5.5977531	-5.275505	0.0001144	0.0041076	1.3772343
MBD4	-1.35304	6.7370249	-5.275169	0.0001144	0.0041076	1.3766307
LIF	-1.191059	3.4885952	-5.274637	0.0001146	0.0041076	1.3756751
RITA1	-1.194643	4.9954837	-5.271246	0.0001153	0.0041159	1.3695852
AAMP	-1.250289	5.8600513	-5.265459	0.0001165	0.0041453	1.3591876
PTBP2	-1.340794	4.047567	-5.258209	0.0001181	0.0041734	1.3461552
ARL13B	-1.072076	3.5746439	-5.257085	0.0001183	0.0041734	1.3441337
FOXD3-AS1	-1.449534	6.9389834	-5.25681	0.0001184	0.0041734	1.3436399
STAC	-1.238426	3.4712511	-5.256229	0.0001185	0.0041734	1.3425941
GIPC1	-1.186422	7.6610983	-5.256082	0.0001185	0.0041734	1.3423299
LOC375196	-1.078016	4.1008078	-5.252754	0.0001192	0.0041799	1.3363439
LOC101060C	-1.176217	3.8204161	-5.252417	0.0001193	0.0041799	1.3357385
FTH1P5	-1.338807	9.7160715	-5.247268	0.0001204	0.0042123	1.3264739
LOC730098	-1.133969	6.2522539	-5.244183	0.0001211	0.0042189	1.320922
DOCK9-AS2	-1.104401	5.1327077	-5.239007	0.0001223	0.0042331	1.3116035
CSRP3	-1.229367	4.6572377	-5.236093	0.0001229	0.0042477	1.3063565
LOC1002872	-1.3581	3.8886827	-5.235285	0.0001231	0.0042477	1.3049016
ENO2	-1.106056	10.964703	-5.233689	0.0001235	0.004253	1.3020262
LINC00936	-1.280688	7.3726809	-5.229452	0.0001244	0.0042637	1.294393
NPHS2	-1.19049	3.5705037	-5.229275	0.0001245	0.0042637	1.2940743
MYBBP1A	-1.117084	6.5296178	-5.227776	0.0001248	0.0042637	1.2913721
LOC284080	-1.044954	3.1374055	-5.226084	0.0001252	0.0042696	1.2883234
ZKSCAN3	-1.113538	4.2689821	-5.22052	0.0001265	0.0042712	1.2782932
FAM134B	-1.341465	6.3181129	-5.217448	0.0001272	0.0042807	1.2727528
EFCAB1	-1.000887	3.9591555	-5.214104	0.000128	0.0043	1.2667207
LOC1019276	-1.127742	4.1645673	-5.210277	0.0001289	0.0043103	1.2598153
LINC00403	1.3569385	3.6318909	5.2101255	0.0001289	0.0043103	1.2595426

CCPG1	-1.356432	3.7680924	-5.208656	0.0001293	0.0043149	1.2568914
STX18-AS1	-1.194929	2.8696576	-5.206372	0.0001298	0.0043216	1.2527682
GINS3	-1.01681	4.3487093	-5.206037	0.0001299	0.0043216	1.2521629
RAB21	-1.136216	4.0446542	-5.202119	0.0001308	0.0043352	1.2450901
DPF1	-1.175017	6.6707713	-5.201313	0.000131	0.0043352	1.2436344
RWDD3	-1.265806	4.9131286	-5.200801	0.0001312	0.0043352	1.2427097
NRGN	-1.579992	10.25157	-5.195454	0.0001325	0.0043679	1.2330526
PLGLB2	-1.75528	5.9823633	-5.193378	0.000133	0.0043679	1.2293027
C5orf55	-1.111392	4.47388	-5.190567	0.0001336	0.0043821	1.2242222
DGKH	-1.137402	4.9166054	-5.185956	0.0001348	0.0044053	1.2158883
LMO1	-1.153976	4.7483721	-5.184633	0.0001351	0.004409	1.2134963
RNF126P1	-1.264797	3.9672036	-5.182079	0.0001358	0.0044186	1.2088799
SPRYD3	-1.224909	8.1055306	-5.180346	0.0001362	0.0044186	1.2057457
SPRY1	-1.074841	4.6998466	-5.179319	0.0001364	0.0044186	1.2038872
EDRF1	-1.031857	4.1359271	-5.179136	0.0001365	0.0044186	1.2035567
UQCRB	-1.190873	7.3668925	-5.176456	0.0001372	0.0044269	1.1987082
C6orf57	-1.111195	2.4105533	-5.176247	0.0001372	0.0044269	1.1983308
ZNF441	-1.125609	3.0490814	-5.166329	0.0001397	0.0044702	1.1803816
ALLC	-1.670756	4.47057	-5.165935	0.0001398	0.0044702	1.1796693
GLRX2	-1.873253	6.8730573	-5.165284	0.00014	0.0044702	1.1784902
NDUFC1	-1.388374	6.390571	-5.165147	0.0001401	0.0044702	1.1782427
KIR2DS4	-1.509031	3.499561	-5.16361	0.0001404	0.0044759	1.1754591
SFXN1	-1.000496	5.0294669	-5.160962	0.0001411	0.0044794	1.1706648
FAM131B	-1.385268	8.704079	-5.158846	0.0001417	0.0044874	1.1668306
ANAPC15	-1.346357	7.106796	-5.155572	0.0001425	0.0044978	1.1609
KLHL26	-1.284129	6.5967683	-5.150863	0.0001438	0.004519	1.1523664
LINC01267	1.0158035	3.938177	5.1483946	0.0001444	0.0045301	1.1478929
DUSP10	-1.192535	4.9989192	-5.142928	0.0001459	0.0045588	1.137981
LOC1005056	-1.146919	3.2091909	-5.142401	0.000146	0.0045588	1.1370255
ZNF143	-1.571171	4.1764839	-5.141955	0.0001462	0.0045588	1.1362164
DPCR1	-1.099871	3.3972714	-5.138393	0.0001471	0.004575	1.1297557
AREL1	-1.228504	4.9422078	-5.134811	0.0001481	0.0045903	1.1232562
LOC1005068	-1.072303	4.0102967	-5.134116	0.0001483	0.0045903	1.1219953
FBXO40	-1.038751	3.8822921	-5.131403	0.000149	0.0045994	1.1170716
PFKP	-1.028119	6.4229844	-5.128461	0.0001499	0.0046106	1.1117329
TSTD1	-1.636615	5.763411	-5.121746	0.0001517	0.0046372	1.0995383
PHKB	-1.608025	5.4042943	-5.120629	0.000152	0.0046372	1.0975104
GDA	-1.440538	6.3595044	-5.119933	0.0001522	0.0046372	1.0962465
NIP7	-1.153291	4.0140011	-5.117974	0.0001528	0.0046381	1.0926874
LRRN3	-1.478956	7.5064672	-5.117468	0.0001529	0.0046381	1.091768
ZNF334	-1.076146	5.6323685	-5.116217	0.0001533	0.0046381	1.0894957
TRIM58	-1.082896	2.9852882	-5.115983	0.0001534	0.0046381	1.0890693
IMMT	-1.226255	6.5372528	-5.114689	0.0001537	0.0046387	1.0867178
DHRS2	-1.125146	3.7346075	-5.107993	0.0001556	0.0046555	1.0745468
LOC1019295	-1.251123	2.6857941	-5.10797	0.0001556	0.0046555	1.0745066
DHDH	-1.458976	5.3281207	-5.102683	0.0001572	0.0046808	1.0648918
TRPV3	-1.061036	3.9690578	-5.099017	0.0001582	0.0046989	1.0582243
RRAGB	-1.32848	4.4851557	-5.094615	0.0001595	0.0046998	1.0502136
MPND	-1.014237	5.0968121	-5.090429	0.0001608	0.0046998	1.0425956
TNFSF9	-1.147705	6.5508535	-5.087508	0.0001616	0.0047043	1.0372782
EID2	-1.731515	5.3945224	-5.085392	0.0001623	0.0047043	1.0334238

SLC7A13	-1.6532	2.4750817	-5.084487	0.0001626	0.0047043	1.031777
CCDC47	-1.005491	6.4426195	-5.084478	0.0001626	0.0047043	1.0317609
SYP	-1.198979	10.14352	-5.079985	0.0001639	0.0047187	1.0235772
LOC1019281	-1.033548	3.5012114	-5.076622	0.0001649	0.0047331	1.017449
RWDD4	-1.389548	5.3186678	-5.069906	0.000167	0.0047538	1.0052094
LOC1001307	-1.199252	5.920369	-5.06497	0.0001685	0.0047696	0.9962097
FAM50B	-1.109561	8.3179942	-5.062554	0.0001693	0.0047698	0.9918026
ZNF776	-1.944587	3.9391248	-5.055316	0.0001716	0.0048092	0.9785979
C11orf73	-1.351111	7.5853547	-5.05118	0.0001729	0.0048396	0.9710491
ATP5B	-1.329536	9.5454887	-5.049444	0.0001735	0.0048396	0.9678806
NAPG	-1.355052	6.3783544	-5.049115	0.0001736	0.0048396	0.9672792
SNRNP40	-1.291448	6.2657447	-5.048981	0.0001736	0.0048396	0.9670342
ZMAT5	-1.159533	5.062222	-5.047454	0.0001741	0.0048417	0.9642462
CBLN1	-1.146074	3.6518421	-5.046739	0.0001743	0.0048417	0.9629413
HAND2	-1.149221	3.365272	-5.043953	0.0001753	0.004852	0.9578527
MISP	-1.210582	4.3857818	-5.04191	0.0001759	0.0048572	0.9541215
FOLR1	-1.008797	3.063417	-5.038595	0.000177	0.0048659	0.9480653
LOC283682	-1.170225	3.5265303	-5.037964	0.0001772	0.0048659	0.9469137
EIF2AK3	-1.11904	4.1963579	-5.037878	0.0001772	0.0048659	0.9467559
DAW1	-1.18714	3.4735956	-5.036074	0.0001778	0.0048659	0.9434601
LINC00939	-1.029835	4.4919345	-5.035883	0.0001779	0.0048659	0.9431111
SOSTDC1	-1.380957	4.6348223	-5.035181	0.0001781	0.0048659	0.9418271
APLP1	-1.318947	9.3301131	-5.034069	0.0001785	0.0048692	0.9397949
LOC1019281	-1.547879	5.1673805	-5.02991	0.0001799	0.0048942	0.9321928
TRMT112	-1.08951	9.7781044	-5.020414	0.0001831	0.0049682	0.9148278
ZGRF1	-1.209898	3.9144948	-5.015968	0.0001846	0.0049947	0.9066932
KRT37	-1.509337	3.3565532	-5.014832	0.000185	0.0049947	0.904614
LANCL3	-1.724719	3.3438055	-5.014718	0.000185	0.0049947	0.9044064
STEAP1	-1.422126	6.0969402	-5.013251	0.0001855	0.0050017	0.9017213
SCOC-AS1	-1.074176	3.2575481	-5.006042	0.0001881	0.0050483	0.8885237
CYP51A1-AS	-1.091075	2.9154307	-5.003503	0.0001889	0.00506	0.8838734
CD40LG	-1.032898	4.0659408	-4.999984	0.0001902	0.0050773	0.8774275
C2orf80	-2.708987	6.4885133	-4.98755	0.0001947	0.0051388	0.8546394
RPS6KA4	-1.041997	3.6949717	-4.985259	0.0001955	0.0051541	0.8504395
ADAMTS19	-1.408364	5.086033	-4.980115	0.0001974	0.0051935	0.8410038
ASMT	-1.195567	2.7821866	-4.979796	0.0001975	0.0051935	0.8404201
C20orf195	-1.057971	4.4835566	-4.973331	0.0001999	0.0051983	0.8285577
HNRNPAB	-1.183013	7.2952236	-4.97301	0.0002	0.0051983	0.8279686
PANK3	-1.726376	5.0035345	-4.972488	0.0002002	0.0051983	0.8270101
BAI3	-1.370561	5.6950751	-4.97039	0.000201	0.0051983	0.8231594
COX7A2L	-1.271293	8.6942336	-4.969227	0.0002014	0.0051983	0.8210244
RPAP3	-1.127531	3.4634656	-4.969073	0.0002015	0.0051983	0.8207405
PET100	-1.447575	9.6659298	-4.96505	0.000203	0.0052293	0.8133547
LINC00853	-1.007655	3.4797858	-4.962674	0.0002039	0.0052298	0.8089906
LINC00165	-1.303954	4.4067747	-4.954445	0.0002071	0.0052824	0.7938746
PPP1R36	-1.098629	4.2376028	-4.951688	0.0002081	0.005296	0.7888079
EMB	-1.14048	3.5797743	-4.951068	0.0002084	0.005296	0.7876683
ESPNL	-1.126857	5.7165327	-4.948891	0.0002092	0.005311	0.7836667
LAX1	-1.421409	3.1424453	-4.94406	0.0002111	0.0053328	0.774784
AIFM1	-1.392585	7.0629821	-4.939443	0.000213	0.0053601	0.7662927
HTR1E	-1.149542	4.9908821	-4.938973	0.0002131	0.0053601	0.7654265

SLC35A3	-1.016209	3.530037	-4.936404	0.0002142	0.0053634	0.7607006
TMEM194B	-1.53507	6.4804143	-4.933202	0.0002155	0.0053739	0.7548091
LOC283737	-1.378434	3.5921727	-4.932159	0.0002159	0.0053739	0.75289
HINT1	-1.067898	7.6728756	-4.931597	0.0002161	0.0053739	0.751855
TRIM9	-1.539672	6.994711	-4.926323	0.0002182	0.0054125	0.742147
RAB4B	-1.000744	4.1471655	-4.918337	0.0002215	0.0054482	0.72744
NDUFA4	-1.471514	10.54011	-4.913025	0.0002238	0.0054896	0.7176528
SATB2-AS1	-1.021769	4.2325274	-4.912989	0.0002238	0.0054896	0.7175874
SET	-1.596185	8.428527	-4.910665	0.0002247	0.0055004	0.7133044
LOC101927C	-1.217233	5.0447203	-4.909437	0.0002253	0.0055064	0.7110412
LOC1005061	-1.074453	4.7155774	-4.907185	0.0002262	0.0055124	0.7068902
C5orf15	-1.029503	5.0986713	-4.901439	0.0002287	0.0055631	0.6962962
CHCHD1	-1.157429	6.2194675	-4.899425	0.0002295	0.0055709	0.6925804
C1orf94	-1.273688	4.3986257	-4.895187	0.0002314	0.0056032	0.6847638
HSDL1	-1.593514	6.0537068	-4.894553	0.0002316	0.0056032	0.6835944
NHEG1	-1.477979	3.0872792	-4.893194	0.0002322	0.0056032	0.6810859
PRSS3P2	-1.063486	6.6580381	-4.891027	0.0002332	0.0056032	0.677088
FLJ41455	-1.240466	2.7061642	-4.890783	0.0002333	0.0056032	0.6766378
LOC1009962	-1.002626	5.5766634	-4.890667	0.0002333	0.0056032	0.6764225
LOC151657	-1.098381	4.9679267	-4.889451	0.0002339	0.0056032	0.6741785
MED10	-1.19915	6.9779941	-4.888281	0.0002344	0.0056032	0.6720197
LINC00685	-1.138393	3.7996894	-4.888132	0.0002345	0.0056032	0.6717447
KERA	1.2866597	3.7404232	4.8809713	0.0002376	0.0056331	0.6585245
NUDT16L1	-1.20224	6.1902581	-4.879898	0.0002381	0.0056331	0.6565429
PSG1	-1.062348	4.1896139	-4.879383	0.0002383	0.0056331	0.655592
MOGS	-1.324204	5.4476934	-4.879172	0.0002384	0.0056331	0.6552021
ZG16	-1.239819	4.4456401	-4.875355	0.0002401	0.0056334	0.6481508
ALG14	-1.316773	5.2776155	-4.875173	0.0002402	0.0056334	0.6478146
LOC400768	-1.144802	3.6810974	-4.875131	0.0002402	0.0056334	0.6477374
ATP6V0C	-1.065679	7.504916	-4.873354	0.000241	0.0056334	0.6444541
SLC35D3	-1.051089	2.6414695	-4.87052	0.0002423	0.0056514	0.6392187
TAF3	-1.058922	5.0960996	-4.870124	0.0002425	0.0056514	0.6384869
ATP6V1E1	-1.049814	10.096879	-4.867657	0.0002436	0.0056647	0.6339276
SOX21	-1.142601	3.9364183	-4.866568	0.0002441	0.0056699	0.6319154
ABCA3	-1.140894	8.0161532	-4.865921	0.0002444	0.0056704	0.630718
OTUD6B	-1.874089	3.5658849	-4.86457	0.0002451	0.0056735	0.6282221
PARD6A	-1.041281	6.9381811	-4.864422	0.0002451	0.0056735	0.6279472
H2AFY2	-1.396025	6.1738787	-4.861729	0.0002464	0.0056894	0.6229687
DZIP3	-1.346385	6.360879	-4.853988	0.00025	0.0057276	0.6086524
C16orf46	-1.141415	5.4417168	-4.851936	0.000251	0.0057324	0.6048567
OGDH	-1.011048	5.709723	-4.849983	0.0002519	0.0057324	0.6012436
LEMD3	-1.234002	8.0039601	-4.848306	0.0002527	0.0057399	0.5981408
HMP19	-1.309038	8.633537	-4.848111	0.0002528	0.0057399	0.5977787
RAD51-AS1	-1.048275	5.5740574	-4.846304	0.0002536	0.0057531	0.5944345
RRS1	-1.178927	6.9571155	-4.840064	0.0002566	0.0058001	0.5828846
CDKN1C	-1.06663	5.4298777	-4.839518	0.0002569	0.0058001	0.5818734
LOC440570	-1.297647	4.1271837	-4.839044	0.0002571	0.0058001	0.5809958
NAB2	-1.096084	6.8273041	-4.831249	0.0002609	0.0058474	0.5665583
PTH1R	-1.082433	5.6739472	-4.830877	0.0002611	0.0058474	0.5658693
TTPAL	-1.019177	6.3455799	-4.825179	0.0002639	0.0058952	0.5553105
SLC4A2	-1.083501	6.0194327	-4.823273	0.0002649	0.0059099	0.5517778

C14orf2	-1.369945	9.449336	-4.814662	0.0002692	0.0059936	0.5358141
AGA	-1.171821	4.9726037	-4.813193	0.00027	0.0060025	0.5330886
PRMT8	-1.22625	6.1519835	-4.807341	0.000273	0.0060506	0.522233
KRTAP19-3	-1.05691	3.2474571	-4.804802	0.0002743	0.0060731	0.5175213
TSPAN7	-1.342653	9.6800272	-4.801812	0.0002758	0.0060943	0.511973
QPCT	-2.033401	5.1747502	-4.798435	0.0002776	0.0061087	0.5057043
LOC150051	-1.064007	3.4443007	-4.797305	0.0002782	0.0061105	0.5036069
HLTF	-1.420934	6.7624508	-4.796968	0.0002784	0.0061105	0.5029811
TM4SF4	-1.306949	3.2464188	-4.795284	0.0002792	0.0061105	0.4998538
CENPF	-1.110229	3.8725621	-4.790741	0.0002817	0.0061369	0.4914178
EPS8L3	-1.484766	3.8223041	-4.785435	0.0002845	0.0061655	0.4815609
KCNS3	-1.275418	5.4979427	-4.784353	0.0002851	0.0061655	0.4795519
VSIG1	-1.000048	4.1305089	-4.781207	0.0002868	0.0061892	0.4737045
CAAP1	-1.218981	4.7996028	-4.780148	0.0002873	0.0061951	0.4717369
TMEM35	-1.685831	4.0109161	-4.779233	0.0002878	0.0061958	0.4700358
IFI6	-1.456949	8.528519	-4.778416	0.0002883	0.0061958	0.4685177
FLJ41170	-1.115519	5.0125404	-4.774352	0.0002905	0.006224	0.4609628
NRG3	-1.288958	7.5017095	-4.771539	0.0002921	0.0062311	0.4557332
LMBRD2	-1.088694	5.4875068	-4.769835	0.000293	0.0062447	0.452563
PHAX	-1.3341	5.8376332	-4.766189	0.000295	0.0062483	0.4457819
LOC1019285	-1.011319	3.1487079	-4.765718	0.0002953	0.0062483	0.4449055
WDR54	-1.195641	6.9287573	-4.765625	0.0002953	0.0062483	0.4447341
FDFT1	-1.004439	7.5347431	-4.765609	0.0002954	0.0062483	0.4447037
FARSB	-1.14451	4.9841753	-4.764871	0.0002958	0.0062483	0.44333
NDRG1	-1.404876	8.6926195	-4.760824	0.000298	0.0062801	0.4358007
ENY2	-1.042441	6.4007409	-4.759021	0.0002991	0.0062951	0.4324452
CEL	-1.050581	2.5444058	-4.756421	0.0003005	0.0063197	0.4276064
SVIP	-1.242709	5.8653014	-4.753493	0.0003022	0.0063463	0.4221568
WDYHV1	-1.30288	3.6613012	-4.752312	0.0003029	0.0063463	0.4199581
CTSA	-1.115621	8.1116693	-4.748276	0.0003052	0.0063591	0.4124436
FLJ34503	-1.391591	2.8462784	-4.741561	0.0003091	0.0064079	0.3999358
DGKB	-1.050864	5.9921192	-4.72937	0.0003163	0.0065057	0.3772156
SLMO1	-1.121953	4.0352161	-4.724016	0.0003196	0.0065295	0.3672324
SPTY2D1	-1.168322	3.3673988	-4.719702	0.0003222	0.0065641	0.3591872
CYP4X1	-1.37022	6.6217051	-4.713745	0.0003259	0.0065893	0.3480736
KLHL3	-1.074099	7.4148228	-4.711752	0.0003271	0.0066001	0.3443531
LOC283856	-1.074705	3.5355943	-4.709495	0.0003285	0.0066219	0.3401403
LOC101928C	-1.422991	4.0616477	-4.707758	0.0003296	0.0066226	0.3368982
MRPL24	-1.052844	6.9373218	-4.707309	0.0003299	0.0066226	0.3360606
KIFC2	-1.037975	8.1861019	-4.706893	0.0003301	0.0066226	0.3352845
WDR5	-1.015082	5.9893687	-4.701221	0.0003337	0.0066555	0.3246923
DUSP21	-1.146168	3.3875745	-4.699944	0.0003345	0.0066625	0.3223091
LOC440416	-1.076156	3.6045311	-4.689249	0.0003414	0.0067522	0.3023287
NME9	-1.506836	3.9740472	-4.685965	0.0003435	0.0067778	0.2961911
CYP8B1	-1.031246	3.8381533	-4.675864	0.0003502	0.0068771	0.2773038
SLC2A3	-1.274939	6.9424394	-4.675288	0.0003506	0.0068771	0.2762272
COL21A1	-2.223061	3.9762236	-4.667251	0.000356	0.0069626	0.2611911
TUBBP5	-1.084092	3.6860193	-4.666791	0.0003563	0.0069626	0.2603311
BRCC3	-1.06714	5.4605432	-4.663389	0.0003586	0.0069745	0.2539644
LOC441081	-1.751958	5.9909735	-4.660371	0.0003607	0.0069954	0.2483159
IL1F10	-1.007622	3.5062813	-4.659899	0.000361	0.0069954	0.2474309

PEBP4	-1.334923	4.6418164	-4.659825	0.0003611	0.0069954	0.2472934
IMP3	-1.069391	6.947743	-4.65462	0.0003647	0.0070451	0.2375485
KBTBD8	-1.034253	3.5370494	-4.65282	0.0003659	0.0070546	0.2341778
YWHAZ	-1.360234	8.364756	-4.652424	0.0003662	0.0070546	0.2334352
TTI1	-1.728486	5.002529	-4.651905	0.0003665	0.007055	0.232463
AZIN2	-1.305076	7.6034938	-4.650759	0.0003673	0.0070628	0.2303165
TERF1	-1.136591	7.0306475	-4.650337	0.0003676	0.0070628	0.2295267
DDX55	-1.375667	4.7228757	-4.648507	0.0003689	0.0070742	0.2260991
SP2	-1.099917	4.0459459	-4.646898	0.0003701	0.007076	0.2230835
LOC1005068	-1.020293	3.1588401	-4.640406	0.0003747	0.0071375	0.2109207
LRRK2	-1.070348	4.6083405	-4.639392	0.0003754	0.0071434	0.2090187
SMIM6	-1.371626	4.3202601	-4.638793	0.0003758	0.0071434	0.2078974
MAB21L2	-1.100232	4.3266595	-4.633676	0.0003795	0.0071805	0.1983047
MCTP1	-1.7049	5.1406239	-4.633391	0.0003797	0.0071805	0.1977714
CCDC81	-1.241495	4.5443538	-4.632274	0.0003805	0.0071805	0.1956756
STX3	-1.367457	5.6027503	-4.631028	0.0003814	0.0071805	0.1933405
SENP6	-1.057298	4.754706	-4.630666	0.0003817	0.0071805	0.192662
SH2D1B	-1.174156	4.0765383	-4.630097	0.0003821	0.0071805	0.1915948
PAXIP1	-1.456485	4.8477911	-4.629984	0.0003822	0.0071805	0.1913825
TBC1D25	-1.163062	5.5517409	-4.628799	0.0003831	0.0071902	0.1891597
MPDZ	-1.032588	7.7562605	-4.624769	0.000386	0.0072288	0.1816024
EXTL1	-1.138561	6.0375657	-4.623079	0.0003873	0.0072342	0.1784317
MIS12	-1.305419	4.4093655	-4.622311	0.0003878	0.0072342	0.1769907
C3orf62	-1.061382	3.2554428	-4.621843	0.0003882	0.0072342	0.1761126
SLC25A43	-1.063071	2.4841504	-4.621774	0.0003882	0.0072342	0.1759825
GNB4	-1.004298	5.0614605	-4.620571	0.0003891	0.0072342	0.1737268
NFYB	-1.042342	4.2315272	-4.615374	0.000393	0.0072814	0.1639736
AHNAK2	-1.103421	6.8136287	-4.615038	0.0003933	0.0072814	0.1633433
DUSP13	-1.148011	3.5187469	-4.613464	0.0003944	0.0072835	0.1603896
SOX5	-1.058932	6.0703605	-4.608331	0.0003983	0.0073216	0.1507533
RAP2C	-1.210929	4.6660818	-4.607909	0.0003986	0.0073216	0.1499616
LOC101928C	-1.389558	3.8063115	-4.606223	0.0003999	0.0073223	0.1467961
PCDHAC1	-1.265442	4.6394035	-4.598004	0.0004063	0.0073951	0.1313592
RNF138	-1.504157	5.0398165	-4.597301	0.0004068	0.0073984	0.1300388
BABAM1	-1.286904	7.3647288	-4.595696	0.0004081	0.0074146	0.1270225
GBGT1	-1.198618	4.8494075	-4.58939	0.000413	0.0074516	0.1151726
SLC50A1	-1.033442	5.9551315	-4.587746	0.0004143	0.0074586	0.1120825
LINC01069	-1.128036	3.3347557	-4.585139	0.0004164	0.0074586	0.1071812
GRAMD1C	-1.102424	3.3153696	-4.584682	0.0004167	0.0074586	0.106322
PAPSS1	-1.020751	8.0026136	-4.584173	0.0004172	0.0074586	0.1053656
DEPDC7	-1.224899	2.3795667	-4.581658	0.0004192	0.0074586	0.1006368
MAP1B	-1.010545	10.427563	-4.581578	0.0004192	0.0074586	0.1004865
PRPF19	-1.147569	7.6250696	-4.580091	0.0004204	0.0074668	0.0976912
NXNL1	-1.033459	5.7332917	-4.57924	0.0004211	0.0074725	0.0960906
LOC100507C	-1.252455	4.5045458	-4.578227	0.0004219	0.0074805	0.0941854
C17orf100	-1.415834	4.8287031	-4.571074	0.0004277	0.007538	0.0807297
ZFAT	-1.158771	5.3972799	-4.570596	0.0004281	0.0075384	0.0798304
GPR155	-1.300498	5.7847414	-4.569119	0.0004293	0.0075467	0.0770515
CTC-338M12	-1.131082	3.9453068	-4.566717	0.0004313	0.0075594	0.0725332
PVRIG	-1.053385	4.0497004	-4.565008	0.0004327	0.0075594	0.0693158
HCN3	-1.134848	5.8683131	-4.564384	0.0004333	0.0075594	0.0681411

ARRDC3-AS1	-1.14758	4.233391	-4.560849	0.0004362	0.0075792	0.0614878
SPAG16	-1.021547	3.8939631	-4.558102	0.0004385	0.0076052	0.0563159
CYP27C1	-1.007116	2.5146348	-4.557963	0.0004386	0.0076052	0.0560556
DUSP2	-1.205442	5.5042131	-4.556154	0.0004401	0.0076252	0.0526483
C15orf32	-1.010594	3.4751768	-4.553054	0.0004428	0.0076576	0.0468118
TUSC8	-1.239681	3.728501	-4.551267	0.0004443	0.0076709	0.0434468
LINC00208	-1.020739	4.3152246	-4.549375	0.0004459	0.007685	0.039882
KBTBD12	-1.31596	4.7582183	-4.548992	0.0004462	0.007685	0.0391608
PRKCB	-1.086413	8.3017597	-4.545643	0.0004491	0.0077075	0.0328524
ZFP1	-1.012658	4.7843815	-4.540513	0.0004535	0.0077495	0.0231864
HMCES	-1.002793	5.7213305	-4.539985	0.000454	0.0077495	0.0221904
PEX12	-1.106992	3.7403195	-4.537506	0.0004562	0.0077528	0.0175182
LOC1005071	-1.378987	3.4819466	-4.537407	0.0004563	0.0077528	0.0173318
COMMD1	-1.008796	7.9772875	-4.537084	0.0004565	0.0077528	0.0167231
CCM2	-1.260013	6.4890096	-4.535756	0.0004577	0.0077528	0.0142198
WDR66	-1.081698	4.5042215	-4.533649	0.0004596	0.0077706	0.0102494
LOC1027253	-1.069059	4.7393398	-4.521648	0.0004703	0.0078999	-0.012383
SRD5A3	-1.14031	4.8807178	-4.517457	0.0004741	0.0079372	-0.020289
LDLRAD4	-1.051735	6.2468059	-4.515471	0.0004759	0.0079454	-0.024036
PPL	-1.300026	6.805219	-4.511872	0.0004792	0.0079837	-0.030828
ITPK1-AS1	-1.039866	3.8739774	-4.507757	0.000483	0.0080183	-0.038596
LARGE	-1.390675	6.1132961	-4.507504	0.0004832	0.0080183	-0.039072
HS3ST6	-1.043661	4.5559242	-4.504351	0.0004862	0.0080516	-0.045026
ALOX12B	-1.437272	3.8284585	-4.5006	0.0004897	0.0080796	-0.05211
USP27X-AS1	-1.194518	3.7959307	-4.498986	0.0004912	0.0080916	-0.055157
ANKRD20A5F	-1.168863	3.6640985	-4.494316	0.0004956	0.0081243	-0.063978
CASQ1	-1.52737	4.0153615	-4.494078	0.0004958	0.0081243	-0.064427
PHF11	-1.074071	4.3285478	-4.493552	0.0004964	0.0081243	-0.065422
KLHDC1	-1.06678	3.0236321	-4.489383	0.0005003	0.0081687	-0.073298
CAPNS2	-1.086925	3.2075531	-4.487292	0.0005024	0.0081769	-0.077249
TOLLIP-AS1	-1.196387	4.2200621	-4.48548	0.0005041	0.0081924	-0.080673
LSM4	-1.028775	7.5648773	-4.484553	0.000505	0.0082005	-0.082426
ARL2	-1.113329	7.5274235	-4.481123	0.0005084	0.0082418	-0.088908
C4orf29	-1.003494	4.4019201	-4.478372	0.0005111	0.0082559	-0.094109
ARMC10	-1.267166	6.9018619	-4.477766	0.0005117	0.0082559	-0.095254
ZNF512	-1.164607	7.0553653	-4.475413	0.000514	0.008278	-0.099703
LPAR4	1.2529139	3.8892088	4.4749686	0.0005144	0.008278	-0.100544
SFMBT1	-1.080341	4.9103224	-4.47429	0.0005151	0.008278	-0.101827
KCTD18	-1.045794	5.0305759	-4.469216	0.0005202	0.0083078	-0.111423
LINC00343	-1.057815	2.4615439	-4.467338	0.000522	0.0083314	-0.114976
HRAS	-1.062286	6.8448158	-4.466476	0.0005229	0.0083362	-0.116605
LOC1019292	-1.358101	3.4530963	-4.460991	0.0005285	0.0083881	-0.126984
NDUFAF2	-1.289975	7.1574905	-4.458405	0.0005311	0.0084098	-0.131878
FER1L6-AS1	1.4068634	3.5712728	4.4538689	0.0005358	0.0084469	-0.140463
MYRIP	-1.175531	5.3563642	-4.447706	0.0005422	0.0085328	-0.15213
BOD1	-1.261483	6.8310684	-4.444825	0.0005452	0.0085475	-0.157586
MMP2	-1.066937	4.0630004	-4.441109	0.0005491	0.0085826	-0.164623
LOC1005065	-1.334809	5.4830818	-4.438088	0.0005523	0.0086197	-0.170347
NDUFS6	-1.012766	9.7048551	-4.429988	0.000561	0.0087222	-0.185694
LOC1005075	-1.133976	4.0212337	-4.42698	0.0005643	0.0087663	-0.191395
MDH1B	-1.347831	4.6950948	-4.424323	0.0005672	0.0087821	-0.196433

SERPINA3	2.755891	6.0762453	4.4240987	0.0005674	0.0087821	-0.196857
SELT	-1.021903	8.7771887	-4.423921	0.0005676	0.0087821	-0.197193
SFR1	-1.128779	3.7350883	-4.423672	0.0005679	0.0087821	-0.197665
TIMM10B	-1.00365	7.2643238	-4.423054	0.0005686	0.0087821	-0.198836
SCCPDH	-1.112313	7.5638998	-4.421336	0.0005705	0.0087955	-0.202094
LRRC46	-1.012232	3.6802183	-4.420169	0.0005718	0.0088017	-0.204307
INSM2	-1.163737	5.8251433	-4.419207	0.0005728	0.0088052	-0.206131
LINC00309	-1.740774	3.4884805	-4.41864	0.0005734	0.0088083	-0.207206
LOC1019269	-1.051209	2.8478783	-4.414971	0.0005775	0.0088429	-0.214164
SGPP2	-1.578305	6.0690498	-4.41467	0.0005779	0.0088429	-0.214735
TMEM115	-1.145148	6.5020804	-4.408173	0.0005852	0.0089311	-0.227059
OCR1	-1.457573	4.6760355	-4.407983	0.0005854	0.0089311	-0.227418
B3GNT4	-1.115826	4.8206618	-4.405928	0.0005877	0.0089533	-0.231319
CCDC91	-1.273898	4.06069	-4.404786	0.000589	0.0089664	-0.233484
KRT17P5	-1.105091	3.2905233	-4.40289	0.0005912	0.0089793	-0.237083
PLA2G7	1.3277622	5.7078277	4.4006175	0.0005938	0.0090054	-0.241395
RNF166	-1.318279	4.6410696	-4.397913	0.0005969	0.0090325	-0.246527
ZNF808	-1.437587	7.2351572	-4.396936	0.000598	0.0090429	-0.248382
FAM151A	-1.104692	3.4827291	-4.395497	0.0005997	0.0090548	-0.251115
ISOC1	-1.495282	4.3866738	-4.377049	0.0006215	0.0092785	-0.286149
ZNF639	-1.027912	4.6263103	-4.376536	0.0006221	0.0092785	-0.287123
DLEU1	-1.019024	4.0698202	-4.376327	0.0006223	0.0092785	-0.287519
LINC00330	-1.010493	3.1142802	-4.371107	0.0006286	0.0093271	-0.29744
TMEM235	-1.476177	4.9249896	-4.370536	0.0006293	0.0093306	-0.298524
LPP-AS2	-1.003482	2.5221642	-4.368194	0.0006322	0.0093383	-0.302975
HPCA	-1.099787	7.5782522	-4.367872	0.0006326	0.0093383	-0.303587
DPEP1	-1.082633	5.8005146	-4.3616	0.0006403	0.0094283	-0.31551
PRKAB2	-1.04269	5.6950131	-4.360644	0.0006415	0.0094352	-0.317328
LOC1005058	-1.013663	4.1371316	-4.360081	0.0006422	0.0094352	-0.318399
CNIH2	-1.106218	5.0725265	-4.359939	0.0006424	0.0094352	-0.318669
OLFM2	-1.223316	6.8614103	-4.353089	0.000651	0.0095043	-0.331695
GALNT11	-1.079551	8.9634068	-4.346964	0.0006587	0.0095723	-0.343346
NTNG2	-1.341087	8.4235578	-4.344206	0.0006623	0.0095761	-0.348595
PGRMC1	-1.061014	7.4828185	-4.344205	0.0006623	0.0095761	-0.348597
PWP2	-1.069369	5.4338096	-4.342775	0.0006641	0.0095823	-0.351318
DCTN3	-1.156287	8.2201103	-4.340683	0.0006668	0.0096145	-0.355298
RPRML	-1.344084	7.5544668	-4.340005	0.0006677	0.0096204	-0.356588
JAKMIP2-AS	-1.043183	3.3598352	-4.33938	0.0006685	0.0096253	-0.357779
RAD54B	-1.009194	4.8775095	-4.338326	0.0006699	0.0096382	-0.359784
DCSTAMP	-1.045067	4.7255872	-4.337844	0.0006705	0.0096404	-0.360702
MANSC1	-1.338842	6.7291302	-4.334443	0.0006749	0.0096726	-0.367175
PGAM1	-1.152091	9.8360414	-4.333988	0.0006755	0.0096726	-0.368041
GRIN3A	-1.147099	5.2260086	-4.330539	0.0006801	0.0096968	-0.374608
LOC1005075	-1.491463	2.8018486	-4.330022	0.0006807	0.0096992	-0.375592
ZC2HC1B	-1.248179	4.0638692	-4.328876	0.0006823	0.0097073	-0.377774
RPP30	-1.031978	4.554689	-4.328417	0.0006829	0.0097092	-0.378648
IGLL3P	-1.063934	6.0547416	-4.326476	0.0006854	0.009724	-0.382344
ILF2	-1.562423	5.458117	-4.324007	0.0006887	0.009746	-0.387046
RSPH9	-1.026469	3.9379451	-4.323419	0.0006895	0.0097496	-0.388166
TTC33	-1.089155	5.9518006	-4.320542	0.0006934	0.0097577	-0.393646
TCHH	-1.267128	3.5219771	-4.320496	0.0006934	0.0097577	-0.393733

RPGR	-2.064258	6.5318152	-4.31934	0.000695	0.0097577	-0.395935
SOHLH2	-1.550341	3.3477932	-4.318823	0.0006957	0.0097577	-0.39692
MRGPRX4	-1.153625	3.9144647	-4.318237	0.0006965	0.0097577	-0.398037
ANKRD33B	-1.16131	7.5719025	-4.316398	0.000699	0.0097577	-0.401539
LSM7	-1.307575	6.8203548	-4.316007	0.0006995	0.0097577	-0.402284
HS3ST4	-1.011002	8.3293676	-4.315469	0.0007002	0.0097577	-0.40331
PRO2949	-1.093719	3.0679757	-4.315277	0.0007005	0.0097577	-0.403674
ITGAE	-1.441191	6.3421772	-4.315094	0.0007008	0.0097577	-0.404023
TREML5P	-1.283308	4.1317742	-4.314864	0.0007011	0.0097577	-0.404463
AZI2	-1.043951	4.9953626	-4.312397	0.0007044	0.0097846	-0.409162
NUP43	-1.048142	5.2677249	-4.306503	0.0007125	0.0098438	-0.420394
CCNT2-AS1	-1.22849	4.2188415	-4.305284	0.0007142	0.0098538	-0.422717
LINC00957	-1.149028	6.0215182	-4.303747	0.0007164	0.0098766	-0.425647
RNF165	-1.100681	5.6610767	-4.297592	0.000725	0.0099552	-0.437381
LINC00710	-1.179971	3.0193292	-4.296757	0.0007262	0.0099622	-0.438973
FLG2	-1.806368	4.7042202	-4.296007	0.0007272	0.0099659	-0.440403
LOC1001349	-1.196296	5.8112681	-4.294787	0.000729	0.0099776	-0.44273
CYP1A1	-1.089922	4.2368425	-4.284594	0.0007435	0.0101419	-0.46217
PNMA2	-1.025522	8.5720586	-4.283348	0.0007453	0.0101462	-0.464547
PRO0471	-1.128196	2.8214222	-4.281876	0.0007475	0.0101685	-0.467357
COL22A1	-1.591643	3.6092458	-4.278214	0.0007528	0.010214	-0.474344
CRISPLD1	1.2032614	3.8282141	4.276999	0.0007546	0.0102227	-0.476662
LOC1005065	-1.070954	4.8919004	-4.276733	0.000755	0.0102227	-0.477169
CHMP2B	-1.186158	5.417817	-4.273994	0.000759	0.0102439	-0.482397
MRPL34	-1.108461	6.1834465	-4.271485	0.0007627	0.0102601	-0.487184
OBP2B	-1.305567	3.9557502	-4.268197	0.0007676	0.0103057	-0.493462
ERVK13-1	-1.064751	4.8963941	-4.2671	0.0007693	0.010321	-0.495556
DPH6-AS1	-1.231576	2.7224832	-4.265506	0.0007717	0.0103225	-0.498599
BBS5	-1.173322	3.7628063	-4.264929	0.0007725	0.0103225	-0.4997
NDUFA5	-1.099705	4.6694233	-4.264851	0.0007727	0.0103225	-0.49985
TMPRSS4	-1.077281	4.4276899	-4.26431	0.0007735	0.0103225	-0.500882
ZNF542P	-1.200621	4.1146861	-4.263436	0.0007748	0.0103225	-0.50255
PDLIM2	-1.014333	5.4404853	-4.263332	0.0007749	0.0103225	-0.50275
LOC283454	-2.309111	6.1249125	-4.261826	0.0007772	0.0103314	-0.505626
CPNE1	-1.465138	7.9523217	-4.256891	0.0007847	0.010403	-0.515051
GTF2IRD2B	-1.135526	4.6475602	-4.256672	0.0007851	0.010403	-0.515469
NOM1	-1.074514	4.2625668	-4.256322	0.0007856	0.0104033	-0.516136
TAS2R38	-1.185833	3.7067023	-4.254115	0.000789	0.0104346	-0.520351
VARS	-1.006122	6.9873821	-4.252793	0.000791	0.010448	-0.522877
PRPS2	-1.745984	6.154411	-4.252126	0.000792	0.0104481	-0.524152
MRPL2	-1.204983	5.3133384	-4.246196	0.0008012	0.0105491	-0.53548
TRPA1	-1.017611	4.5809309	-4.244118	0.0008045	0.0105688	-0.539451
CRBN	-1.037164	7.7805934	-4.243541	0.0008054	0.0105688	-0.540554
SRSF11	-1.679252	5.3905239	-4.243405	0.0008056	0.0105688	-0.540813
ZNF701	-1.069527	4.339838	-4.241753	0.0008082	0.0105688	-0.54397
SLC5A4	-1.242371	4.241875	-4.239617	0.0008116	0.0105788	-0.548053
DOC2A	-1.638745	8.8254818	-4.239497	0.0008118	0.0105788	-0.548282
SORCS3-AS1	-1.414497	5.2844434	-4.239496	0.0008118	0.0105788	-0.548283
CCM2L	-1.001703	3.550818	-4.236646	0.0008163	0.0106107	-0.55373
GABRA5	-1.668528	6.8459654	-4.235678	0.0008178	0.0106164	-0.555582
PRKCI	-1.162462	7.7829561	-4.235615	0.0008179	0.0106164	-0.555701

BTNL9	-1.079211	4.7716124	-4.235395	0.0008183	0.0106164	-0.556122
LOC1009964	-1.148112	5.5567701	-4.235057	0.0008188	0.0106166	-0.556768
ART3	-1.437293	5.05266	-4.234282	0.0008201	0.0106259	-0.558249
GRM4	-1.004238	5.696563	-4.232967	0.0008222	0.01063	-0.560763
KLHDC3	-1.109142	8.8121576	-4.231649	0.0008243	0.01063	-0.563283
DISP2	-1.221657	6.4566787	-4.229641	0.0008275	0.0106416	-0.567122
KIR2DS1	-1.016879	4.3069158	-4.223479	0.0008375	0.0107097	-0.578904
ZNF284	-1.480132	4.6068887	-4.218976	0.0008449	0.0107571	-0.587518
YPEL2	-1.168428	6.8508288	-4.216138	0.0008496	0.0107766	-0.592945
GRM2	-1.211015	6.5244168	-4.21224	0.0008561	0.0108253	-0.600404
KCNK1	-1.052786	6.3443378	-4.209491	0.0008607	0.0108679	-0.605663
CFD	-1.10211	4.2042113	-4.209278	0.000861	0.0108679	-0.60607
LINC01339	-1.024374	4.5448617	-4.208513	0.0008623	0.010876	-0.607534
ZNF541	-1.230308	3.9408937	-4.206118	0.0008664	0.0108917	-0.612118
NDUFS3	-1.029899	8.2827447	-4.20518	0.000868	0.0108917	-0.613912
CDC7	-1.479632	5.0107673	-4.204042	0.0008699	0.0108917	-0.61609
ALMS1P	-1.039784	5.7590129	-4.202263	0.0008729	0.0108939	-0.619496
OSGEP	-1.125495	6.1331102	-4.201081	0.0008749	0.0109086	-0.621758
BVES-AS1	-1.132461	3.815268	-4.197583	0.0008809	0.0109591	-0.628453
BC048103 /	-1.012231	5.4737097	-4.197466	0.0008811	0.0109591	-0.628677
H2BFM	-1.036634	3.3978552	-4.196901	0.0008821	0.0109646	-0.629759
FAM86C1	-1.135172	5.8244133	-4.193928	0.0008872	0.0110017	-0.635451
ASPRV1	-1.149433	5.3377411	-4.1926	0.0008895	0.0110121	-0.637994
USP30	-1.021944	5.6708689	-4.192514	0.0008897	0.0110121	-0.638157
MAGI2-AS2	1.1423964	3.4765293	4.1869169	0.0008995	0.0110795	-0.648876
SH2D5	-1.131236	6.9815086	-4.185659	0.0009017	0.011087	-0.651285
PRKXP1	-1.081474	4.9082026	-4.183704	0.0009051	0.0111143	-0.65503
KCNE1	-1.110702	2.9769158	-4.183386	0.0009057	0.0111143	-0.655638
CAPNS1	-1.196642	8.7045717	-4.181816	0.0009085	0.0111189	-0.658645
LINC00845	-1.048956	5.4512283	-4.178651	0.0009141	0.0111458	-0.664708
CH25H	-1.50909	4.0476208	-4.174737	0.0009211	0.0112047	-0.672205
HIP1	-1.032531	4.0087394	-4.173295	0.0009237	0.0112297	-0.674969
ETV3	-1.108742	6.267431	-4.172445	0.0009253	0.011235	-0.676597
LYRM9	-1.221289	5.3313388	-4.17205	0.000926	0.0112367	-0.677356
PPEF1	-2.215378	5.1373206	-4.17176	0.0009265	0.0112367	-0.67791
RFPL1	-1.051246	2.3971375	-4.164728	0.0009393	0.0113089	-0.691387
SLC38A9	-1.17502	4.8918705	-4.164724	0.0009393	0.0113089	-0.691393
FCHSD2	-1.273026	5.4036662	-4.16259	0.0009433	0.0113295	-0.695485
C1orf226	-1.046936	5.4062652	-4.159729	0.0009486	0.0113418	-0.700968
LOC1019281	-1.213777	3.5243864	-4.15798	0.0009518	0.011366	-0.704321
FAM103A1	-1.272631	6.7171801	-4.157493	0.0009527	0.011366	-0.705255
TRIM78P	-1.034533	3.143512	-4.156508	0.0009546	0.0113746	-0.707143
RRH	-1.021486	3.6732467	-4.155847	0.0009558	0.0113761	-0.70841
SLC25A26	-1.076597	7.1832709	-4.15538	0.0009567	0.0113799	-0.709306
LY86-AS1	-1.481141	4.5751345	-4.154767	0.0009578	0.0113803	-0.71048
C3orf36	-1.032775	3.733268	-4.153602	0.00096	0.0113996	-0.712715
ZNF468	-1.061692	3.4562933	-4.149137	0.0009684	0.0114797	-0.721276
MACROD2-AS	-1.145728	2.7951233	-4.143115	0.0009799	0.011528	-0.732827
INTS4L1	-1.126492	4.5078072	-4.143061	0.00098	0.011528	-0.73293
EGF	-1.139056	3.3602323	-4.142707	0.0009807	0.011528	-0.733609
PSMD7	-1.402743	6.7208046	-4.135666	0.0009943	0.0116177	-0.747116

KIF13B	-1.03516	7.4692638	-4.130369	0.0010047	0.0116859	-0.757279
LPPR2	-1.054409	7.6271901	-4.129718	0.0010059	0.0116865	-0.758528
FUT11	-1.173033	4.439625	-4.128891	0.0010076	0.0116865	-0.760116
HAR1A	-1.140543	6.6449387	-4.126981	0.0010114	0.0117058	-0.763782
SLCO1A2	-1.500743	3.7401347	-4.126606	0.0010121	0.0117058	-0.7645
LHPP	-1.010335	4.6407242	-4.125608	0.0010141	0.0117135	-0.766416
LOC1005057	-1.950972	5.4592326	-4.123464	0.0010183	0.011721	-0.770531
IGSF9	-1.097104	5.3491035	-4.123136	0.001019	0.011721	-0.771161
WFIKKN1	-1.026184	5.1359364	-4.12027	0.0010247	0.0117392	-0.776661
PKNOX1	-1.016846	6.2318795	-4.118625	0.001028	0.01175	-0.779819
APCDD1L	-1.028535	4.5205193	-4.118092	0.0010291	0.01175	-0.780842
SETBP1	-1.299709	6.3048577	-4.117052	0.0010312	0.01175	-0.782838
CYP26A1	-1.090108	3.8272645	-4.116783	0.0010318	0.01175	-0.783355
SCP2D1	-1.062037	3.4918935	-4.115326	0.0010347	0.01175	-0.786152
MED11	-1.113238	5.9201955	-4.114246	0.0010369	0.01175	-0.788225
XPA	-1.067268	6.6934712	-4.110426	0.0010447	0.0118045	-0.79556
RASD2	-1.682415	6.9641379	-4.108189	0.0010493	0.011848	-0.799857
LOC1019283	-1.213167	3.9123839	-4.106087	0.0010536	0.0118831	-0.803894
TMEM56	-1.50088	5.8435426	-4.105664	0.0010545	0.0118831	-0.804705
NXPH4	-1.235883	3.8237345	-4.101152	0.0010639	0.0119598	-0.813372
B3GALNT1	-1.237101	4.7293277	-4.098011	0.0010704	0.0120073	-0.819406
PRR26	1.0231946	4.5012882	4.0930956	0.0010808	0.0120886	-0.828848
LOC1027237	-1.060885	3.2472711	-4.092624	0.0010818	0.0120886	-0.829755
LOC101928C	-1.055716	3.4362108	-4.09059	0.0010861	0.0121235	-0.833662
ITPR1-AS1	-1.475388	4.5767201	-4.08908	0.0010894	0.0121452	-0.836563
TUBB4B	-1.264385	9.6442807	-4.08621	0.0010955	0.0121684	-0.842079
FAM221B	-1.223561	3.7881017	-4.083657	0.001101	0.0122091	-0.846984
BC069782 /	-1.163618	5.2192105	-4.082997	0.0011024	0.0122122	-0.848252
AKT1S1	-1.114222	3.4653779	-4.079578	0.0011099	0.0122402	-0.854825
GPC4	-1.029441	4.8691456	-4.077504	0.0011144	0.0122618	-0.85881
USP46	-1.121696	5.3908408	-4.077359	0.0011147	0.0122618	-0.859089
NDUFAB1	-1.365049	6.704615	-4.07506	0.0011197	0.0122804	-0.863509
CHIAP2	-1.128409	3.3050372	-4.074836	0.0011202	0.0122804	-0.863939
MOCS2	-1.187896	6.1225093	-4.073152	0.001124	0.0122908	-0.867178
LRRC4C	-1.010297	8.2842622	-4.072808	0.0011247	0.0122925	-0.867838
R3HCC1L	-1.039195	4.5848232	-4.07161	0.0011274	0.0123017	-0.870141
LYSMD3	-1.554933	4.7523178	-4.069103	0.0011329	0.0123427	-0.874962
OTUD6B-AS1	-1.132869	4.3311208	-4.066435	0.0011389	0.0123877	-0.880091
SNCG	-1.109377	6.8048832	-4.065908	0.0011401	0.012394	-0.881105
STK32C	-1.172235	6.4862773	-4.065431	0.0011411	0.012399	-0.882022
ZNF613	-1.04911	3.0493387	-4.063971	0.0011444	0.0124253	-0.88483
KCNJ14	-1.111036	4.3329545	-4.063483	0.0011455	0.0124267	-0.885769
ZC3H13	-1.268449	5.873797	-4.056891	0.0011604	0.0125289	-0.898445
PPM1B	-1.562771	6.5924747	-4.05435	0.0011662	0.0125651	-0.903334
OVGP1	-1.12386	5.1227785	-4.052159	0.0011713	0.0125994	-0.907548
PLAA	-1.268391	4.4020287	-4.049422	0.0011776	0.0126474	-0.912813
ZCCHC12	-1.01927	5.7579132	-4.045931	0.0011857	0.0127011	-0.91953
AFAP1L2	-1.298142	6.2702599	-4.042871	0.0011928	0.0127292	-0.925418
SPATA1	-1.22082	4.513798	-4.042552	0.0011936	0.0127292	-0.926031
POLR1D	-1.158151	5.538581	-4.042439	0.0011938	0.0127292	-0.926249
EPM2A	-1.08064	6.4770952	-4.042361	0.001194	0.0127292	-0.926399

LIN28A	-1.175867	4.621688	-4.040853	0.0011976	0.012747	-0.929302
PCDH19	-1.096859	5.6126851	-4.04021	0.0011991	0.012747	-0.930539
OR7E104P	-1.022992	5.8947255	-4.039922	0.0011998	0.012747	-0.931092
TOP1	-1.157434	6.8786748	-4.035739	0.0012097	0.0128106	-0.939142
PARK7	-1.133745	9.2476487	-4.030522	0.0012222	0.0129092	-0.949183
LOC729680	-1.227401	6.9153441	-4.026595	0.0012316	0.0129493	-0.956743
MYL6B	-1.14664	7.7344399	-4.02624	0.0012325	0.0129493	-0.957427
FASTKD5	-1.192141	5.6430902	-4.026015	0.001233	0.0129493	-0.957859
TRIP4	-1.200315	6.081423	-4.023894	0.0012382	0.0129781	-0.961943
PKIA	-1.450476	7.2443341	-4.023226	0.0012398	0.0129819	-0.963228
MAGEE1	-1.371505	8.1816973	-4.021214	0.0012447	0.0129992	-0.967104
OSR1	-1.320433	3.4911107	-4.020828	0.0012457	0.0129992	-0.967847
ADPRHL2	-1.129427	7.3489332	-4.020317	0.0012469	0.0129992	-0.96883
SORCS1	-1.147285	8.2236746	-4.01985	0.0012481	0.0130019	-0.96973
MIR4313	-1.246755	5.2274131	-4.018398	0.0012516	0.0130258	-0.972525
LOC1001294	-1.062715	3.4869717	-4.010059	0.0012724	0.0131411	-0.988583
C5orf34	-1.620967	3.1611761	-4.008377	0.0012766	0.0131647	-0.991823
TAX1BP1	-1.21035	7.2143313	-4.004849	0.0012855	0.0132165	-0.99862
CXorf23	-1.090694	3.404681	-4.00311	0.0012899	0.0132418	-1.001968
COPRS	-1.014874	7.5521081	-3.996201	0.0013075	0.0133296	-1.01528
NR2F1	-1.467189	7.6444146	-3.992643	0.0013167	0.0134001	-1.022134
SOCS5	-1.211841	4.5996616	-3.99251	0.0013171	0.0134001	-1.02239
LRIF1	-1.096518	2.7569379	-3.991045	0.0013209	0.0134122	-1.025214
CYB5R4	-1.020507	4.8245306	-3.9907	0.0013218	0.0134122	-1.025878
NTSR1	-1.180464	5.0870319	-3.989533	0.0013248	0.0134255	-1.028128
ELTD1	1.429073	4.4064941	3.9890246	0.0013262	0.0134323	-1.029107
CMTM8	-1.431259	4.0567604	-3.985038	0.0013366	0.0134915	-1.03679
LOC1005065	-1.441878	6.3609389	-3.983683	0.0013402	0.0135142	-1.0394
IGK	-1.022099	5.4922528	-3.981445	0.0013461	0.0135283	-1.043715
COMMD9	-1.00273	7.4026073	-3.980663	0.0013482	0.0135414	-1.045221
IMMP1L	-1.439373	5.051809	-3.975476	0.001362	0.0136471	-1.055221
PMEL	-1.012685	5.1106545	-3.975117	0.001363	0.01365	-1.055912
LGMN	-1.171206	5.3538511	-3.970456	0.0013756	0.0137171	-1.064898
ARMC8	-1.030666	5.5930473	-3.967349	0.001384	0.01375	-1.070888
AK5	-1.019287	10.401096	-3.96505	0.0013903	0.01375	-1.075321
FGF19	-1.023539	3.4216155	-3.964998	0.0013905	0.01375	-1.075422
IARS2	-1.108161	5.3680098	-3.959016	0.001407	0.0138658	-1.086956
ADAMTSL4	-1.009339	5.1095711	-3.958118	0.0014095	0.0138658	-1.088689
NICN1	-1.082129	7.8560324	-3.957443	0.0014113	0.0138658	-1.089991
MRPS23	-1.158984	6.2958943	-3.957372	0.0014115	0.0138658	-1.090127
DPM2	-1.07911	6.6509892	-3.956934	0.0014127	0.0138658	-1.090971
LOC1027239	-1.131839	7.803197	-3.956102	0.0014151	0.0138727	-1.092576
FYTTD1	-1.008896	4.0756175	-3.953854	0.0014214	0.0139082	-1.096912
DYX1C1	-1.048167	3.2672955	-3.951596	0.0014277	0.0139564	-1.101267
WSCD2	-1.017632	5.6892299	-3.949967	0.0014323	0.0139946	-1.104409
C20orf85	-1.174009	3.1582164	-3.949062	0.0014349	0.0140052	-1.106155
GAL3ST4	-1.026336	4.0774232	-3.948454	0.0014366	0.0140052	-1.107328
FXYD6	-1.29068	10.13713	-3.948357	0.0014368	0.0140052	-1.107514
PHOX2B	-1.072403	3.3441888	-3.945391	0.0014453	0.0140611	-1.113237
DEPDC5	-1.084627	6.000556	-3.944805	0.001447	0.0140706	-1.114366
CHRDL2	-1.186549	4.7276758	-3.943874	0.0014496	0.0140787	-1.116164

IFT80	-1.029757	4.6166201	-3.93667	0.0014704	0.0142375	-1.130062
LOC1019276	-1.096482	4.5436743	-3.93489	0.0014755	0.0142605	-1.133496
IFIT5	-1.17298	5.8603829	-3.934572	0.0014765	0.0142628	-1.13411
TEX29	-1.042734	5.6522824	-3.927863	0.0014962	0.0143985	-1.147057
HAGH	-1.069283	7.7981102	-3.927862	0.0014962	0.0143985	-1.147058
LINC01049	-1.105389	3.6439444	-3.925315	0.0015037	0.0144507	-1.151974
GNL2	-1.065935	6.4108396	-3.923663	0.0015086	0.0144831	-1.155162
DMXL1	-1.241312	6.061409	-3.919988	0.0015196	0.0145217	-1.162256
USMG5	-1.024641	10.497621	-3.915798	0.0015322	0.014615	-1.170344
FLJ30403	-1.080241	4.8614134	-3.914492	0.0015362	0.0146247	-1.172864
GORAB	-1.776565	4.4720232	-3.913607	0.0015389	0.0146247	-1.174572
SLC12A7	-1.064914	6.2876727	-3.912412	0.0015425	0.014637	-1.17688
RPRM	-1.122336	6.5357083	-3.905919	0.0015624	0.0147491	-1.189415
ZNF571	1.0985379	4.3011225	3.9054693	0.0015638	0.0147491	-1.190283
UQCRFS1	-1.028441	8.4769938	-3.903106	0.0015711	0.0147794	-1.194847
RFWD3	-1.193692	4.8322021	-3.902268	0.0015737	0.0147903	-1.196465
LOC1027235	-1.094435	3.6578771	-3.9013	0.0015767	0.0147982	-1.198333
C1orf109	-1.166705	4.1893911	-3.897703	0.001588	0.0148628	-1.205278
LOC1009963	-1.148351	4.8571626	-3.893343	0.0016017	0.0149639	-1.2137
UNC50	-1.324454	6.55684	-3.887954	0.0016189	0.0150761	-1.224108
CGREF1	-1.187819	5.675792	-3.885335	0.0016273	0.0151337	-1.229166
INHBE	-1.006048	2.8814485	-3.882973	0.0016349	0.0151908	-1.233728
STARD3NL	-1.684884	5.645459	-3.882336	0.001637	0.015203	-1.234958
YPEL4	-1.137259	7.0529839	-3.878496	0.0016494	0.015267	-1.242375
GLIS2	-1.041366	6.019933	-3.868114	0.0016837	0.0154251	-1.262435
ID2	-1.477047	5.9902513	-3.864797	0.0016947	0.0154658	-1.268843
OR2B6	-1.173332	3.2021005	-3.862583	0.0017022	0.0154833	-1.273122
EYA1	-1.00033	4.5148172	-3.862346	0.001703	0.0154833	-1.273579
ZNF331	-1.070446	8.0872151	-3.8621	0.0017038	0.0154833	-1.274054
RAPGEF4-AS	-1.36735	4.9162219	-3.861976	0.0017042	0.0154833	-1.274294
IPPK	-1.04211	6.279517	-3.848718	0.0017495	0.0156954	-1.299916
OR5P3	-1.203027	3.1638546	-3.846342	0.0017578	0.0157461	-1.304509
PCDH17	-1.201901	7.1695586	-3.84588	0.0017594	0.0157536	-1.305401
ACOT13	-1.304203	7.964814	-3.843775	0.0017667	0.0157932	-1.309471
TIPRL	-1.222667	6.8976225	-3.837045	0.0017904	0.0159201	-1.322481
TBL1XR1	-1.165932	6.2942005	-3.835905	0.0017945	0.0159349	-1.324683
MB21D2	-1.127756	6.6679807	-3.830297	0.0018145	0.0160159	-1.335525
ZBED5-AS1	-1.000161	5.1595924	-3.829144	0.0018187	0.0160318	-1.337754
CPOX	-1.336551	3.4537742	-3.825842	0.0018306	0.0161024	-1.344138
MRPS36	-1.254311	5.536473	-3.819423	0.001854	0.0162038	-1.356551
NRSN1	-1.498322	9.4869398	-3.81419	0.0018733	0.0162754	-1.366669
LSM1	-1.263608	7.1039235	-3.812174	0.0018808	0.0163235	-1.370569
PAK7	-1.209339	6.462916	-3.808733	0.0018937	0.0163908	-1.377224
GALNT7	-1.187642	4.8610304	-3.808697	0.0018938	0.0163908	-1.377293
SDF2	-1.02593	6.7045676	-3.807335	0.001899	0.0164074	-1.379927
DRD1	-1.203778	5.5793999	-3.806541	0.0019019	0.0164172	-1.381463
ENPP7	-1.030896	4.6106243	-3.805172	0.0019071	0.0164431	-1.38411
OCIAD2	-1.165681	7.749418	-3.803776	0.0019124	0.016464	-1.38681
GPR31	-1.031892	4.3929639	-3.80293	0.0019156	0.0164689	-1.388447
LOC338963	-1.048355	3.9230733	-3.802261	0.0019181	0.0164689	-1.38974
PGRMC2	-1.116333	6.2561721	-3.799901	0.0019271	0.0164907	-1.394306

ZFP14	-1.190533	6.7238711	-3.797445	0.0019365	0.0165366	-1.399055
ERMP1	-1.321753	5.6386241	-3.793356	0.0019523	0.0166157	-1.406964
VAT1L	-1.664372	7.829945	-3.7924	0.001956	0.0166333	-1.408815
USP28	-1.154932	6.1734515	-3.790756	0.0019624	0.016667	-1.411996
LOC1019283	-1.125422	4.9193	-3.78948	0.0019674	0.0166899	-1.414463
LY96	-1.23808	3.1602029	-3.789127	0.0019687	0.0166932	-1.415146
EFNA3	-1.057914	6.3143498	-3.788793	0.0019701	0.0166973	-1.415791
ELOVL2	1.1592819	4.7536382	3.7884983	0.0019712	0.0167001	-1.416362
MYADM	-1.001265	7.4883858	-3.785786	0.0019818	0.0167455	-1.42161
ACKR1	-1.233285	6.4602311	-3.78563	0.0019824	0.0167455	-1.421911
CLVS2	-1.416404	4.7498594	-3.785594	0.0019826	0.0167455	-1.42198
ADAMTS3	-1.293144	4.0881607	-3.779429	0.002007	0.0168635	-1.433907
ZNF442	-1.274762	4.2542298	-3.777235	0.0020157	0.0169054	-1.438153
HDGFL1	-1.060664	5.2516639	-3.774829	0.0020254	0.0169554	-1.442807
LINC01104	-1.004384	2.9327442	-3.773711	0.0020299	0.0169723	-1.44497
VDAC2	-1.157514	10.026653	-3.768278	0.0020519	0.0170846	-1.455482
MYOT	-1.103829	3.366341	-3.762415	0.0020759	0.017169	-1.466827
TMEM55A	-1.561585	4.1273112	-3.761652	0.002079	0.0171818	-1.468305
DHRS11	-1.049955	5.7196587	-3.756366	0.0021009	0.0172852	-1.478534
MOCS3	-1.134292	3.8423688	-3.750432	0.0021258	0.0174083	-1.490016
SMIM3	-1.273103	5.1256756	-3.750148	0.002127	0.0174083	-1.490567
ZWILCH	-1.102585	5.5799373	-3.750089	0.0021273	0.0174083	-1.49068
MRPL1	-1.213531	6.8664483	-3.748316	0.0021348	0.0174264	-1.494112
SH2D4A	-1.035267	6.5580205	-3.746821	0.0021411	0.0174264	-1.497005
UBE2T	-1.232917	7.5052729	-3.746817	0.0021411	0.0174264	-1.497014
GOLT1B	-1.590911	5.8770719	-3.746791	0.0021413	0.0174264	-1.497063
IBTK	-1.05696	3.9674297	-3.743914	0.0021535	0.0174845	-1.502632
STXBP5L	-1.523631	7.1739763	-3.737973	0.0021791	0.017603	-1.514131
CCK	-1.285851	8.2329527	-3.736965	0.0021834	0.0176086	-1.516081
MLLT4-AS1	-1.010709	3.6797329	-3.726608	0.0022288	0.0178269	-1.536129
SCYL3	-1.326236	5.5213044	-3.720322	0.0022568	0.017952	-1.548298
CYFIP2	-1.010186	9.3148543	-3.719737	0.0022594	0.017952	-1.549429
RAB2A	-1.000026	7.3526926	-3.719435	0.0022608	0.017952	-1.550015
DNAJB9	-1.197313	4.9450305	-3.716614	0.0022735	0.0179805	-1.555475
KIAA0087	-1.41823	4.901172	-3.713906	0.0022858	0.0180426	-1.560718
FAM102B	-1.671293	6.8929823	-3.71075	0.0023001	0.0180927	-1.566828
TMEM41B	-1.055278	6.0466566	-3.709369	0.0023065	0.0181082	-1.569501
DOC2B	-1.068371	4.7100397	-3.707424	0.0023154	0.0181435	-1.573266
LOC1019271	-1.10057	5.0136054	-3.706654	0.0023189	0.0181622	-1.574756
PSPH	-1.058053	3.4727768	-3.703649	0.0023328	0.0182104	-1.580574
NAPEPLD	-1.085918	5.4826308	-3.700806	0.002346	0.0182701	-1.586079
CMC1	-1.07648	3.4979185	-3.694075	0.0023776	0.0184325	-1.59911
HHIPL2	-1.10374	4.0578706	-3.693824	0.0023788	0.0184325	-1.599597
ZNF281	-1.332043	6.0837418	-3.691733	0.0023887	0.0184805	-1.603644
THEG5	-1.213405	5.6013135	-3.688902	0.0024022	0.0185403	-1.609126
OSTF1	-1.287019	6.4679163	-3.688182	0.0024056	0.0185454	-1.61052
PRPF39	-1.776024	3.8441945	-3.684069	0.0024254	0.0186275	-1.618484
MLYCD	-1.001187	5.3843178	-3.683328	0.002429	0.0186409	-1.619919
PCMT1	-1.118159	9.2015397	-3.68293	0.0024309	0.0186417	-1.62069
LINC01342	-1.1275	4.8791576	-3.679936	0.0024454	0.0186899	-1.626486
IGIP	-1.100695	7.9932827	-3.678162	0.002454	0.0187395	-1.629921

PRO1596	-1.49148	5.5908005	-3.677965	0.002455	0.0187395	-1.630303
SRBD1	-1.158246	4.8520912	-3.670324	0.0024926	0.0189152	-1.645097
KLHL42	-1.002142	7.0974157	-3.670282	0.0024928	0.0189152	-1.64518
LOC1019285	-1.038364	5.9907763	-3.664335	0.0025225	0.019023	-1.656694
GLRX	-1.228661	7.0154796	-3.660858	0.00254	0.0191058	-1.663428
TNF	-1.022926	3.2526761	-3.660107	0.0025438	0.0191062	-1.664881
ARMCX2	-1.291768	6.9036184	-3.653365	0.0025781	0.019245	-1.677938
R3HDM1	-1.076832	7.2752929	-3.652882	0.0025806	0.019245	-1.678872
NDUFS2	-1.101665	7.5172045	-3.652837	0.0025808	0.019245	-1.678959
RALY	-1.047085	8.1214541	-3.652063	0.0025848	0.0192589	-1.680457
CACNG3	-1.059447	6.9755197	-3.648521	0.0026031	0.019344	-1.687317
TMEM17	-1.238427	4.0431332	-3.647917	0.0026062	0.019355	-1.688487
CMTM5	-1.014379	5.7195042	-3.64614	0.0026154	0.0194024	-1.691928
NCKAP1	-1.177081	6.9255931	-3.644467	0.0026242	0.0194319	-1.695167
EPHX4	-1.390869	6.2366768	-3.643659	0.0026284	0.0194319	-1.696733
LOC1027239	1.0275175	4.3727234	3.643377	0.0026299	0.0194319	-1.697278
LOC283357	-1.308887	3.9730815	-3.642201	0.002636	0.0194423	-1.699555
HIST1H4G	-1.004246	3.1524884	-3.641343	0.0026405	0.0194615	-1.701216
BAG5	-1.086681	5.6870073	-3.641021	0.0026422	0.0194635	-1.70184
ARF4	-1.132854	7.3219595	-3.640254	0.0026462	0.0194697	-1.703325
TTC13	-1.05532	5.0543714	-3.640092	0.0026471	0.0194697	-1.703639
MKNK1-AS1	1.1727349	4.3569125	3.6400457	0.0026473	0.0194697	-1.703729
ZNF436	-1.11964	6.6178224	-3.638958	0.0026531	0.0194769	-1.705836
RAB2B	-1.064985	6.5295991	-3.636226	0.0026675	0.0195495	-1.711126
EML1	-1.129962	4.5923025	-3.636052	0.0026685	0.0195495	-1.711462
NDUFB1	-1.000953	6.2010991	-3.635522	0.0026713	0.0195614	-1.71249
GPR144	-1.059001	5.0393029	-3.634543	0.0026765	0.0195772	-1.714384
SMAGP	-1.039556	3.7344591	-3.634397	0.0026773	0.0195772	-1.714668
OARD1	-1.101423	6.1487981	-3.633052	0.0026844	0.0196016	-1.717271
PEG10	-1.434639	6.4305367	-3.632524	0.0026873	0.0196152	-1.718294
ASUN	-1.391251	4.9229206	-3.63095	0.0026957	0.0196628	-1.721342
MAK16	-1.192626	5.4533249	-3.624671	0.0027296	0.0198177	-1.733501
GIMAP7	-1.545279	3.7536499	-3.619791	0.0027563	0.019948	-1.742952
MTMR4	-1.032308	7.117267	-3.613178	0.0027928	0.0201168	-1.755757
CYTIP	-1.378502	3.6407714	-3.612564	0.0027962	0.0201168	-1.756946
BDNF	-1.222295	5.6080308	-3.610503	0.0028077	0.0201846	-1.760937
CRIPT	-1.195998	6.6565922	-3.609553	0.002813	0.0201873	-1.762777
CDK5R2	-1.031296	7.7339772	-3.607589	0.002824	0.020238	-1.76658
CRHBP	-1.150634	5.9093584	-3.603134	0.0028492	0.0203531	-1.775208
MRPL46	-1.191638	5.7453026	-3.596063	0.0028896	0.0205595	-1.788901
RPL9	-1.06632	10.250205	-3.591433	0.0029164	0.0206538	-1.797865
SP3	-1.016069	5.8626775	-3.591072	0.0029185	0.0206603	-1.798565
GALNT15	-1.113112	4.4303932	-3.577737	0.002997	0.0209854	-1.824386
SOCS2-AS1	-1.304008	3.7028488	-3.576856	0.0030023	0.0209869	-1.826091
LOC1005071	-1.311197	6.3576284	-3.569872	0.0030444	0.0211262	-1.839615
FSTL5	-1.703845	4.734518	-3.569762	0.003045	0.0211262	-1.839827
GNG3	-1.110069	9.079278	-3.569353	0.0030475	0.0211291	-1.840619
LOC1025462	-1.013177	6.3169463	-3.565732	0.0030696	0.0211959	-1.847631
NAT14	-1.097463	7.1609562	-3.565346	0.0030719	0.0212051	-1.848377
OCA2	-1.036262	8.4993907	-3.558796	0.0031123	0.0213975	-1.861059
UGDH	-1.35914	5.1361469	-3.557557	0.00312	0.0214125	-1.863459

SCGB3A1	-1.076333	5.9041999	-3.556709	0.0031253	0.0214143	-1.865101
WFDC1	-1.165487	5.661843	-3.552292	0.0031529	0.0215675	-1.873653
YWHAB	-1.14554	10.788628	-3.551792	0.003156	0.0215746	-1.874619
LOC1019287	-1.00041	2.5905592	-3.550226	0.0031659	0.0216006	-1.877652
PLEKHG1	1.0455525	3.9990882	3.5489973	0.0031737	0.0216006	-1.880031
NIPA1	-1.111865	8.5957393	-3.542375	0.0032158	0.0217724	-1.892852
LCN6	-1.061485	4.1026748	-3.536708	0.0032524	0.0219503	-1.903821
MKX	-1.102429	4.4735235	-3.521803	0.0033505	0.0223365	-1.932674
POC5	-1.188301	6.3026999	-3.51961	0.0033652	0.022383	-1.936918
COPG2IT1	-1.055062	9.4695747	-3.518737	0.003371	0.022383	-1.938609
LOC286382	-1.000474	4.8345132	-3.515572	0.0033924	0.0224698	-1.944735
LINC01010	-1.239445	4.5764298	-3.514894	0.0033969	0.0224706	-1.946046
C10orf35	-1.113689	7.8895681	-3.51357	0.0034059	0.0225082	-1.948609
YARS2	-1.25685	5.4170619	-3.511311	0.0034213	0.0225515	-1.95298
LOC1019278	-1.020341	4.8080382	-3.508172	0.0034428	0.0225965	-1.959057
LOC1009966	-1.001704	3.8720875	-3.505049	0.0034643	0.022696	-1.9651
CHGB	-1.778131	7.8477489	-3.503759	0.0034732	0.0227325	-1.967598
PRRT2	-1.008248	7.3270078	-3.501983	0.0034855	0.0227802	-1.971033
LOC101927C	-1.05657	4.5119548	-3.501535	0.0034887	0.0227802	-1.971901
TSC1	-1.158185	8.048881	-3.501066	0.0034919	0.0227802	-1.972809
MRPL19	-1.111566	5.2642667	-3.500672	0.0034947	0.0227802	-1.973571
NAPA	-1.018052	6.8462581	-3.500501	0.0034959	0.0227802	-1.973903
SDHD	-1.132205	4.1468866	-3.500486	0.003496	0.0227802	-1.973931
HRASLS5	-1.125399	5.4856168	-3.50047	0.0034961	0.0227802	-1.973962
OR3A3	-1.085746	4.4804496	-3.499821	0.0035006	0.0227952	-1.975218
PIK3CB	-1.048173	6.8115812	-3.496542	0.0035236	0.0228937	-1.981563
LOC644656	-1.144121	5.6550901	-3.491192	0.0035614	0.0230209	-1.991916
NPTXR	-1.087399	6.8731677	-3.48787	0.0035851	0.0230733	-1.998344
TMEM155	-1.42702	6.6178887	-3.487027	0.0035911	0.0230771	-1.999975
LPPR5	-1.220028	5.6189666	-3.482595	0.003623	0.0231934	-2.008551
ST6GALNAC5	-1.338941	4.9335981	-3.482345	0.0036248	0.0231977	-2.009033
RPA3OS	-1.406431	3.5838983	-3.474904	0.003679	0.0234566	-2.023431
TUSC1	-1.385306	6.845433	-3.474527	0.0036818	0.0234596	-2.02416
CLK4	-1.037429	6.5196645	-3.47111	0.0037069	0.0235467	-2.030771
HMGCLL1	-1.02952	4.5954922	-3.468525	0.0037261	0.0236171	-2.03577
ZNF524	-1.031167	5.5101787	-3.46834	0.0037275	0.0236185	-2.036129
RNF181	-1.067357	6.980257	-3.46797	0.0037302	0.0236195	-2.036845
PAIP1	-1.061266	6.5591206	-3.467333	0.003735	0.0236367	-2.038076
GNAT2	-1.017954	4.4682459	-3.465499	0.0037487	0.0236989	-2.041625
P4HTM	-1.024157	7.6839588	-3.462901	0.0037681	0.0237636	-2.046649
DOK4	-1.066645	6.3453081	-3.460444	0.0037867	0.0238385	-2.051402
ANKRD12	-1.154157	6.4454635	-3.454605	0.003831	0.0239704	-2.062696
SLTM	-1.44233	5.2635222	-3.454391	0.0038327	0.0239733	-2.06311
LOC1019306	-1.059184	4.3033668	-3.454072	0.0038351	0.0239766	-2.063727
HDHD1	-1.3008	6.7329079	-3.454016	0.0038355	0.0239766	-2.063835
PAN3	-1.61654	6.8585665	-3.452067	0.0038505	0.0240186	-2.067604
TXN	-1.058048	8.7754931	-3.450025	0.0038662	0.0240688	-2.071553
MAP10	-1.245026	2.7242243	-3.448832	0.0038754	0.0241153	-2.07386
TIMM21	-1.346557	6.2460164	-3.442557	0.0039243	0.0242863	-2.085996
C12orf29	-1.248985	6.7702325	-3.441021	0.0039363	0.0243153	-2.088965
DEGS2	-1.147814	5.2404118	-3.439538	0.003948	0.0243462	-2.091833

GPR34	-1.463953	3.0449026	-3.436141	0.0039748	0.024438	-2.098401
PNMA1	-1.174337	8.1745331	-3.435961	0.0039763	0.024438	-2.098749
EPHA5	-1.018906	5.1411677	-3.435666	0.0039786	0.0244451	-2.099319
TRIM32	-1.172097	5.9457668	-3.429127	0.0040309	0.0246551	-2.111961
KCNH8	-1.491543	3.4972468	-3.428322	0.0040373	0.0246768	-2.113519
LOC1019272	-1.0243	4.9048813	-3.428237	0.004038	0.0246768	-2.113682
ANKRD13C	-1.05241	4.9504048	-3.424085	0.0040716	0.0247873	-2.121709
TUSC7	-1.191835	5.0639373	-3.423124	0.0040794	0.0248113	-2.123566
NEFL	-1.361891	9.5097004	-3.422011	0.0040885	0.0248586	-2.125719
UBE2Q2	-1.532314	5.8163157	-3.416272	0.0041356	0.0250637	-2.136812
TDRD9	-1.30259	4.1208796	-3.415553	0.0041415	0.0250772	-2.138201
RASGEF1C	-1.096616	5.0913516	-3.413757	0.0041564	0.0251325	-2.141672
GALNTL5	-1.104693	4.8928939	-3.413711	0.0041568	0.0251325	-2.141761
CTTNBP2	-1.633793	4.9154354	-3.406218	0.0042194	0.0253331	-2.156243
SIMC1	-1.2418	4.5955822	-3.403189	0.004245	0.0254552	-2.162097
POLI	-1.134668	5.1925564	-3.400843	0.0042649	0.0255447	-2.166628
EIF3M	-1.018105	4.8190271	-3.397818	0.0042908	0.0256319	-2.172474
F11-AS1	-1.064687	3.0591424	-3.39714	0.0042966	0.0256487	-2.173783
LOC284513	-1.393394	6.463922	-3.390371	0.004355	0.0258508	-2.186862
YTHDF1	-1.489387	8.5108192	-3.39035	0.0043552	0.0258508	-2.186902
SLC1A1	-1.698527	6.6017005	-3.384549	0.0044059	0.0260594	-2.198108
PSMG3	-1.127292	6.8665215	-3.382921	0.0044203	0.0261004	-2.201253
TBCA	-1.126592	8.5777666	-3.382131	0.0044273	0.0261118	-2.202779
RIT2	-1.670237	5.5744239	-3.380083	0.0044454	0.0261509	-2.206734
KHDRBS1	-1.077555	6.8178654	-3.377026	0.0044726	0.0262506	-2.212639
TBR1	-1.051162	6.9697551	-3.373546	0.0045038	0.0263434	-2.219359
FAM122B	-1.21886	5.1414008	-3.372508	0.0045131	0.02639	-2.221363
LOC1001281	-1.121964	3.4708335	-3.371405	0.0045231	0.026418	-2.223493
NIPSNAP3A	-1.502205	4.494984	-3.368558	0.0045489	0.0264779	-2.228991
P2RY1	1.0646628	3.8396707	3.3666234	0.0045665	0.0265275	-2.232727
CARTPT	-1.092237	6.1775949	-3.364929	0.0045819	0.0265646	-2.235997
GALNT14	-1.192979	5.0041279	-3.364694	0.0045841	0.0265696	-2.236453
WASF1	-1.927115	8.9106671	-3.362409	0.0046051	0.0266307	-2.240864
NANP	-1.177544	4.0863904	-3.361148	0.0046167	0.0266687	-2.243298
CBX1	-1.033014	8.7541519	-3.356354	0.0046611	0.0267938	-2.252553
COPS4	-1.309267	4.9131244	-3.354963	0.004674	0.0268473	-2.255237
TTC30A	-1.033282	3.8245736	-3.345863	0.0047597	0.0271112	-2.272802
RLIM	-1.142733	7.5869115	-3.345457	0.0047636	0.0271198	-2.273585
HSPA12A	-1.005617	7.3266839	-3.342522	0.0047916	0.0271845	-2.27925
SPOP	-1.036432	7.3876407	-3.340495	0.004811	0.02724	-2.283159
ANXA3	-1.867277	3.7971301	-3.340051	0.0048152	0.0272531	-2.284016
SLC10A2	-1.09028	4.2194584	-3.339682	0.0048188	0.0272657	-2.284729
LOC1019274	-1.017764	2.9318788	-3.337057	0.0048441	0.0273161	-2.289794
FABP5	-1.000599	7.9673819	-3.331847	0.0048948	0.027445	-2.299845
PCNP	-1.022537	6.9284769	-3.330807	0.0049049	0.027487	-2.301852
GPR158	-1.640488	8.1477995	-3.329234	0.0049204	0.0275207	-2.304887
FAM220A	-1.63103	6.6015771	-3.326078	0.0049515	0.0276342	-2.310973
EIF1AY	-1.455043	3.1427191	-3.325038	0.0049618	0.0276639	-2.31298
FRMPD4	-1.435401	6.5583164	-3.32383	0.0049737	0.0276981	-2.31531
MUT	-1.371802	6.8357318	-3.322043	0.0049915	0.0277517	-2.318756
APOBEC4	-1.327115	4.5227499	-3.320338	0.0050085	0.0278313	-2.322045

ARPC3	-1.095604	7.2088401	-3.318599	0.005026	0.0278897	-2.325398
CRYM	-1.112182	9.6302227	-3.316209	0.00505	0.027978	-2.330007
LOC100506C	-1.00722	2.8331134	-3.315907	0.005053	0.0279798	-2.33059
DLD	-1.111407	5.5792633	-3.312472	0.0050878	0.0280584	-2.337212
BBS10	-1.095196	3.8109515	-3.308162	0.0051318	0.0282063	-2.345523
SCN2A	-1.152808	8.7593929	-3.302061	0.0051947	0.0284027	-2.357283
LYSMD4	-1.00755	5.4025543	-3.297971	0.0052373	0.0285573	-2.365166
PRIMA1	-1.328239	5.8161237	-3.297737	0.0052397	0.0285573	-2.365617
PDHX	-1.447857	6.3104153	-3.297417	0.005243	0.0285622	-2.366234
SELL	-1.157805	3.5291505	-3.296851	0.005249	0.0285622	-2.367324
TMEM161B-A	-1.162892	6.4746398	-3.296851	0.005249	0.0285622	-2.367324
NELL2	-1.584767	9.1669324	-3.293161	0.0052878	0.0286709	-2.374436
MRPL54	-1.226372	5.8828291	-3.291481	0.0053055	0.0287173	-2.377673
C6orf106	-1.027009	7.7693967	-3.290239	0.0053187	0.0287582	-2.380067
TMEM38A	-1.04534	5.6697232	-3.286272	0.005361	0.0289047	-2.38771
NADK2-AS1	-1.17201	4.9069291	-3.285041	0.0053742	0.0289448	-2.39008
TMEM5	-1.135785	4.4745318	-3.284112	0.0053842	0.0289448	-2.39187
NDFIP1	-1.366371	8.2195216	-3.280824	0.0054196	0.0290428	-2.398204
RAN	-1.01118	7.1705748	-3.279315	0.005436	0.029077	-2.401112
SPA17	-1.017243	5.5443668	-3.277398	0.0054568	0.0291354	-2.404804
LINC00616	1.0493897	4.4391507	3.2741438	0.0054924	0.0292717	-2.411071
GSKIP	-1.538875	6.495657	-3.272836	0.0055067	0.0293101	-2.41359
LOC1019292	-1.037479	3.7241362	-3.272551	0.0055099	0.0293191	-2.414139
SPRYD4	-1.105725	6.2671973	-3.267887	0.0055614	0.0294646	-2.423119
TMEM251	-1.245958	3.3733423	-3.258122	0.0056709	0.0298191	-2.441917
MAGEH1	-1.388929	7.4053295	-3.256016	0.0056948	0.029891	-2.445972
HOMER1	-1.200099	7.0025895	-3.255499	0.0057006	0.0298911	-2.446966
CCDC113	-1.002572	7.0193461	-3.249133	0.0057736	0.0301344	-2.459218
TXNDC12	-1.383387	8.5215013	-3.243423	0.0058397	0.0303097	-2.470203
DHRS7B	-1.046837	7.096107	-3.240505	0.0058738	0.0304404	-2.475817
MDH1	-1.121623	9.2959303	-3.239281	0.0058882	0.0304917	-2.478171
BIRC2	-1.370765	6.5071736	-3.238732	0.0058947	0.0305174	-2.479227
JAGN1	-1.011532	6.9957216	-3.235013	0.0059386	0.0306299	-2.48638
STXBP5	-1.160107	5.4461731	-3.233431	0.0059573	0.0306944	-2.489422
SNX30	-1.551299	5.703865	-3.230605	0.005991	0.0307894	-2.494857
TREM1	-1.01525	3.4604238	-3.230518	0.0059921	0.0307894	-2.495025
RXFP1	-1.552054	5.9212292	-3.230244	0.0059954	0.0307894	-2.49555
SLITRK1	-1.190344	7.7417266	-3.226941	0.006035	0.0308845	-2.501902
DHCR24	-1.292437	8.1918471	-3.22646	0.0060408	0.0308907	-2.502826
GHET1	1.0627045	4.0118098	3.225939	0.0060471	0.0309077	-2.503828
AMPH	-1.316151	8.4454997	-3.224322	0.0060666	0.030962	-2.506937
DNAJC6	-1.262238	6.8150491	-3.217189	0.0061536	0.0312569	-2.520648
SLC25A3P1	1.6607095	3.8145209	3.2166419	0.0061603	0.0312685	-2.521699
SLC30A10	-1.024471	3.2990566	-3.215651	0.0061725	0.0312975	-2.523603
LOC1001302	-1.283092	3.6943758	-3.215092	0.0061794	0.0313065	-2.524678
CSGALNACT2	-1.076076	5.5607699	-3.214578	0.0061858	0.0313211	-2.525664
EEFSEC	-1.0688	6.1105224	-3.213961	0.0061934	0.031348	-2.526851
SYNPR	-1.413369	8.5937641	-3.212416	0.0062125	0.0313846	-2.529819
ABRACL	-1.146565	6.4487437	-3.197591	0.006399	0.0319638	-2.558299
SDCBP	-1.333087	8.1515257	-3.195185	0.0064298	0.0320628	-2.562917
ST8SIA1	-1.086855	4.3870232	-3.188018	0.0065224	0.0323589	-2.576678

KCNT2	-1.232995	4.9659437	-3.186113	0.0065472	0.032412	-2.580336
COX14	-1.015801	7.2349033	-3.183815	0.0065773	0.032495	-2.584746
RAB38	-1.009263	2.9181237	-3.182459	0.0065951	0.0325619	-2.587348
SH3GL2	-1.207561	9.035761	-3.172097	0.0067329	0.033003	-2.60723
LOC1019291	-1.044701	4.2672648	-3.170523	0.006754	0.0330513	-2.610248
BEX1	-1.323618	9.9801636	-3.169842	0.0067632	0.0330805	-2.611556
KIAA1045	-1.11874	7.3489979	-3.166879	0.0068033	0.0331813	-2.617238
LINC01361	-1.09927	3.7436237	-3.163657	0.0068472	0.0333427	-2.623417
RABGGTB	-1.009807	6.8482115	-3.159291	0.006907	0.0335401	-2.631788
NAA30	-1.106883	5.9634295	-3.156959	0.0069393	0.0336538	-2.63626
TRIL	-1.558577	5.7632961	-3.155396	0.0069609	0.0337089	-2.639256
FBXL2	-1.131279	7.3614844	-3.149747	0.0070398	0.0339088	-2.650081
DGKI	-1.226048	6.9597946	-3.141261	0.0071599	0.0342161	-2.666341
MARS2	-1.069663	6.9288476	-3.141186	0.007161	0.0342161	-2.666484
RANBP6	-1.208602	6.1939735	-3.140153	0.0071757	0.0342546	-2.668463
LHFP	-1.158171	7.0123223	-3.139326	0.0071876	0.0342712	-2.670048
EXOSC6	-1.108048	6.1267558	-3.134953	0.0072505	0.0344909	-2.678423
SRD5A1	-1.219928	5.4081374	-3.131538	0.0073	0.0346621	-2.684962
MRPL13	-1.161502	5.1700486	-3.12539	0.0073901	0.0349364	-2.696732
RAB9B	-1.586085	7.2398854	-3.125373	0.0073903	0.0349364	-2.696763
PPP1R14A	-1.782943	6.6425139	-3.122935	0.0074263	0.0350051	-2.701429
PADI3	-1.03294	3.520929	-3.111794	0.007593	0.0354284	-2.722746
GTF2I	-1.341957	8.6829266	-3.111668	0.0075949	0.0354292	-2.722986
LINC00888	-1.292646	6.7350124	-3.109894	0.0076219	0.0355143	-2.726381
ZCCHC9	-1.279303	5.1100353	-3.107539	0.0076577	0.0356164	-2.730883
C11orf74	-1.504374	5.9360197	-3.106564	0.0076726	0.0356695	-2.732749
RTN1	-1.032	11.689144	-3.10557	0.0076878	0.0356996	-2.73465
SMIM24	-1.612789	5.8702902	-3.104595	0.0077028	0.0357528	-2.736513
FUNDC2	-1.058473	6.5968334	-3.102076	0.0077415	0.0358595	-2.74133
MTERF3	-1.280313	5.88018	-3.098167	0.007802	0.0360664	-2.748801
ARHGAP5-AS	-1.061351	7.2636348	-3.097979	0.007805	0.0360718	-2.749162
BNIP2	-1.079864	4.8000083	-3.09759	0.007811	0.0360916	-2.749905
WBP4	-1.022629	6.804627	-3.097215	0.0078169	0.0361063	-2.750621
MIRLET7DHG	-1.133949	4.1790556	-3.095465	0.0078442	0.0361956	-2.753966
FNBP1L	-1.222375	6.1177479	-3.094678	0.0078565	0.0362361	-2.755471
GNG10	-1.041751	7.7726705	-3.092536	0.0078901	0.0362847	-2.759563
SYNC	-1.230367	3.5494048	-3.08668	0.0079826	0.0365953	-2.770751
NME1	-1.23371	7.5351181	-3.085536	0.0080009	0.0366419	-2.772937
RAI2	-1.025505	5.085145	-3.084495	0.0080175	0.0366727	-2.774925
LPPR4	-1.321868	5.6769074	-3.083145	0.0080391	0.0367433	-2.777503
TMEM128	-1.275322	5.3356044	-3.082979	0.0080417	0.0367433	-2.777821
ZNF432	-1.056885	5.2173096	-3.081824	0.0080602	0.0367944	-2.780026
PELI3	-1.14696	7.2229199	-3.078633	0.0081116	0.036963	-2.78612
MAT2B	-1.226216	6.68102	-3.065296	0.0083299	0.037448	-2.811575
MAS1	-1.187549	4.7370817	-3.062637	0.0083742	0.0375571	-2.816648
EIF2AK2	-1.0855	6.8972703	-3.052792	0.0085399	0.0380917	-2.835425
POMP	-1.210533	6.1429861	-3.049425	0.0085974	0.0382728	-2.841844
SLC12A5	-1.014622	8.2779519	-3.046564	0.0086464	0.0384078	-2.847296
FOXD2-AS1	-1.075923	3.9638803	-3.046177	0.0086531	0.038429	-2.848033
TATDN1	-1.165161	6.9800719	-3.043919	0.0086921	0.038502	-2.852336
NGFRAP1	-1.071331	9.6665227	-3.043243	0.0087038	0.038512	-2.853625

PEX3	-1.002092	5.2577981	-3.042667	0.0087138	0.0385478	-2.854722
SULF1	-1.034083	5.0658758	-3.042478	0.008717	0.038554	-2.855083
LUZP2	-1.124586	4.5170196	-3.038779	0.0087814	0.0387132	-2.862129
ZBTB2	-1.041264	4.2528579	-3.038089	0.0087935	0.0387331	-2.863445
PRDX3	-1.217777	6.94878	-3.03741	0.0088054	0.0387771	-2.864737
PSMA5	-1.11463	6.114016	-3.035796	0.0088337	0.0388309	-2.867812
TMEM14A	-1.520896	7.8666978	-3.035747	0.0088346	0.0388309	-2.867905
BZRAP1-AS1	-1.088021	5.3127521	-3.035528	0.0088384	0.0388309	-2.868323
TOMM5	-1.009458	6.8308274	-3.035429	0.0088402	0.0388309	-2.86851
GPR12	-1.099164	5.0779138	-3.034841	0.0088505	0.0388478	-2.86963
LRP1B	-1.324034	5.1652496	-3.026548	0.0089978	0.0392331	-2.88542
DACH2	-1.462734	5.2792273	-3.022568	0.0090693	0.0394468	-2.892997
GAFA2	-1.022026	5.1819191	-3.022349	0.0090732	0.0394556	-2.893414
SNX17	-1.143301	7.3223316	-3.020889	0.0090996	0.0394626	-2.896192
DPY19L2	-1.255149	5.0684388	-3.019502	0.0091248	0.0395335	-2.89883
ZNF532	-1.031584	7.5597783	-3.019205	0.0091302	0.039535	-2.899395
ESYT2	-1.077454	5.9866913	-3.018339	0.0091459	0.0395865	-2.901044
NUPL1	-1.054836	4.9065859	-3.017796	0.0091558	0.0396041	-2.902077
CRYZ	-1.527747	6.6817877	-3.015514	0.0091974	0.0396919	-2.906417
UTY	-1.247341	5.1457822	-3.008165	0.0093328	0.0400885	-2.920393
MAP2K1	-1.186033	8.4606665	-3.005314	0.0093859	0.0402	-2.925815
TMEM60	-1.405256	5.5829318	-3.004401	0.009403	0.0402327	-2.92755
GNAI1	-1.216754	6.0877183	-3.000433	0.0094774	0.0404225	-2.935092
DCAF17	-1.136606	4.6713994	-2.995089	0.0095786	0.0406386	-2.945244
TMEM246	-1.055887	7.8615533	-2.990997	0.0096569	0.0408138	-2.953017
UHRF2	-1.070662	6.9537047	-2.98512	0.0097703	0.0411149	-2.964177
AGPAT9	-1.753641	5.2801287	-2.981818	0.0098346	0.0413091	-2.970446
PGM3	-1.223418	5.4512619	-2.976086	0.0099473	0.0415604	-2.981323
STMN2	-1.019879	11.064089	-2.971289	0.0100425	0.0418223	-2.990421
ZNF586	-1.078264	5.4910806	-2.96965	0.0100753	0.0419303	-2.99353
PANK1	-1.083409	3.6501122	-2.967927	0.0101098	0.0420079	-2.996799
PRR18	-1.287712	5.5139341	-2.964192	0.0101851	0.0422272	-3.00388
MGC39584	-1.117914	4.0087818	-2.958347	0.010304	0.0424954	-3.014957
ACTR3	-1.061153	6.7291722	-2.957998	0.0103111	0.0425163	-3.015619
HERC2	-1.153221	8.0531907	-2.951303	0.0104491	0.0428606	-3.028302
PTPRT	-1.11981	6.7689998	-2.944959	0.0105815	0.04311	-3.040315
JKAMP	-1.350252	5.8707115	-2.932509	0.0108462	0.043744	-3.063872
LOC157562	-1.330696	7.0170757	-2.931799	0.0108615	0.0437664	-3.065216
CCDC160	-1.216311	3.7808106	-2.931755	0.0108625	0.0437664	-3.065299
WWP1	-1.155159	6.1779987	-2.927143	0.0109623	0.0440386	-3.074019
LOC100505€	-1.23009	4.4879921	-2.926435	0.0109777	0.0440686	-3.075358
UBR7	-1.043849	7.0772669	-2.926075	0.0109855	0.0440686	-3.076038
TPTE2P6	-1.094371	2.9001978	-2.925712	0.0109935	0.0440686	-3.076724
IQCF5	-1.078578	3.4929696	-2.925051	0.0110079	0.0441074	-3.077975
TOP2B	-1.024288	8.1802253	-2.918337	0.0111554	0.0444134	-3.090662
NR3C2	-1.147085	6.8681918	-2.913861	0.0112549	0.0446616	-3.099118
SST	-1.163128	5.6538236	-2.908197	0.011382	0.0449527	-3.109813
EXOSC9	-1.174052	6.3928434	-2.907304	0.0114022	0.0450103	-3.111499
RGS4	-1.239553	8.0552576	-2.906839	0.0114127	0.0450237	-3.112377
RPL22L1	1.0854725	3.9878195	2.8917935	0.0117581	0.0457184	-3.14076
KAT7	-1.148554	7.548148	-2.889385	0.0118143	0.04587	-3.145301

LOC641515	-1.048008	2.7113193	-2.887147	0.0118668	0.0459317	-3.149519
COPS5	-1.300791	5.4919136	-2.8861	0.0118914	0.0459712	-3.151491
VPS13A	-1.025844	5.7852352	-2.885455	0.0119066	0.0460075	-3.152706
TXLNG	-1.097261	6.3736438	-2.883565	0.0119513	0.0461257	-3.156269
EIF1AX	-1.003917	6.5918452	-2.882512	0.0119763	0.0461421	-3.158252
PET117	-1.086929	5.2426759	-2.879256	0.0120537	0.0463096	-3.164386
KTN1	-1.04382	6.5468028	-2.876721	0.0121144	0.0464419	-3.16916
DPY19L2P2	-1.572902	5.7589908	-2.873152	0.0122003	0.0466227	-3.175879
LOC388882	-1.00219	4.9666091	-2.8697	0.012284	0.0467549	-3.182377
SUDS3	-1.019518	6.567759	-2.867435	0.0123392	0.0468471	-3.186639
C9orf57	-1.045827	2.5125409	-2.865214	0.0123935	0.0469664	-3.190818
NEK7	-1.313163	5.8929	-2.856658	0.0126051	0.04743	-3.206908
BPGM	-1.089466	5.9936326	-2.85652	0.0126086	0.04743	-3.207168
ABCC12	-1.148759	4.8604584	-2.855682	0.0126295	0.0474738	-3.208743
PGAP1	-1.046227	5.665464	-2.848411	0.0128125	0.0479413	-3.222406
RHOBTB1	-1.347263	5.4136889	-2.844898	0.0129018	0.0481171	-3.229003
LOC1009967	-1.32389	6.1410315	-2.839949	0.0130287	0.0484226	-3.238295
NEGR1	-1.345747	6.7450469	-2.836393	0.0131206	0.0485877	-3.244969
AUH	-1.891243	5.8998755	-2.829231	0.0133076	0.0489086	-3.258405
TMCO5B	-1.04138	4.7603117	-2.827928	0.0133419	0.0489904	-3.260849
USP9Y	-1.765187	4.5916384	-2.82761	0.0133503	0.0489952	-3.261446
APLNR	1.6755598	4.9665789	2.8259222	0.0133949	0.049073	-3.26461
PSMB6	-1.239639	6.492839	-2.825858	0.0133966	0.049073	-3.26473
C3orf14	-1.272224	6.0944801	-2.819761	0.013559	0.049424	-3.276158
LOC1005055	-1.187749	5.0493673	-2.817113	0.0136301	0.0496036	-3.281119
SERPINI1	-1.688345	9.270398	-2.816194	0.0136549	0.0496673	-3.28284
CADPS2	-1.457863	8.7595312	-2.815302	0.0136789	0.0497342	-3.284511
CNRIP1	-1.028279	9.2911085	-2.814613	0.0136976	0.0497783	-3.285801
PEX5L	-1.122184	6.9736669	-2.812377	0.0137582	0.0498923	-3.289988
SDR16C5	-1.237167	4.5889862	-2.8118	0.0137739	0.0499314	-3.291068
