# Supplementary material for: Identification and Experimental Validation of Marker Genes between Diabetes and Alzheimer's Disease
Source: Oxid Med Cell Longev. 2022 Aug 12;2022:8122532. doi: 10.1155/2022/8122532 (PMC9391608; doi:10.1155/2022/8122532)
Supplement: Supplementary 3 — Supplementary file 2: the DEGs identified in patients with AD compared with controls (GSE122063; a total of 788 DEGs; including 475 downregulated genes and 313 upregulated genes). [file 8122532.f3.pdf]

Gene	logFC	AveExpr	t	P.Value	adj.P.Val	B
SPP1	2.0520812	12.234875	9.1888693	2.08E-12	5.39E-08	17.78305
LOC283737	-2.151132	5.2802963	-9.051435	3.36E-12	5.39E-08	17.33423
SPON2	-1.281271	8.7510405	-8.35483	3.99E-11	4.27E-07	15.026724
FAM5B	-1.212189	8.2898071	-8.162729	7.95E-11	6.37E-07	14.381805
RBM3	-1.915815	6.8847866	-7.693333	4.32E-10	1.38E-06	12.79325
TARBP1	-1.267118	8.2237624	-7.592901	6.22E-10	1.57E-06	12.451378
BACE2	1.2418388	8.6939343	7.58651	6.37E-10	1.57E-06	12.429604
ANLN	1.4532455	8.0662264	7.3870418	1.31E-09	2.81E-06	11.748896
C17orf108	-1.383909	11.747311	-7.082557	3.98E-09	7.06E-06	10.706566
LOC728052	-1.059815	9.9400615	-7.058733	4.34E-09	7.06E-06	10.62489
LOC1005071	-1.006503	8.6626823	-7.054599	4.40E-09	7.06E-06	10.610719
MYL5	-1.362834	7.8931358	-6.88054	8.30E-09	1.16E-05	10.013668
EFHB	-1.294824	4.5466947	-6.857754	9.02E-09	1.21E-05	9.9354832
LOC644189	-1.261264	8.1110707	-6.754465	1.31E-08	1.56E-05	9.581027
C4A	1.5420636	6.0352392	6.7451284	1.36E-08	1.56E-05	9.5489868
PPP1R32	-1.238324	4.0141614	-6.735259	1.41E-08	1.56E-05	9.5151174
XLOC_00347	-1.977003	7.2578762	-6.725633	1.46E-08	1.56E-05	9.4820856
XLOC_0006C	1.3998463	9.087029	6.6597342	1.86E-08	1.86E-05	9.2559549
CYP1B1-AS1	-1.090194	5.3398124	-6.64335	1.97E-08	1.87E-05	9.1997383
CCT6B	-1.887925	5.3678913	-6.642218	1.98E-08	1.87E-05	9.1958551
LOC1002893	-1.175403	6.2172455	-6.601251	2.30E-08	1.94E-05	9.055307
XLOC_00695	-2.177677	5.3757124	-6.577671	2.50E-08	1.96E-05	8.9744193
NOXA1	-1.05421	9.3125655	-6.508104	3.22E-08	2.15E-05	8.7358415
LOC1005073	-1.021142	7.6130842	-6.492756	3.41E-08	2.20E-05	8.6832187
XLOC_12_01	-1.037756	9.2800888	-6.481731	3.55E-08	2.22E-05	8.6454245
ARMCX3-AS1	1.1554125	5.5947789	6.4777289	3.60E-08	2.22E-05	8.6317041
MID1IP1	1.0737996	12.193662	6.4674797	3.74E-08	2.26E-05	8.5965712
FCGBP	2.2374355	8.0675388	6.4468653	4.03E-08	2.27E-05	8.5259165
XLOC_00093	-1.192874	5.0377069	-6.446192	4.04E-08	2.27E-05	8.5236082
CARTPT	-2.8456	8.9455249	-6.421853	4.41E-08	2.36E-05	8.4402042
BOK	1.0721068	8.2867296	6.405742	4.68E-08	2.42E-05	8.3850059
TCL1B	1.2930114	5.9721604	6.3752833	5.22E-08	2.54E-05	8.2806731
MILR1	1.1921958	6.345716	6.309098	6.64E-08	3.04E-05	8.0540811
FAM167B	1.7002702	7.3508721	6.3026704	6.80E-08	3.04E-05	8.0320847
XLOC_01428	-1.192598	7.2060016	-6.292712	7.05E-08	3.07E-05	7.9980094
CIRBP	-1.17205	9.5415149	-6.29131	7.08E-08	3.07E-05	7.9932112
LOC1005074	-1.005677	6.5996217	-6.220936	9.14E-08	3.86E-05	7.7525387
LINC00473	-1.938152	5.786114	-6.215019	9.34E-08	3.89E-05	7.7323166
XLOC_12_01	1.3180072	6.5362093	6.1891957	1.03E-07	4.17E-05	7.6440718
FSD2	1.2286465	8.7074876	6.1472895	1.19E-07	4.56E-05	7.5009467
GOLGA6C	-1.051168	9.7316425	-6.13295	1.26E-07	4.71E-05	7.4519971
MAFF	1.5950662	8.0157257	6.1250452	1.29E-07	4.72E-05	7.4250157
KRBOX1	-1.183034	5.7018944	-6.093292	1.45E-07	5.00E-05	7.316677
LOC151760	-1.411734	3.9912947	-6.08917	1.47E-07	5.00E-05	7.3026207
ACOT4	-1.4064	6.9439699	-6.087898	1.48E-07	5.00E-05	7.2982798
RASAL3	1.1463335	8.3029055	6.0786383	1.53E-07	5.06E-05	7.2667036
LOC1002876	-1.122806	7.3540227	-6.023094	1.87E-07	5.56E-05	7.0774078
ICA1	-1.057228	8.3279136	-6.020384	1.89E-07	5.56E-05	7.0681754
SNX31	1.4543877	6.0207388	5.9949274	2.07E-07	5.98E-05	6.9814983
C1QC	1.4625708	7.7846915	5.9928014	2.09E-07	5.98E-05	6.9742614

BNIPL	1.0739637	3.263232	5.9873003	2.13E-07	5.99E-05	6.9555376
C4B	1.2196102	10.517026	5.9550021	2.39E-07	6.50E-05	6.8456524
FRMPD2	-2.399961	6.5630089	-5.951437	2.42E-07	6.52E-05	6.8335271
RNF165	-1.407522	4.6376861	-5.947222	2.46E-07	6.52E-05	6.8191956
C5AR1	1.5153221	4.8760543	5.9299042	2.62E-07	6.89E-05	6.7603212
CAMKK2	-1.164115	9.3484168	-5.918424	2.73E-07	7.06E-05	6.7213077
SLAMF8	1.9950276	5.6618469	5.912142	2.79E-07	7.17E-05	6.6999622
CHORDC1	1.0274175	8.5148778	5.9091892	2.82E-07	7.17E-05	6.6899306
RUNX3	1.0169632	6.3568838	5.8928804	2.99E-07	7.22E-05	6.6345377
C1orf145	-1.30745	3.7420706	-5.890641	3.02E-07	7.22E-05	6.6269328
LOC1002331	1.1066349	6.6894649	5.8897794	3.03E-07	7.22E-05	6.6240079
CRH	-2.712227	6.5990026	-5.859278	3.38E-07	7.95E-05	6.5204799
MS4A14	1.192615	4.4272193	5.8471649	3.53E-07	7.97E-05	6.4793893
LOC1005061	-1.550994	4.45323	-5.782853	4.45E-07	9.31E-05	6.2614564
VGF	-2.045565	12.956187	-5.779619	4.50E-07	9.31E-05	6.250506
FLJ31104	-1.384229	4.7378151	-5.771525	4.63E-07	9.34E-05	6.2231082
SLC4A11	1.0782355	7.9121135	5.764326	4.75E-07	9.53E-05	6.1987476
SAP25	1.1906912	10.357569	5.7613396	4.80E-07	9.57E-05	6.188643
DRD2	-1.022964	7.4883805	-5.758529	4.85E-07	9.61E-05	6.1791339
STX1A	-1.059314	9.6413044	-5.738069	5.22E-07	0.0001009	6.1099347
ATOH7	-1.872099	4.7922371	-5.733576	5.31E-07	0.0001016	6.0947475
HLA-DRB4	1.1477236	7.5392234	5.7277505	5.42E-07	0.0001028	6.0750548
CDH19	1.1081346	6.5350272	5.7019051	5.94E-07	0.000109	5.9877346
XLOC_0110S	-1.692371	4.1937218	-5.70013	5.98E-07	0.000109	5.9817384
THNSL1	-1.168068	7.379729	-5.68915	6.22E-07	0.0001121	5.9446667
XLOC_0029E	-1.083876	4.7647884	-5.667255	6.73E-07	0.0001184	5.8707826
SCIN	1.5873883	6.9216565	5.666693	6.74E-07	0.0001184	5.8688856
SLC7A7	1.0773276	6.3802202	5.6658501	6.76E-07	0.0001184	5.8660423
TGFBI	1.2366419	7.8831419	5.6568403	6.99E-07	0.0001209	5.8356556
LOC100131C	1.0173674	5.2584821	5.6501166	7.16E-07	0.0001227	5.8129849
GPM6A	-1.261596	12.304789	-5.632948	7.61E-07	0.0001271	5.7551204
VSIG4	1.6085064	9.977252	5.6219608	7.91E-07	0.0001309	5.7181077
XLOC_01054	-1.479219	4.3958168	-5.616337	8.07E-07	0.0001328	5.6991696
SCG3	-1.121855	9.3885977	-5.609877	8.26E-07	0.0001352	5.677418
XLOC_0019E	1.0961373	10.90311	5.6012381	8.52E-07	0.0001352	5.6483382
C6orf221	-1.197906	4.3793481	-5.600446	8.55E-07	0.0001352	5.6456707
SERPINA3	1.8211709	8.0637955	5.6003737	8.55E-07	0.0001352	5.6454288
NECAB2	-1.350431	8.5799029	-5.596009	8.68E-07	0.0001359	5.6307404
C14orf79	-1.258989	4.5038181	-5.565969	9.67E-07	0.0001442	5.5297125
LILRB1	1.2485929	6.9185951	5.5554597	1.00E-06	0.0001477	5.4943968
PNMA5	-1.187454	11.209903	-5.54848	1.03E-06	0.00015	5.470949
NEAT1	1.2409596	8.7616361	5.5351858	1.08E-06	0.0001552	5.4263052
CTAG1A	-1.478562	4.4885802	-5.523643	1.12E-06	0.0001584	5.3875614
STAB1	1.1341773	6.740128	5.5202825	1.14E-06	0.0001586	5.3762852
XLOC_0048E	-1.369848	4.4568096	-5.515295	1.16E-06	0.0001599	5.3595514
LY96	1.1197892	7.5028487	5.4824444	1.30E-06	0.0001735	5.2494217
CCDC102A	1.1536463	6.3423271	5.4819993	1.30E-06	0.0001735	5.2479308
LOC1005089	1.1822323	5.7937744	5.4750285	1.34E-06	0.0001771	5.2245805
KIAA1644	-1.021412	5.8882348	-5.472286	1.35E-06	0.0001779	5.2153943
XLOC_12_0C	1.0787913	4.1543824	5.4674003	1.37E-06	0.0001779	5.1990361
CD163	2.0765168	9.3304475	5.4639203	1.39E-06	0.0001791	5.1873852

HIST1H2AH	1.4333316	10.21649	5.4610565	1.40E-06	0.0001801	5.1777988
LINC00467	-1.066243	6.0361215	-5.451142	1.45E-06	0.0001833	5.1446178
LOC285540	1.0114533	6.4057591	5.4382509	1.52E-06	0.0001886	5.1014985
GSTZ1	-1.063693	6.143053	-5.428027	1.58E-06	0.0001935	5.0673177
UNC80	-1.043148	7.2941191	-5.411599	1.67E-06	0.0001989	5.0124234
SCLT1	-1.110048	6.6090879	-5.410719	1.68E-06	0.0001989	5.0094842
HES5	-1.334195	8.7722659	-5.407472	1.70E-06	0.0001989	4.9986394
SERPINH1	1.4467219	9.5844603	5.4058397	1.71E-06	0.0001993	4.9931887
HMHA1	1.0185613	10.595449	5.3930292	1.79E-06	0.0002065	4.950422
MIA	1.7976113	4.8726396	5.3917153	1.80E-06	0.0002065	4.946037
HIST1H2AK	1.0848656	9.5689453	5.375867	1.90E-06	0.0002122	4.8931655
COL24A1	-1.214338	6.4053711	-5.367984	1.95E-06	0.0002146	4.8668816
CAMK4	-1.264041	7.0739977	-5.356813	2.03E-06	0.0002201	4.8296492
TSPAN7	-1.14211	11.048988	-5.352162	2.07E-06	0.0002217	4.8141525
UGT8	1.090361	8.4562992	5.3485766	2.09E-06	0.0002223	4.8022109
STYK1	-1.330202	7.0308112	-5.337975	2.17E-06	0.0002285	4.766909
LOC1005075	-1.036249	8.3220613	-5.335459	2.19E-06	0.0002289	4.7585337
SNORD114-2	-1.001303	7.2068282	-5.334748	2.20E-06	0.0002289	4.7561654
MET	-2.030302	8.9951245	-5.325505	2.27E-06	0.0002346	4.7254054
RCC1	1.1481401	5.2612426	5.3250744	2.27E-06	0.0002346	4.7239743
XLOC_12_01	-1.153764	4.6835502	-5.285959	2.61E-06	0.0002577	4.5939563
VASP	1.3195571	8.2968755	5.2788819	2.68E-06	0.0002627	4.5704593
WDR86	-1.086878	7.3197023	-5.270279	2.76E-06	0.0002691	4.5419084
MLIP	-1.872747	7.531097	-5.263545	2.83E-06	0.0002714	4.5195676
MYBPH	1.5962551	6.1389776	5.2544958	2.92E-06	0.0002768	4.4895558
MYO10	1.0218247	7.4206111	5.2510466	2.95E-06	0.0002775	4.478121
FREM3	-1.81741	4.9007073	-5.246301	3.00E-06	0.00028	4.4623907
XLOC_0145C	-1.11224	7.1514077	-5.242663	3.04E-06	0.0002828	4.4503346
CHRDL2	-1.086651	4.01156	-5.238383	3.09E-06	0.000285	4.4361545
SCN3B	-1.116679	12.764888	-5.237182	3.10E-06	0.000285	4.4321768
ARMCX5	-1.050274	7.9002092	-5.233282	3.14E-06	0.0002873	4.4192576
NPTX2	-1.571051	9.0560163	-5.230033	3.18E-06	0.0002878	4.4084967
PVRL3-AS1	-1.492175	5.3102783	-5.229626	3.19E-06	0.0002878	4.4071502
SDIM1	-1.785077	7.2590092	-5.217935	3.32E-06	0.0002973	4.3684475
CCR5	1.1507951	6.8788268	5.2123084	3.39E-06	0.0003007	4.3498314
PRSS35	-1.495114	5.0109312	-5.196931	3.57E-06	0.0003105	4.2989783
LOC1005069	1.3086153	6.0941008	5.1893441	3.67E-06	0.0003126	4.2739031
C17orf102	-1.81322	6.8426698	-5.189153	3.67E-06	0.0003126	4.2732727
INHA	-1.076813	4.259298	-5.186889	3.70E-06	0.0003126	4.2657912
LPIN3	1.3396978	8.5250154	5.1867614	3.70E-06	0.0003126	4.2653694
EVI2B	1.1391221	6.4310121	5.1849781	3.73E-06	0.0003137	4.2594778
LOC440040	-1.381835	6.9587362	-5.172099	3.90E-06	0.0003223	4.216946
XLOC_00439	-1.024565	3.7061021	-5.156446	4.12E-06	0.0003371	4.165294
MYOT	1.0976481	5.7022881	5.1541092	4.15E-06	0.0003381	4.1575854
XLOC_12_0C	-1.82436	8.2448799	-5.142135	4.33E-06	0.0003474	4.1181084
GDF15	1.2308932	7.5880671	5.1420864	4.33E-06	0.0003474	4.1179473
LOC285954	-1.212534	4.8576895	-5.140226	4.36E-06	0.0003488	4.1118154
LOC729264	-1.499979	5.4090437	-5.135825	4.43E-06	0.0003498	4.0973136
DOPEY1	-1.034107	7.5997148	-5.133129	4.47E-06	0.0003514	4.0884324
LOC100653C	1.1166815	5.4893253	5.1315936	4.49E-06	0.0003524	4.083375
WNT10B	-1.19347	8.1457008	-5.130481	4.51E-06	0.0003528	4.0797092

PLK2	-1.110815	10.57441	-5.129239	4.53E-06	0.0003528	4.0756194
SPRYD7	-1.008971	8.1955225	-5.124923	4.60E-06	0.0003564	4.0614083
PAMR1	-1.07798	9.3292919	-5.123064	4.63E-06	0.000357	4.0552855
HMOX1	1.073439	9.3773625	5.1230492	4.63E-06	0.000357	4.0552374
BFSP1	-1.210536	6.8691304	-5.12064	4.67E-06	0.0003575	4.0473064
AQP1	1.0401721	8.1653084	5.1124886	4.80E-06	0.0003643	4.0204792
ZBBX	-1.43564	5.4264967	-5.102056	4.98E-06	0.0003735	3.9861627
STAT4	-1.405588	7.2014089	-5.100516	5.01E-06	0.0003746	3.9810988
LOC283484	-1.422144	5.8439509	-5.094263	5.12E-06	0.0003799	3.9605413
XLOC_12_00	-1.181305	3.3909043	-5.088417	5.23E-06	0.0003828	3.9413323
XLOC_01253	-1.829865	7.9847824	-5.08049	5.37E-06	0.0003926	3.9152914
FCGR2C	1.1441608	4.9696353	5.0679466	5.61E-06	0.0004056	3.8741104
XLOC_00598	-1.416597	4.1043764	-5.062356	5.73E-06	0.0004089	3.8557667
XLOC_00654	-1.387539	5.156051	-5.056717	5.84E-06	0.0004137	3.8372707
TBL1Y	1.0082802	7.0887556	5.0511578	5.95E-06	0.0004179	3.8190407
LOC1005061	-1.155799	7.3235903	-5.048712	6.00E-06	0.0004187	3.811023
CBX3P2	-1.264216	3.9988824	-5.046909	6.04E-06	0.0004188	3.8051142
CD68	1.0443667	7.1912836	5.0467734	6.05E-06	0.0004188	3.8046685
RHOJ	1.0051562	7.9545081	5.0460351	6.06E-06	0.000419	3.8022485
XLOC_00473	1.3510348	2.0816592	5.035545	6.29E-06	0.000429	3.7678783
CAPS	1.0128787	6.9815493	5.0298583	6.41E-06	0.0004334	3.7492552
CARD6	1.0969519	6.9525161	5.0279495	6.46E-06	0.000435	3.7430055
PPEF1	-2.30031	5.3405558	-5.020528	6.62E-06	0.0004417	3.7187139
TASP1	-1.024831	6.2009094	-5.019299	6.65E-06	0.0004427	3.714691
SLC22A9	-1.341049	5.6980846	-5.018251	6.68E-06	0.0004434	3.711263
EPHB4	1.086754	6.8955762	5.0151367	6.75E-06	0.0004448	3.7010743
KCNF1	-1.009617	7.9474919	-5.007223	6.94E-06	0.0004514	3.6751917
RXFP4	1.0463395	8.380767	4.9933034	7.28E-06	0.0004573	3.6296996
HSD17B6	-1.193762	8.2623901	-4.988234	7.41E-06	0.0004583	3.6131418
SLC7A4	-1.430355	6.2810748	-4.97306	7.81E-06	0.0004756	3.5636089
GOLT1A	-1.499623	3.6501683	-4.960869	8.15E-06	0.0004881	3.5238499
CHRM1	-1.125442	12.47579	-4.959667	8.19E-06	0.0004881	3.5199321
XLOC_00777	1.1974159	5.4515417	4.9584011	8.22E-06	0.0004883	3.5158055
XLOC_00296	-1.060642	4.5794226	-4.949362	8.48E-06	0.0004976	3.4863508
LOC643783	-1.034269	4.3122784	-4.942171	8.70E-06	0.0005027	3.4629303
CHRM4	1.7497907	9.0637407	4.9349076	8.92E-06	0.0005091	3.4392856
SNAR-B2	1.0277424	12.053807	4.9304715	9.06E-06	0.0005151	3.4248499
C7orf52	-1.586984	3.7068371	-4.906257	9.85E-06	0.0005445	3.3461275
TNFRSF12A	1.2253037	7.3445276	4.9014831	1.00E-05	0.00055	3.3306241
IL18R1	1.07743	4.7678261	4.8985384	1.01E-05	0.0005523	3.3210629
XLOC_12_01	-1.146174	11.308386	-4.880657	1.08E-05	0.0005734	3.2630432
SST	-2.353122	5.9124708	-4.871334	1.11E-05	0.0005835	3.2328224
SIDT1	-1.480124	7.1353035	-4.865807	1.13E-05	0.0005908	3.2149144
LOC646482	-1.139449	4.580348	-4.86502	1.14E-05	0.0005915	3.212365
RGS7	-1.213933	7.6352628	-4.862391	1.15E-05	0.000594	3.2038507
HAPLN1	-1.154256	6.1144937	-4.847489	1.21E-05	0.0006144	3.1556151
LPAR1	1.0527562	9.6337187	4.8444038	1.22E-05	0.000617	3.1456335
LOC100288C	-1.051894	9.2344728	-4.841288	1.23E-05	0.0006208	3.1355554
PNOC	-1.401632	7.7326903	-4.832659	1.27E-05	0.0006335	3.1076609
LY6H	-1.084091	9.3727028	-4.829914	1.28E-05	0.000635	3.098791
FCGR2A	1.0082021	7.4590807	4.8287128	1.29E-05	0.0006363	3.0949095

C1QB	1.2359542	10.428044	4.8203699	1.32E-05	0.0006425	3.0679627
PEX6	1.2132043	8.5078194	4.8195997	1.33E-05	0.0006425	3.0654756
PRR22	-1.102497	4.9692049	-4.81932	1.33E-05	0.0006425	3.0645722
PAPL	-1.358552	11.262435	-4.805297	1.40E-05	0.0006598	3.01932
LOC1005055	-1.240477	4.3456703	-4.79847	1.43E-05	0.000667	2.9973039
FBXO16	-1.305646	7.6476386	-4.797491	1.43E-05	0.0006677	2.9941487
GPLD1	-1.026376	5.5894878	-4.796492	1.44E-05	0.0006677	2.9909278
PTPRR	-1.168337	5.9640004	-4.794676	1.45E-05	0.0006691	2.9850773
RHBDF2	1.0556381	10.247029	4.7929676	1.46E-05	0.0006709	2.9795701
GABRE	1.1277035	7.676862	4.7925284	1.46E-05	0.000671	2.9781545
C6orf154	-1.034485	9.151181	-4.791711	1.46E-05	0.0006719	2.9755206
CD37	1.0100462	5.1145642	4.7818004	1.51E-05	0.0006873	2.9435983
TEKT3	-1.048547	3.7510101	-4.777371	1.54E-05	0.0006942	2.9293371
XLOC_01396	-1.289217	5.6159052	-4.759129	1.64E-05	0.0007207	2.8706626
CHRM3	-1.019951	7.1484627	-4.753952	1.66E-05	0.0007264	2.8540251
XLOC_00972	-1.036368	3.4331983	-4.750711	1.68E-05	0.0007315	2.8436103
MEIS3	-1.041733	5.2499358	-4.749502	1.69E-05	0.0007326	2.8397267
GPC5	-1.06792	8.3608121	-4.746843	1.71E-05	0.0007344	2.8311867
HSPA1A	1.3560351	12.637254	4.7380791	1.76E-05	0.0007506	2.8030528
CACNG3	-1.522139	7.6767435	-4.733179	1.79E-05	0.0007573	2.7873312
GAS7	-1.478593	9.8776716	-4.732596	1.79E-05	0.0007578	2.7854609
XLOC_00777	1.2083339	6.1689526	4.7302143	1.81E-05	0.000761	2.7778201
C20orf195	1.0891338	4.9457481	4.7251937	1.84E-05	0.0007691	2.7617206
CHGB	-1.073797	9.1468022	-4.723349	1.85E-05	0.000772	2.7558069
XLOC_00771	-1.164966	4.5725469	-4.716442	1.89E-05	0.0007843	2.7336699
LRTM2	-1.598697	6.7079675	-4.713751	1.91E-05	0.00079	2.7250499
ZIM2	-1.028937	6.9837791	-4.710141	1.93E-05	0.0007952	2.713488
XLOC_00124	1.3935769	7.3320152	4.7068439	1.96E-05	0.0008022	2.7029317
GRIA4	-1.007685	9.3891128	-4.697814	2.02E-05	0.0008241	2.6740319
CRYM	-2.033591	12.380994	-4.692887	2.05E-05	0.0008328	2.6582716
DLGAP2	-1.627	7.5697401	-4.691667	2.06E-05	0.0008339	2.6543706
IL3RA	1.1579454	7.570428	4.6914057	2.06E-05	0.0008339	2.6535361
DNAH2	-1.163496	7.1465105	-4.689664	2.07E-05	0.0008341	2.6479658
C6orf118	1.4798797	6.2416693	4.6896264	2.07E-05	0.0008341	2.6478472
C5orf55	-1.097581	6.1047442	-4.689482	2.08E-05	0.0008341	2.647385
XLOC_00598	1.1675991	4.5408374	4.683664	2.12E-05	0.0008453	2.6287887
SYCE1	-1.072163	5.0640683	-4.682795	2.12E-05	0.0008457	2.6260111
HIGD1B	1.1699165	10.192456	4.6825191	2.13E-05	0.0008457	2.6251303
XLOC_01054	-1.111135	3.8662487	-4.681174	2.13E-05	0.0008458	2.6208332
SSR4P1	-1.26158	5.0844053	-4.679478	2.15E-05	0.0008471	2.6154126
FIBIN	-1.244463	6.091688	-4.678622	2.15E-05	0.0008485	2.6126791
LOC1005062	-1.606697	6.2773769	-4.677806	2.16E-05	0.0008488	2.6100723
GEM	1.0495953	7.420879	4.6763438	2.17E-05	0.000852	2.6054029
TNFRSF10B	1.0117514	7.8028206	4.674553	2.18E-05	0.0008552	2.599684
ADHFE1	-1.004849	7.6566449	-4.662354	2.28E-05	0.000879	2.5607482
XLOC_00294	-1.104288	4.050031	-4.656606	2.32E-05	0.0008907	2.5424136
IL33	-1.25208	7.7796991	-4.645853	2.41E-05	0.0009102	2.5081422
LOC1005062	-1.550878	4.9557236	-4.645135	2.41E-05	0.0009102	2.505854
PMAIP1	1.0656737	6.3255491	4.6427213	2.43E-05	0.000915	2.4981662
DGKI	-1.147995	5.9692548	-4.63968	2.46E-05	0.0009169	2.4884793
CSDA	1.5063544	8.3570171	4.6315674	2.53E-05	0.0009373	2.4626563

LOC1005067	-1.223221	5.5502407	-4.627878	2.56E-05	0.0009458	2.4509179
LOC1001895	-1.583693	5.1189342	-4.62161	2.61E-05	0.0009595	2.4309834
CHRNB2	-1.025239	10.651513	-4.605121	2.77E-05	0.001001	2.3785912
EVI2A	1.0263601	8.4577489	4.602109	2.79E-05	0.001009	2.3690304
TNFRSF10D	1.5343025	7.3305494	4.5990185	2.82E-05	0.0010111	2.3592215
GBP2	1.0346776	6.0390708	4.5970278	2.84E-05	0.0010111	2.3529045
KCNB2	-1.459657	6.9877954	-4.596561	2.85E-05	0.0010111	2.3514246
CCR1	1.064346	7.4176151	4.5924187	2.89E-05	0.0010197	2.3382829
S100A4	1.1241362	7.0563341	4.5902051	2.91E-05	0.0010251	2.3312628
CXCR4	1.0175931	9.1245183	4.5832768	2.98E-05	0.0010357	2.3092986
IFI30	1.0174609	8.656211	4.5821244	2.99E-05	0.0010357	2.3056466
ROBO2	-1.021522	5.24659	-4.580981	3.00E-05	0.0010361	2.3020228
ST7-AS1	-1.24171	6.0521662	-4.576102	3.05E-05	0.0010422	2.2865679
TLR2	1.1849882	7.6506285	4.568722	3.13E-05	0.0010539	2.2631998
RFPL1-AS1	-1.204467	9.9050573	-4.567584	3.14E-05	0.0010543	2.259599
HOXC11	1.2669336	2.441525	4.5637783	3.18E-05	0.0010583	2.2475551
NKX6-2	1.040396	10.731895	4.5619917	3.20E-05	0.0010633	2.2419028
C20orf141	1.2883409	12.642772	4.5614509	3.21E-05	0.0010633	2.2401922
PTPN3	-1.599868	5.9687698	-4.540831	3.44E-05	0.0011192	2.1750248
CDNF	-1.110722	3.8954918	-4.537902	3.47E-05	0.0011261	2.1657762
SVOP	-1.47895	10.71006	-4.537814	3.47E-05	0.0011261	2.1655002
LOC145694	-1.066745	4.5218854	-4.536009	3.49E-05	0.0011307	2.1598005
LOC1001309	-1.024537	5.9124526	-4.534206	3.51E-05	0.0011353	2.1541104
CNTNAP2	-1.206641	9.449914	-4.528873	3.58E-05	0.0011524	2.1372857
SLC16A3	1.0058102	9.6058975	4.5174122	3.72E-05	0.0011875	2.1011551
WDR54	-1.168848	7.8691288	-4.517016	3.72E-05	0.0011875	2.0999064
LOC1005099	-1.472527	8.6102834	-4.515493	3.74E-05	0.0011924	2.095107
LOC401442	-1.649905	5.7546473	-4.506901	3.85E-05	0.0012154	2.0680506
XLOC_00692	-1.003597	4.2817537	-4.503683	3.90E-05	0.0012226	2.0579208
ARPP21	-1.325241	9.2078014	-4.499404	3.95E-05	0.0012277	2.0444567
FLT1	1.0882356	7.8500719	4.494242	4.02E-05	0.0012353	2.0282219
LOC375295	-1.248141	5.209665	-4.48705	4.12E-05	0.0012549	2.0056137
SLC22A6	-1.271153	3.7231616	-4.481352	4.20E-05	0.0012752	1.9877149
LY86-AS1	-1.489552	6.3731883	-4.479332	4.23E-05	0.0012795	1.9813696
CCL2	1.5474757	7.9321988	4.479009	4.23E-05	0.0012795	1.9803565
LOC1001315	1.0855847	5.9374903	4.4785065	4.24E-05	0.0012795	1.9787787
CPB1	-1.012962	3.4467958	-4.478376	4.24E-05	0.0012795	1.9783675
CCDC113	-1.059957	6.3844819	-4.476175	4.27E-05	0.0012857	1.9714575
RANBP17	-1.201979	5.4450104	-4.475868	4.28E-05	0.0012859	1.9704962
XLOC_12_0C	-1.367045	4.6361727	-4.474631	4.30E-05	0.00129	1.9666124
XLOC_00169	-1.011126	5.3013361	-4.464368	4.45E-05	0.0013253	1.9344134
PKP3	-1.275524	5.7265655	-4.463132	4.46E-05	0.0013296	1.9305382
XLOC_12_0C	-1.395247	11.248048	-4.462171	4.48E-05	0.0013303	1.9275251
LOC646241	-1.373418	4.488135	-4.455485	4.58E-05	0.0013479	1.9065727
FAM19A1	-1.063669	6.9558433	-4.454525	4.60E-05	0.0013498	1.9035632
LOC644450	1.1568945	8.8897912	4.4505385	4.66E-05	0.0013641	1.8910765
XLOC_0084C	-1.318325	6.2445565	-4.448405	4.69E-05	0.0013695	1.8843966
HTR2A	-1.23825	8.9212687	-4.44799	4.70E-05	0.0013696	1.8830963
KDM4D	-1.287966	4.3326252	-4.445938	4.73E-05	0.0013728	1.8766734
TAAR5	-1.361727	10.337159	-4.443437	4.77E-05	0.00138	1.868845
ANXA8L2	-1.237618	5.8957727	-4.442897	4.78E-05	0.0013802	1.8671542

MAEL	-1.07199	4.8800354	-4.425414	5.07E-05	0.0014456	1.8124876
XLOC_00004	-1.023872	4.5894105	-4.423148	5.10E-05	0.0014514	1.8054072
SNAR-H	1.0181466	11.272567	4.4107286	5.32E-05	0.0014902	1.7666378
WIF1	-1.246326	10.342177	-4.410075	5.33E-05	0.0014911	1.7646
SYT16	-1.400463	5.1736961	-4.407544	5.38E-05	0.0015025	1.756704
XLOC_01097	-1.259913	3.4568859	-4.405068	5.42E-05	0.001511	1.7489822
ATL1	-1.162163	9.8379158	-4.397447	5.56E-05	0.0015357	1.7252268
PRMT8	-1.693717	5.8499886	-4.386934	5.76E-05	0.001572	1.6924889
XLOC_00595	1.2778197	3.8959275	4.3867555	5.76E-05	0.001572	1.6919316
WEE2	-1.016602	2.8485196	-4.383467	5.83E-05	0.0015826	1.6816985
ZNF214	-1.160931	4.4571774	-4.373582	6.02E-05	0.0016205	1.6509551
SFTPD	-1.281393	5.2655641	-4.371976	6.05E-05	0.0016279	1.6459618
XLOC_00436	1.0688886	4.6974293	4.3706833	6.08E-05	0.0016314	1.6419449
PDLIM4	1.1521512	6.2335278	4.37032	6.09E-05	0.0016314	1.6408159
PLD3	-1.025399	9.4695585	-4.368059	6.13E-05	0.0016369	1.6337898
PPM1J	-1.151596	6.4332242	-4.36743	6.15E-05	0.001639	1.631836
DIO3OS	1.0144785	5.8779747	4.353025	6.45E-05	0.0017095	1.5871166
REP15	2.0985467	8.3778885	4.3516441	6.48E-05	0.0017131	1.582833
11-Mar	-1.553091	4.918436	-4.350486	6.50E-05	0.0017183	1.5792403
ARHGAP36	-1.135946	5.5493866	-4.350064	6.51E-05	0.0017193	1.5779307
SLC26A4	-1.307917	5.9647976	-4.346794	6.58E-05	0.0017317	1.5677915
ZWILCH	-1.289818	4.7302321	-4.336664	6.81E-05	0.0017737	1.5364038
AEBP1	1.0909876	8.9137765	4.3329072	6.89E-05	0.0017737	1.5247699
AP3B2	-1.053992	8.3224689	-4.332614	6.90E-05	0.0017737	1.5238616
SNAR-G2	1.0256598	11.559519	4.3140587	7.34E-05	0.0018493	1.4664725
PXDNL	-1.000739	3.8516361	-4.314041	7.34E-05	0.0018493	1.4664177
GNG2	-1.396615	8.5915469	-4.312525	7.38E-05	0.0018528	1.4617344
SRGN	1.0624267	9.4642116	4.3047239	7.57E-05	0.001885	1.4376423
XLOC_12_0C	-1.088948	4.0546311	-4.297611	7.75E-05	0.0019062	1.415694
LOC286002	-1.64993	6.0168446	-4.293131	7.87E-05	0.0019215	1.4018769
LOC1005064	-1.23913	5.3063969	-4.287455	8.01E-05	0.0019445	1.3843806
SNAR-F	1.1351689	10.872815	4.2784614	8.26E-05	0.0019811	1.356681
GP9	1.059976	6.821875	4.2770002	8.30E-05	0.0019872	1.352183
PCDH11Y	-1.071675	5.0509632	-4.273185	8.40E-05	0.0019981	1.3404434
CAMK1G	-1.194197	9.3482735	-4.268663	8.53E-05	0.0020172	1.3265322
ANKRD55	-1.046222	4.6511733	-4.257306	8.85E-05	0.0020682	1.2916273
PRPH2	-1.235051	5.7716016	-4.256411	8.88E-05	0.0020728	1.2888761
XLOC_00248	-1.480613	10.03366	-4.253842	8.95E-05	0.0020857	1.2809893
COPG2IT1	-1.041503	13.085368	-4.249021	9.10E-05	0.0021059	1.2661882
DUS2L	-1.007904	4.473665	-4.247233	9.15E-05	0.0021103	1.2607034
NRSN1	-1.146417	5.9535885	-4.243851	9.25E-05	0.0021263	1.2503273
GJB6	-1.204352	8.9265391	-4.239658	9.38E-05	0.0021485	1.2374683
ADAMTS1	1.2223721	7.1615879	4.2299539	9.69E-05	0.0022022	1.2077347
CBLN4	-1.648464	9.1090973	-4.221009	9.98E-05	0.0022519	1.1803544
UNC13A	-1.419044	7.2554889	-4.214605	0.0001019	0.0022712	1.1607676
NGEF	-1.012587	12.215841	-4.213152	0.0001024	0.0022766	1.1563246
BCAS1	1.0873049	10.845374	4.2104077	0.0001033	0.0022905	1.1479368
C3orf71	-1.099976	4.7027931	-4.208601	0.0001039	0.002297	1.1424162
BDNF	-1.328633	4.4582594	-4.205813	0.0001049	0.0023086	1.1338996
MOXD1	-1.007341	9.4656007	-4.204621	0.0001053	0.0023146	1.1302573
NPTXR	-1.18056	7.6112501	-4.202459	0.000106	0.0023246	1.1236566

CCKBR	-1.33096	7.3190504	-4.19838	0.0001075	0.0023415	1.1112051
CYP4X1	-1.147327	7.779546	-4.182192	0.0001133	0.0024326	1.0618439
INSM1	-1.249613	4.8963683	-4.181419	0.0001136	0.0024346	1.059487
FOXJ1	1.2229872	6.060976	4.1734741	0.0001166	0.0024781	1.0352975
MIR7-3HG	-1.108402	7.0091539	-4.168266	0.0001186	0.0025041	1.0194519
GDA	-1.534396	8.5706751	-4.166259	0.0001194	0.0025123	1.0133486
TOX	-1.118211	6.6148598	-4.164948	0.0001199	0.0025198	1.0093616
XLOC_00008	-1.140415	3.2208719	-4.159809	0.0001219	0.0025408	0.9937399
SNORA73A	1.2664819	7.878167	4.15598	0.0001234	0.0025661	0.9821075
DOC2A	-1.273306	7.1703346	-4.155669	0.0001236	0.002567	0.9811636
GNG3	-1.299234	9.3795078	-4.154689	0.000124	0.0025736	0.9781851
ODZ3	-1.120791	4.6111594	-4.138748	0.0001306	0.002678	0.929819
LYVE1	1.1678168	6.3031901	4.1366635	0.0001315	0.0026866	0.9234994
PRRT1	-1.190911	10.539929	-4.129074	0.0001348	0.0027333	0.9005084
C15orf27	-1.022368	7.4536027	-4.11725	0.0001401	0.0028128	0.8647281
RPL13AP17	-1.109883	12.309509	-4.115416	0.0001409	0.0028244	0.8591832
Q958C4	-1.132795	5.0273776	-4.114811	0.0001412	0.002825	0.8573537
FLJ25917	-1.310785	4.528529	-4.113714	0.0001417	0.0028294	0.8540376
XLOC_00554	-1.010372	2.9225928	-4.109563	0.0001436	0.0028607	0.8414963
XLOC_12_0C	-1.3146	5.0712054	-4.108817	0.0001439	0.0028623	0.8392416
C21orf62	1.0767348	4.5664853	4.1057764	0.0001454	0.0028796	0.8300579
SLC30A3	-1.28808	10.8309	-4.101113	0.0001476	0.002903	0.8159786
LOC1001299	-1.200316	5.03374	-4.099873	0.0001482	0.002903	0.8122364
VENTXP1	1.099304	2.1410997	4.0986157	0.0001488	0.0029053	0.808443
CLSTN3	-1.094688	9.771355	-4.098182	0.000149	0.0029053	0.8071359
FBXW9	-1.065991	5.202116	-4.097741	0.0001492	0.0029077	0.8058027
PGM2L1	-1.078475	11.27233	-4.094296	0.0001509	0.0029333	0.7954122
SCARNA11	1.4230919	5.1988451	4.0906564	0.0001527	0.0029627	0.7844391
HSPA1B	1.2375829	10.654879	4.0879484	0.000154	0.0029758	0.7762773
C6orf222	-1.219334	3.657152	-4.086218	0.0001549	0.0029759	0.7710626
FLJ32063	-1.851224	6.3665123	-4.084042	0.000156	0.0029817	0.7645089
SLC10A4	-1.445437	5.7036822	-4.084016	0.000156	0.0029817	0.7644316
XLOC_00575	-1.117403	7.1965131	-4.081713	0.0001572	0.0029862	0.7574933
BTN3A1	1.1312821	6.7223616	4.0793065	0.0001584	0.0030011	0.750249
XLOC_01003	1.0493861	5.636555	4.078113	0.000159	0.0030054	0.7466565
CHAF1B	-1.03323	7.3207152	-4.077874	0.0001591	0.0030054	0.7459379
NECAB1	-1.676052	8.6060794	-4.070931	0.0001628	0.0030512	0.72505
ENC1	-1.38147	9.0940908	-4.06942	0.0001636	0.0030644	0.7205049
SYN2	-1.054822	11.431257	-4.065316	0.0001657	0.0030945	0.7081676
SRGAP3	-1.081148	7.7907971	-4.051922	0.0001731	0.0031961	0.6679496
IL1RL1	1.9376313	4.1044515	4.0493974	0.0001745	0.0032119	0.6603754
LOC1006528	-1.006917	6.4792309	-4.049278	0.0001746	0.0032119	0.6600177
BEX1	-1.289502	13.712982	-4.045598	0.0001767	0.0032341	0.6489813
BEX2	-1.144769	13.523598	-4.041307	0.0001791	0.0032662	0.6361218
XLOC_00451	1.0034454	7.7518122	4.0311854	0.0001851	0.0033423	0.6058106
XLOC_12_0C	-1.208102	6.2091471	-4.028729	0.0001865	0.0033613	0.59846
XLOC_12_0C	-1.041086	5.5428055	-4.019915	0.0001919	0.0034178	0.5721046
OLFM4	-1.351778	5.6662893	-4.016953	0.0001937	0.003441	0.563255
EGFL6	-1.459384	4.9038208	-4.013908	0.0001957	0.0034615	0.5541592
HCG4	-1.133854	4.6384994	-4.010368	0.0001979	0.0034876	0.5435909
XLOC_00506	-1.782777	5.3779492	-4.009091	0.0001987	0.0034905	0.5397794

PPP1R14C	-1.339242	5.5967199	-4.008667	0.000199	0.0034933	0.5385122
SLCO1C1	-1.169349	7.8321475	-4.006458	0.0002004	0.0035016	0.5319207
XLOC_01148	-1.145155	8.3005643	-4.005547	0.000201	0.0035074	0.5292029
TMEM233	-1.053632	6.0684685	-4.005136	0.0002013	0.0035102	0.5279777
XLOC_00121	1.0209626	5.2259964	4.0036125	0.0002022	0.003517	0.5234332
CACNG1	-1.110142	4.049691	-4.001806	0.0002034	0.0035292	0.5180475
AKAP5	-1.126968	6.1077976	-3.99957	0.0002049	0.0035434	0.5113795
LOC100129C	1.2921974	5.337675	3.9971763	0.0002065	0.0035609	0.5042453
XLOC_0077C	-1.027563	4.1604348	-3.98743	0.000213	0.0036261	0.4752187
PNMA6C	-1.034372	9.0185047	-3.987326	0.0002131	0.0036261	0.4749081
XLOC_0088C	1.2354037	2.2473782	3.9855865	0.0002143	0.0036387	0.4697326
XLOC_00452	-1.150646	3.252185	-3.982391	0.0002165	0.0036684	0.4602266
MS4A6A	1.1594726	5.593661	3.9756044	0.0002213	0.0037118	0.4400477
NAP1L5	-1.13534	10.480816	-3.967831	0.0002268	0.0037758	0.4169571
SPINK8	1.0043619	6.9960186	3.9660567	0.0002281	0.003789	0.4116903
ANGPT2	1.6553484	8.5219295	3.9638133	0.0002298	0.0038109	0.4050323
DACH2	-1.548307	7.507959	-3.962829	0.0002305	0.0038189	0.4021128
RGS17	-1.170378	5.2090383	-3.962206	0.000231	0.0038206	0.4002622
TRIM36	-1.200003	7.4230055	-3.955026	0.0002363	0.0038933	0.3789716
BEX5	-1.390789	10.820205	-3.954745	0.0002365	0.0038948	0.378139
SLC17A6	-1.281111	9.020945	-3.95333	0.0002376	0.0039008	0.3739461
MYT1L	-1.644724	7.7358489	-3.95138	0.0002391	0.0039109	0.3681667
LOC389023	-1.048727	8.8168513	-3.941426	0.0002468	0.0040167	0.3386972
GPRIN2	-1.132778	3.6194936	-3.939066	0.0002487	0.0040328	0.3317147
GATA2	1.090842	5.2166701	3.9344814	0.0002524	0.0040544	0.3181587
PCP4L1	-1.492325	8.4424586	-3.932827	0.0002537	0.0040613	0.3132687
PDE1A	-1.042384	6.5160656	-3.932662	0.0002538	0.0040613	0.3127799
GRP	-1.156973	5.4651268	-3.916128	0.0002676	0.0042214	0.2639696
ADCYAP1	-1.080041	5.8470823	-3.910254	0.0002726	0.00428	0.2466537
UBE2N	-1.00469	7.7055726	-3.909122	0.0002736	0.0042912	0.2433177
XLOC_00837	-1.06217	2.4164852	-3.908627	0.000274	0.0042938	0.2418621
LOC440896	-1.134954	8.1110005	-3.907174	0.0002753	0.0043074	0.2375813
XLOC_00376	1.2906304	2.6226632	3.9050286	0.0002772	0.0043288	0.2312625
SNORD1B	1.2832162	4.7641394	3.8959551	0.0002853	0.0044227	0.2045612
OR13J1	1.106288	2.6640149	3.895574	0.0002856	0.004426	0.2034404
ENPP5	-1.341753	7.4923279	-3.893288	0.0002877	0.0044527	0.1967176
SPINK2	-1.041452	4.3611225	-3.891236	0.0002896	0.0044636	0.1906863
FLJ45832	-1.017075	5.0618079	-3.885283	0.0002951	0.0045292	0.1731964
SNORA14B	1.2826537	7.0396472	3.8835037	0.0002968	0.0045483	0.1679726
NFE2	1.0607928	5.478741	3.8799776	0.0003001	0.0045885	0.1576222
LOC100132C	1.3327184	6.2061054	3.8779502	0.0003021	0.0046049	0.1516733
CREG2	-1.323773	10.419044	-3.876226	0.0003037	0.0046189	0.1466163
FRMPD4	-1.072322	8.4349551	-3.87424	0.0003056	0.0046308	0.1407901
PHYHIP	-1.082917	10.467747	-3.872417	0.0003074	0.0046488	0.1354455
XLOC_00467	1.131663	4.1789455	3.871276	0.0003085	0.0046568	0.132101
CNR1	-1.604121	8.7785812	-3.868241	0.0003115	0.0046908	0.1232058
SYT5	-1.112922	5.4189856	-3.864214	0.0003155	0.0047266	0.1114106
LOC441052	-1.354241	5.213716	-3.855484	0.0003243	0.0048143	0.085863
LOC285181	1.0212388	3.9790424	3.8544993	0.0003253	0.0048255	0.082982
LOC401022	1.1937769	4.765254	3.8470988	0.000333	0.0048985	0.0613511
OLFM3	-1.485157	6.8926658	-3.839033	0.0003416	0.0049831	0.0378

ADCY2	-1.200931	5.7359806	-3.837104	0.0003437	0.0050044	0.0321717
GAP43	-1.201734	10.681986	-3.833857	0.0003473	0.0050319	0.0227008
SYP	-1.538019	8.3668374	-3.83347	0.0003477	0.0050324	0.021572
LOC1001312	-1.057622	5.1495252	-3.831996	0.0003493	0.0050512	0.0172759
FAM75A2	-1.052187	3.654676	-3.825679	0.0003563	0.0051154	-0.001132
GAD1	-1.339002	7.9566268	-3.82539	0.0003566	0.0051159	-0.001973
CLEC2B	1.0361407	6.1877553	3.8252552	0.0003568	0.0051159	-0.002367
KCNE4	1.320147	8.1962143	3.8224953	0.0003599	0.0051468	-0.010404
GPRASP2	-1.239545	9.7543488	-3.822151	0.0003603	0.0051478	-0.011405
PCDH8	-1.632685	9.2169627	-3.820072	0.0003627	0.0051678	-0.017458
KCNV1	-1.353304	8.5189834	-3.819705	0.0003631	0.0051715	-0.018526
ENTPD3	-1.669868	7.4982779	-3.818428	0.0003646	0.0051758	-0.022242
C1QA	1.0352036	8.0630239	3.813805	0.0003699	0.0052197	-0.035689
SNORD30	1.1267815	5.5390369	3.8066025	0.0003784	0.0052997	-0.056622
LOC1005075	1.0503605	2.3759506	3.8052024	0.00038	0.0053163	-0.060689
MAGEL2	-1.245051	7.6550331	-3.80506	0.0003802	0.0053163	-0.061103
C2orf80	-1.003943	9.7277903	-3.803341	0.0003823	0.0053403	-0.066095
XLOC_00914	1.148383	2.2609819	3.7974669	0.0003894	0.0054056	-0.083143
FSTL5	-1.191816	7.0806493	-3.791796	0.0003964	0.0054684	-0.099589
SLC5A11	1.0659733	8.7497779	3.7895554	0.0003992	0.0054952	-0.106082
XLOC_00149	1.1801924	2.2256051	3.7874109	0.0004019	0.005518	-0.112295
MAL2	-1.684218	8.6759813	-3.785847	0.0004039	0.005541	-0.116827
NRXN3	-1.000538	6.9620529	-3.784546	0.0004055	0.0055573	-0.120592
CITED1	-1.180635	4.9394517	-3.778882	0.0004128	0.0056208	-0.136987
NME5	-1.138871	7.2546728	-3.777837	0.0004141	0.005629	-0.140011
XLOC_00986	-1.007052	5.8031128	-3.776522	0.0004158	0.0056396	-0.143814
XLOC_01256	1.0795492	2.7477746	3.7699009	0.0004245	0.0057251	-0.162956
RAB27B	-1.363488	6.665974	-3.767082	0.0004283	0.0057408	-0.171101
SLC4A10	-1.087016	7.9375634	-3.762468	0.0004345	0.0057904	-0.184424
LOC729506	1.1497056	6.0643381	3.7572052	0.0004417	0.0058508	-0.199608
SV2B	-1.144365	11.827483	-3.757157	0.0004418	0.0058508	-0.199747
LOC613126	-1.055322	3.7323028	-3.756667	0.0004425	0.0058574	-0.20116
RBM11	-1.003334	6.6684008	-3.747929	0.0004547	0.0059775	-0.226345
XLOC_00519	1.131182	3.6532911	3.7435414	0.000461	0.006035	-0.238978
TUBB3	-1.00946	12.283959	-3.74227	0.0004628	0.0060456	-0.242638
SYNPR	-1.433329	11.073711	-3.738287	0.0004686	0.0061	-0.254096
ANO3	-1.011676	9.3284863	-3.735514	0.0004727	0.0061405	-0.262071
TNFSF14	1.1559733	2.741175	3.7327131	0.0004768	0.0061743	-0.270122
XLOC_00493	1.0304884	4.8364917	3.7311426	0.0004792	0.0061921	-0.274635
XLOC_00153	1.2208631	2.5175249	3.7251879	0.0004881	0.0062775	-0.291736
PNMA6A	-1.278217	4.755868	-3.723297	0.000491	0.0062989	-0.297163
HLA-DRB5	1.1702681	8.5869626	3.7178361	0.0004994	0.0063616	-0.312829
SOCS3	1.7095045	6.7620494	3.714537	0.0005046	0.0064122	-0.322287
XLOC_00619	1.0507995	2.1307882	3.7034559	0.0005222	0.006574	-0.354019
XLOC_00148	1.3913278	7.2070681	3.6921971	0.0005408	0.0067154	-0.386206
XLOC_01347	1.1452927	6.2687533	3.6915406	0.0005419	0.0067238	-0.388081
LOC1005067	-1.012863	2.8075284	-3.6831	0.0005562	0.0068385	-0.412174
HSPB3	-1.461958	8.0123957	-3.679441	0.0005626	0.006903	-0.422606
LOC1001291	-1.221545	10.308264	-3.675084	0.0005702	0.0069762	-0.435022
XK	-1.128436	9.2055332	-3.672563	0.0005747	0.0070167	-0.442203
FLJ40453	1.218151	2.3690295	3.6696398	0.0005799	0.0070575	-0.450527

XLOC_00249	1.306044	6.5130439	3.6676551	0.0005835	0.0070755	-0.456175
HPRT1	-1.189121	10.919953	-3.664589	0.000589	0.0071138	-0.464899
HS6ST2	-1.090692	6.5410663	-3.661431	0.0005948	0.0071638	-0.473878
DLX5	-1.261868	6.4397755	-3.645886	0.000624	0.0073784	-0.518017
GAD2	-1.232887	6.2063962	-3.645352	0.000625	0.0073836	-0.519533
HTR3B	-1.011301	4.1231809	-3.645249	0.0006252	0.0073836	-0.519823
XLOC_00114	-1.148839	9.8499378	-3.642979	0.0006296	0.0074082	-0.52626
XLOC_00390	1.0747675	2.280043	3.6367103	0.0006419	0.007514	-0.544021
CD200	-1.398947	7.755498	-3.62953	0.0006562	0.0076266	-0.564343
CADPS	-1.048893	7.7563345	-3.626001	0.0006634	0.0076787	-0.574323
CEP41	-1.391904	7.6924439	-3.625877	0.0006636	0.0076788	-0.574674
DOCK3	-1.28533	9.9057956	-3.622426	0.0006707	0.00773	-0.584426
KIT	-1.013959	7.3973738	-3.62099	0.0006737	0.0077502	-0.588484
TMEM200A	-1.052025	6.229696	-3.610134	0.0006965	0.0079472	-0.619124
VSNL1	-1.540503	13.616134	-3.60382	0.0007101	0.0080576	-0.63692
C1orf173	-1.573314	8.3850616	-3.601919	0.0007142	0.0080865	-0.642275
XLOC_01167	1.3921919	5.7012412	3.5956223	0.0007281	0.0081917	-0.66
ZNF620	1.5049918	7.0098663	3.5946164	0.0007304	0.0082085	-0.662829
XLOC_00951	1.6772592	4.6367558	3.5920732	0.0007361	0.0082438	-0.669982
XLOC_12_01	1.3490462	2.1308415	3.5884152	0.0007444	0.0083075	-0.680264
SRRM5	1.0573031	6.7688088	3.584523	0.0007533	0.008372	-0.691199
XLOC_00556	1.0441624	3.0124696	3.5820202	0.0007591	0.0084158	-0.698226
IGLL5	1.0768185	3.791576	3.5797347	0.0007644	0.0084485	-0.704641
STMN2	-1.437263	14.061786	-3.571952	0.0007828	0.0085953	-0.726466
XLOC_01222	1.1554101	2.6597013	3.5629579	0.0008045	0.0087208	-0.751655
DYDC2	-1.411872	4.3086758	-3.56285	0.0008048	0.0087208	-0.751957
XLOC_00520	1.2499058	4.2293554	3.5604438	0.0008107	0.0087615	-0.758688
FGF12	-1.075625	9.5254851	-3.548954	0.0008396	0.0089943	-0.790799
PCDH11X	-1.013988	5.3680598	-3.54787	0.0008423	0.0090133	-0.793825
PNCK	-1.129729	6.2829251	-3.546535	0.0008458	0.0090335	-0.797551
GPR158	-1.342084	9.3260173	-3.546078	0.0008469	0.0090336	-0.798826
BACE1-AS	1.6595775	7.0616225	3.5454072	0.0008487	0.0090465	-0.800698
XLOC_00706	-1.631912	7.7562667	-3.544305	0.0008515	0.0090718	-0.803774
MLLT11	-1.186715	12.18294	-3.534376	0.0008776	0.0092633	-0.831451
XLOC_01381	-1.513629	9.294756	-3.533789	0.0008791	0.0092758	-0.833087
DLX6	-1.288941	5.9503042	-3.531912	0.0008842	0.0093135	-0.838312
FCER1A	1.0997576	3.4327892	3.5304336	0.0008881	0.0093309	-0.842427
WDR16	-1.016338	6.4433836	-3.530126	0.000889	0.0093359	-0.843283
SLC22A10	-1.382657	8.8557101	-3.529531	0.0008906	0.0093381	-0.844938
LOC375196	-1.013973	6.7307286	-3.522361	0.0009101	0.0094659	-0.86488
PIGR	1.0388592	3.0005352	3.5219736	0.0009112	0.009474	-0.865958
XLOC_00218	1.0288271	2.4256423	3.5192795	0.0009187	0.0095278	-0.873444
S100A5	1.2412199	9.9793045	3.5163802	0.0009268	0.0095892	-0.881497
XLOC_01052	1.0858645	4.3947082	3.516159	0.0009274	0.0095894	-0.882111
XLOC_01372	1.1695772	2.2296257	3.5090632	0.0009475	0.0097348	-0.901802
LOC1005062	1.1244986	2.2212794	3.5040759	0.0009619	0.0098229	-0.915627
ELAVL4	-1.418774	9.1078008	-3.503715	0.000963	0.0098305	-0.916627
XLOC_00963	1.768755	4.3454816	3.5018966	0.0009683	0.0098595	-0.921665
SULT4A1	-1.149387	10.652366	-3.50141	0.0009697	0.0098646	-0.923012
CHN1	-1.166116	12.398401	-3.500677	0.0009719	0.0098798	-0.925043
PARM1	-1.262584	6.258821	-3.497831	0.0009802	0.0099372	-0.932921

NMNAT2	-1.496164	8.324399	-3.497109	0.0009824	0.0099463	-0.934922
FAT2	1.3745946	7.0041735	3.4891729	0.0010062	0.0101027	-0.95687
BATF	1.0792718	5.6031166	3.4874238	0.0010115	0.0101388	-0.961704
XLOC_00232	-1.165064	4.6253138	-3.485519	0.0010173	0.0101789	-0.966966
LOC151484	1.0511373	4.3333984	3.483078	0.0010248	0.0102241	-0.973707
XLOC_00261	1.0934241	3.8382041	3.4779762	0.0010407	0.0103379	-0.987787
SNORD17	1.6144723	9.297433	3.4768899	0.0010441	0.0103502	-0.990783
SNORD66	1.4226604	7.100567	3.4708608	0.0010632	0.0104616	-1.007403
CALB1	-1.753891	7.0577497	-3.469607	0.0010673	0.0104911	-1.010858
CAP2	-1.12464	7.692296	-3.4674	0.0010744	0.0105351	-1.016936
OR4A5	1.0068925	3.1038881	3.4671639	0.0010751	0.0105362	-1.017585
KCNS2	-1.032616	6.4040594	-3.46701	0.0010756	0.0105378	-1.01801
XLOC_0075C	1.1114686	4.1265617	3.4639861	0.0010854	0.010592	-1.026333
LOC643623	1.0270569	4.9072038	3.4557153	0.0011127	0.0107731	-1.049076
C6orf138	1.5340535	6.2073455	3.4534693	0.0011203	0.0108166	-1.055247
SNORD67	1.5656654	8.9535883	3.4500862	0.0011317	0.0108811	-1.064536
SNORA28	1.0080916	10.348198	3.448007	0.0011388	0.0109164	-1.070243
GPR4	1.2301234	6.5936924	3.4428207	0.0011566	0.0110511	-1.084468
C3orf30	1.1460348	2.3733474	3.4422991	0.0011584	0.0110579	-1.085898
SCG2	-1.615305	7.9092721	-3.440283	0.0011654	0.0110964	-1.091425
KIAA1239	-1.014258	5.566774	-3.437563	0.001175	0.0111475	-1.098876
SLCO4A1	1.3113161	9.8023171	3.4360107	0.0011804	0.0111723	-1.103128
XLOC_0042C	-1.105331	5.3069869	-3.434697	0.0011851	0.011203	-1.106725
LOC1001316	1.3879722	4.6789086	3.434448	0.001186	0.0112069	-1.107406
XLOC_0118C	1.2037415	6.5213714	3.4307171	0.0011993	0.0112742	-1.117616
XLOC_0041C	-1.143951	4.5099306	-3.429741	0.0012028	0.0112937	-1.120287
SYNGR3	-1.104373	8.6760835	-3.42938	0.0012041	0.011296	-1.121273
ABCC12	-1.071673	4.4217993	-3.424663	0.0012212	0.0114196	-1.134169
DIRAS3	-1.037327	5.1916032	-3.41648	0.0012514	0.0116275	-1.156515
XLOC_00495	2.3730802	8.8797895	3.4134717	0.0012627	0.0117019	-1.164722
FGF9	-1.293122	7.2967209	-3.408321	0.0012822	0.0118181	-1.178762
C3orf80	-1.258744	6.6636114	-3.40814	0.0012829	0.0118211	-1.179257
LPP-AS2	-1.058473	5.7404932	-3.406031	0.001291	0.0118542	-1.185001
YTHDC1	-1.113229	5.5220974	-3.405382	0.0012935	0.0118641	-1.18677
TAGLN3	-1.404402	9.7092429	-3.399265	0.0013173	0.0120306	-1.20342
POT1	1.1424776	8.0948815	3.3940325	0.0013379	0.0121605	-1.217646
PIK3R5	2.0027253	6.8480751	3.3915547	0.0013478	0.0122158	-1.224378
XLOC_01099	1.2270961	2.4184471	3.3913916	0.0013485	0.0122182	-1.224821
LOC1005076	-1.038709	4.6674436	-3.388377	0.0013606	0.0123017	-1.233008
OR52E8	2.1196531	6.9313554	3.3846855	0.0013756	0.0124011	-1.243026
XLOC_00625	2.0128317	5.4438614	3.3776983	0.0014045	0.0125726	-1.261971
XLOC_0039C	1.1194416	2.4666432	3.3752145	0.0014148	0.012648	-1.268699
ADRA1D	-1.067376	4.4985057	-3.374217	0.001419	0.0126713	-1.2714
XLOC_00165	1.7865888	5.3970019	3.3738906	0.0014204	0.0126765	-1.272285
CISH	1.8876644	8.8736791	3.3735291	0.0014219	0.0126831	-1.273263
NKX2-3	-1.038833	10.164423	-3.3687	0.0014424	0.0127912	-1.286333
RTN1	-1.505329	10.074254	-3.367627	0.001447	0.0128203	-1.289234
PRDM12	-1.244439	4.5966252	-3.359412	0.0014826	0.0130504	-1.311435
RBM24	-1.130209	5.8802108	-3.35778	0.0014898	0.013091	-1.315841
XLOC_01432	1.0821668	5.681254	3.3544019	0.0015048	0.0131971	-1.324959
XLOC_00317	1.1098041	2.7518918	3.3485053	0.0015312	0.0133534	-1.340859

LOC1005072	1.0168588	2.3231629	3.3476009	0.0015353	0.0133768	-1.343296
XLOC_00248	2.1179361	6.4981842	3.3394829	0.0015725	0.0136139	-1.365153
TAC3	-1.350811	8.5480921	-3.33862	0.0015766	0.0136375	-1.367474
XLOC_01113	1.9694651	6.320509	3.3324366	0.0016056	0.0138324	-1.384098
FAR2	-1.00394	5.9955075	-3.331562	0.0016097	0.0138575	-1.386448
XLOC_01080	-1.133205	4.7032664	-3.326468	0.001634	0.0140062	-1.400124
XLOC_00587	1.8296089	7.5718596	3.3245975	0.001643	0.0140573	-1.405144
INA	-1.079262	10.71293	-3.318475	0.0016729	0.0142254	-1.421561
XLOC_00133	2.1141541	8.2714074	3.3140717	0.0016947	0.0143574	-1.433355
XLOC_00008	1.8063723	6.9477708	3.3110632	0.0017097	0.0144632	-1.441408
KHDRBS2	-1.085915	6.872876	-3.309198	0.0017191	0.0145222	-1.446397
SCG5	-1.193683	11.611863	-3.304874	0.0017411	0.0146652	-1.457961
XLOC_00687	1.0152451	2.155521	3.3041711	0.0017447	0.01468	-1.459838
XLOC_00554	2.0649703	6.6603172	3.2977812	0.0017777	0.0148602	-1.476904
XLOC_00579	1.0008295	2.2191902	3.2975887	0.0017787	0.0148608	-1.477418
RSPO2	-1.425529	6.713399	-3.295731	0.0017884	0.0149225	-1.482375
ZBED6	1.1276762	8.6264128	3.2865024	0.0018373	0.0151898	-1.506976
SYT13	-1.152597	10.072805	-3.286482	0.0018375	0.0151898	-1.507031
PAK3	-1.057974	6.5897174	-3.28326	0.0018549	0.0152905	-1.515609
INSL3	-1.228688	9.2937442	-3.283137	0.0018555	0.0152918	-1.515934
XLOC_01085	2.22481	8.0392661	3.2815049	0.0018644	0.0153216	-1.520279
ASB16	-1.137267	8.5224054	-3.278912	0.0018786	0.0154027	-1.527176
KRT222	-1.178924	7.6529424	-3.278135	0.0018828	0.0154298	-1.529242
ATXN3L	1.0026853	4.5276798	3.2774009	0.0018869	0.0154489	-1.531194
LOC1005075	-1.460886	8.9705212	-3.277159	0.0018882	0.015449	-1.531836
XLOC_00791	1.0300509	4.0686329	3.2742826	0.0019041	0.0155513	-1.539481
XLOC_00702	2.216935	9.194474	3.273526	0.0019084	0.0155752	-1.541491
XLOC_00666	-1.154212	8.6984807	-3.270016	0.001928	0.0156996	-1.550812
TAC1	-1.775009	6.252355	-3.269845	0.001929	0.0157022	-1.551266
XAGE-4	1.1767776	4.620001	3.2657958	0.0019519	0.0158206	-1.562012
DEFB116	1.0673874	2.8239497	3.2651354	0.0019556	0.0158286	-1.563763
CCDC66	1.7100045	9.1178723	3.2635037	0.001965	0.0158796	-1.56809
ELOVL4	-1.041646	7.7551013	-3.259584	0.0019875	0.0160096	-1.578478
FMR1NB	1.3469524	5.414767	3.2576876	0.0019985	0.0160812	-1.583502
XLOC_00547	1.258782	9.9977014	3.2551375	0.0020134	0.0161695	-1.590253
C1QL3	-1.160294	6.1875918	-3.253317	0.0020241	0.0162269	-1.595071
XLOC_00906	-1.049528	2.4178307	-3.247915	0.0020562	0.0164019	-1.609357
XLOC_00211	-1.007524	8.6712309	-3.24753	0.0020585	0.0164161	-1.610374
ZFP36	1.0238519	9.5468489	3.2405665	0.0021006	0.0166523	-1.628767
XLOC_01113	2.0554504	7.592025	3.2385851	0.0021127	0.0166988	-1.633995
SNORA27	1.0735691	9.0652118	3.2369201	0.0021229	0.0167431	-1.638387
XLOC_01111	1.1737878	2.4528708	3.2342904	0.0021392	0.0168297	-1.645321
NRIP3	-1.061049	6.2337658	-3.233159	0.0021462	0.0168806	-1.648302
XLOC_00418	2.1549258	8.5912254	3.2285313	0.0021752	0.0170459	-1.660493
GPR182	2.2096527	9.2665372	3.227878	0.0021793	0.017074	-1.662213
OSR1	1.0567805	2.5847552	3.2258373	0.0021922	0.0171334	-1.667584
LY6D	-1.02214	6.4688757	-3.223204	0.002209	0.0172352	-1.674511
LINC00230A	-1.561016	5.3840762	-3.219834	0.0022307	0.0173527	-1.683372
XLOC_01286	1.1370144	5.4877562	3.2178416	0.0022436	0.0174327	-1.688608
XLOC_01020	1.7199292	5.118175	3.2093968	0.002299	0.0176972	-1.710776
RASL11B	-1.085456	8.0894985	-3.209119	0.0023009	0.017705	-1.711505

SNORA45	1.0555543	8.0099871	3.2057611	0.0023233	0.0178019	-1.720308
XLOC_0023C	1.021968	2.3164996	3.1952124	0.0023951	0.0182078	-1.747925
XLOC_00727	1.0260122	4.0901739	3.1947283	0.0023984	0.0182252	-1.749191
PCDHB7	-1.013384	3.5755861	-3.191124	0.0024234	0.0183416	-1.758614
LOC1005073	1.4773784	5.0118264	3.190753	0.002426	0.0183482	-1.759582
SFN	1.0269787	6.1886874	3.1891834	0.002437	0.0184183	-1.763682
LOC338797	-1.148981	9.9997983	-3.189003	0.0024383	0.0184203	-1.764153
XLOC_00131	1.7770475	5.7025291	3.1815793	0.0024909	0.0186979	-1.783528
XLOC_00919	1.9406855	10.509683	3.1811978	0.0024937	0.0187112	-1.784523
XLOC_01229	2.1335945	9.280275	3.1798256	0.0025035	0.0187705	-1.788101
OR52I2	1.815036	4.9731847	3.1784228	0.0025136	0.0188044	-1.791757
EPHA5	-1.180508	5.4973453	-3.178324	0.0025143	0.0188044	-1.792016
XLOC_12_01	1.3754579	4.6241578	3.1756119	0.002534	0.0188939	-1.799081
SFRP1	1.0877191	7.8551371	3.17375	0.0025476	0.018968	-1.80393
XLOC_01394	-1.041519	7.9845573	-3.163769	0.0026216	0.0193719	-1.82989
XLOC_00528	1.1510708	4.0203536	3.1626648	0.0026299	0.0194187	-1.832758
XLOC_00755	1.2983031	4.5916431	3.1555981	0.0026837	0.0196799	-1.851102
GPR179	2.1134526	9.7729666	3.1511487	0.0027181	0.0198821	-1.862637
NELL2	-1.057032	10.292334	-3.150049	0.0027267	0.0199265	-1.865486
CDH8	-1.35347	5.5081459	-3.149723	0.0027292	0.0199337	-1.866332
Q5A5F0	1.4131848	7.9882968	3.1488573	0.002736	0.0199491	-1.868574
XLOC_12_01	1.8779397	6.805304	3.1463291	0.0027558	0.0200437	-1.87512
PCSK1	-2.075271	8.597486	-3.145238	0.0027644	0.0200918	-1.877946
CASQ1	-1.172228	8.2505324	-3.143081	0.0027815	0.0201712	-1.883527
OSTBETA	2.0100177	6.1205763	3.1371837	0.0028287	0.0204076	-1.898773
XLOC_00769	-1.211136	4.9443373	-3.132719	0.002865	0.020576	-1.910302
GABRB2	-1.373789	9.5793599	-3.132623	0.0028658	0.020576	-1.910552
XLOC_00119	1.081366	5.90808	3.1317034	0.0028733	0.0206132	-1.912924
XLOC_00189	1.8819342	6.9314332	3.1286336	0.0028986	0.0207294	-1.920844
DYNC1I1	-1.268069	7.7417669	-3.118857	0.0029804	0.0211355	-1.946032
FLJ42842	1.1688729	4.1021951	3.1186959	0.0029818	0.0211358	-1.946447
ZCCHC13	1.6166815	5.1000849	3.1173326	0.0029933	0.0211992	-1.949955
XLOC_12_0C	-1.178832	2.8585246	-3.110952	0.0030481	0.0214452	-1.96636
GLS2	-1.068792	7.2918682	-3.103513	0.0031132	0.0217298	-1.985459
XLOC_12_0C	1.0719462	5.5988444	3.1003915	0.0031409	0.0218362	-1.993462
NLGN4Y	-1.020515	5.5190155	-3.099596	0.003148	0.02186	-1.995502
REEP1	-1.06427	8.697676	-3.095562	0.0031842	0.0220255	-2.005835
XLOC_00976	2.0657628	8.5570208	3.0939375	0.0031989	0.0221033	-2.009995
KRT79	2.1667265	8.2025974	3.0903187	0.0032318	0.022281	-2.019255
XLOC_00369	-1.072335	10.166209	-3.090274	0.0032322	0.022281	-2.01937
LOC1001295	-1.085525	6.1633589	-3.088915	0.0032447	0.0223429	-2.022846
RNASE3	1.015404	4.1206796	3.0684576	0.0034377	0.0232709	-2.075039
XLOC_01229	1.8128466	6.7030835	3.0573905	0.0035466	0.0237885	-2.103177
CEP72	-1.010891	6.3534346	-3.055712	0.0035634	0.0238512	-2.107438
PPBP	1.054482	4.4283975	3.0547732	0.0035728	0.0238735	-2.109822
RPA4	1.8693698	10.577746	3.0536233	0.0035844	0.0239119	-2.112739
NEUROD6	-1.239006	4.9378759	-3.049831	0.0036228	0.0240851	-2.122358
XLOC_12_0C	2.0403617	10.039548	3.0498008	0.0036231	0.0240851	-2.122434
XLOC_01417	-1.105382	10.452885	-3.047367	0.003648	0.0241934	-2.128603
ZCCHC12	-1.098322	5.482084	-3.047057	0.0036511	0.0241934	-2.129387
XLOC_01336	1.9819766	7.5542581	3.0456737	0.0036653	0.0242554	-2.132892

C1orf182	-1.104054	8.9728082	-3.036074	0.0037654	0.02475	-2.157179
XLOC_0070C	1.066854	2.7088684	3.0349959	0.0037768	0.0248049	-2.159903
C13orf35	1.0250716	2.3263056	3.0324202	0.0038042	0.0249273	-2.16641
LOC284578	-1.158531	6.0571807	-3.031613	0.0038128	0.0249633	-2.168447
XLOC_01283	1.5868272	7.8462213	3.0275457	0.0038565	0.0252081	-2.178712
NAP1L2	-1.080391	9.9602312	-3.022871	0.0039072	0.0254621	-2.190498
FPR1	1.0286799	8.2038774	3.0204165	0.0039341	0.0255648	-2.19668
C4orf6	1.2651667	6.7403697	3.0159075	0.003984	0.0257844	-2.20803
PNMA2	-1.033773	9.7414112	-3.012014	0.0040276	0.0259876	-2.217821
XLOC_12_0C	-1.024821	7.7003175	-3.006821	0.0040864	0.0262627	-2.230867
MCF2	-1.155326	6.1704981	-3.003524	0.0041241	0.0264296	-2.239141
XLOC_00855	1.6297785	12.414878	3.0016328	0.0041459	0.0264898	-2.243883
XLOC_00438	2.0439895	9.5370117	2.9940333	0.0042345	0.0269008	-2.262923
CPNE4	-1.583848	7.8783558	-2.983785	0.0043569	0.0274175	-2.288546
XLOC_01027	1.3985473	5.9316639	2.9826467	0.0043707	0.0274509	-2.291389
SNAP25	-1.208736	15.75575	-2.975762	0.004455	0.0277569	-2.308564
XLOC_01179	1.0127858	3.0142242	2.9633397	0.0046109	0.0284789	-2.339482
SH3GL2	-1.354709	9.5169987	-2.963208	0.0046126	0.0284789	-2.33981
ZNF184	-1.037503	6.8643763	-2.960587	0.0046461	0.0285906	-2.346321
CTXN3	-1.645111	6.5286044	-2.959875	0.0046553	0.0286269	-2.34809
BTBD11	-1.082488	6.4054671	-2.955204	0.0047157	0.028888	-2.359683
XLOC_00749	1.2459452	4.9568503	2.9542819	0.0047278	0.0289419	-2.36197
GSTM4	-1.059959	7.1412869	-2.954252	0.0047282	0.0289419	-2.362044
WBSCR17	-1.023166	7.2543495	-2.95383	0.0047337	0.0289591	-2.36309
SLITRK5	-1.090338	6.4339115	-2.949454	0.0047912	0.0291594	-2.373937
KCNC2	-1.119832	8.1348426	-2.949403	0.0047919	0.0291594	-2.374063
SYT4	-1.357078	10.28794	-2.946307	0.004833	0.029326	-2.381729
PTPN5	-1.207043	6.0815506	-2.944172	0.0048615	0.0294323	-2.387013
PRAMEF12	1.2187989	7.2071913	2.939575	0.0049235	0.0296953	-2.398381
XLOC_01082	1.6395707	6.3047248	2.9359302	0.0049731	0.0299385	-2.407385
B4GALT6	-1.201638	6.0027897	-2.924986	0.005125	0.0305504	-2.434375
ACOT7	-1.068395	9.3736627	-2.923372	0.0051478	0.0306448	-2.438347
XLOC_01368	1.283941	5.1922267	2.921373	0.0051761	0.0307677	-2.443268
XLOC_0094C	1.2594404	8.009887	2.9191645	0.0052075	0.0308794	-2.448701
XLOC_00452	1.9975268	8.7437966	2.9157542	0.0052565	0.0310872	-2.457085
PPP6R1	1.7480757	11.421678	2.9147311	0.0052712	0.0311436	-2.459599
XLOC_00709	-1.054366	9.3094501	-2.914663	0.0052722	0.0311436	-2.459765
ERAP2	1.0360144	6.4745579	2.9126613	0.0053012	0.0312395	-2.464682
LOC400622	-1.046151	6.4439463	-2.904511	0.0054208	0.0316996	-2.484674
XLOC_01262	1.1142434	10.100208	2.903521	0.0054355	0.0317451	-2.487101
XLOC_00028	-1.274564	3.9370214	-2.902227	0.0054548	0.0318345	-2.490272
IPCEF1	-1.072043	5.8191797	-2.901011	0.005473	0.0319054	-2.49325
PABPC1L2A	-1.289145	5.556102	-2.898515	0.0055104	0.0320715	-2.499359
C4orf51	1.23914	4.0562786	2.8975374	0.0055251	0.0321398	-2.501751
KDM5D	-1.548644	5.4692353	-2.893253	0.0055902	0.0324099	-2.512228
XLOC_00566	1.4359509	5.7018202	2.8863977	0.0056957	0.0328318	-2.528967
PLCXD3	-1.165771	8.0708853	-2.882872	0.0057507	0.0330441	-2.537566
SNORD97	1.0119162	7.166234	2.878554	0.0058187	0.0333096	-2.548086
XLOC_00233	-1.004424	11.247716	-2.872339	0.0059178	0.0337446	-2.563208
XLOC_00166	-1.066873	9.7438196	-2.851132	0.0062683	0.0350208	-2.614633
XLOC_12_01	-1.091287	8.095087	-2.846757	0.0063429	0.0353055	-2.625211

MAGEB6	2.0198706	5.9957807	2.8435776	0.0063977	0.0355213	-2.632888
XLOC_0004S	1.1123364	7.0160242	2.8428721	0.0064099	0.0355581	-2.634591
KCNJ6	-1.196734	7.6687615	-2.824857	0.0067291	0.0368886	-2.677972
PCK1	1.2013833	4.5011782	2.806488	0.0070696	0.0382262	-2.722
GPR22	-1.087267	6.4783067	-2.804269	0.0071118	0.0384157	-2.727304
VPS18	1.3454026	13.545675	2.7948117	0.0072943	0.0390919	-2.749878
NXPH2	-1.046024	3.9306313	-2.789022	0.0074081	0.0395237	-2.763671
XLOC_12_0C	-1.178587	7.7829093	-2.782105	0.0075463	0.0400347	-2.780121
NMU	-1.302236	4.8934556	-2.781644	0.0075556	0.0400469	-2.781216
XLOC_0114E	-1.286427	8.0329175	-2.755366	0.008103	0.0420997	-2.843434
WNT2B	1.0598093	6.8711834	2.7545727	0.0081201	0.0421511	-2.845305
RGS1	1.2659356	8.7228348	2.743664	0.0083584	0.0430743	-2.871001
CNNM1	-1.03481	5.400414	-2.733105	0.0085951	0.0439203	-2.8958
XLOC_0013Z	-1.278797	7.2438797	-2.730895	0.0086454	0.0440986	-2.900983
PPM1E	-1.09391	5.8753267	-2.724642	0.0087893	0.0445594	-2.915628
SMEK3P	1.01923	2.5996809	2.7237746	0.0088094	0.0446043	-2.917657
XLOC_0059E	1.2976123	5.2325797	2.7236084	0.0088133	0.0446043	-2.918046
C13orf30	1.5123408	5.446684	2.7188031	0.0089256	0.0449574	-2.92928
XLOC_0110S	1.2719351	6.7114416	2.7139334	0.0090408	0.0453737	-2.94065
XLOC_0039S	1.071668	2.0409684	2.7088532	0.0091624	0.0457838	-2.952496
NUDT11	-1.010248	5.3501953	-2.701504	0.009341	0.0464371	-2.969603
MUCL1	-1.121733	4.0776331	-2.69587	0.00948	0.046883	-2.982694
AMPH	-1.275861	7.9164019	-2.680273	0.009875	0.0483278	-3.018827
SNORD38A	1.3125807	8.9569536	2.6669387	0.0102246	0.0495996	-3.049597
