# Supplementary figures and images for: Identification and Experimental Validation of Marker Genes between Diabetes and Alzheimer's Disease
Source: Oxid Med Cell Longev. 2022 Aug 12;2022:8122532. doi: 10.1155/2022/8122532 (PMC9391608; doi:10.1155/2022/8122532)

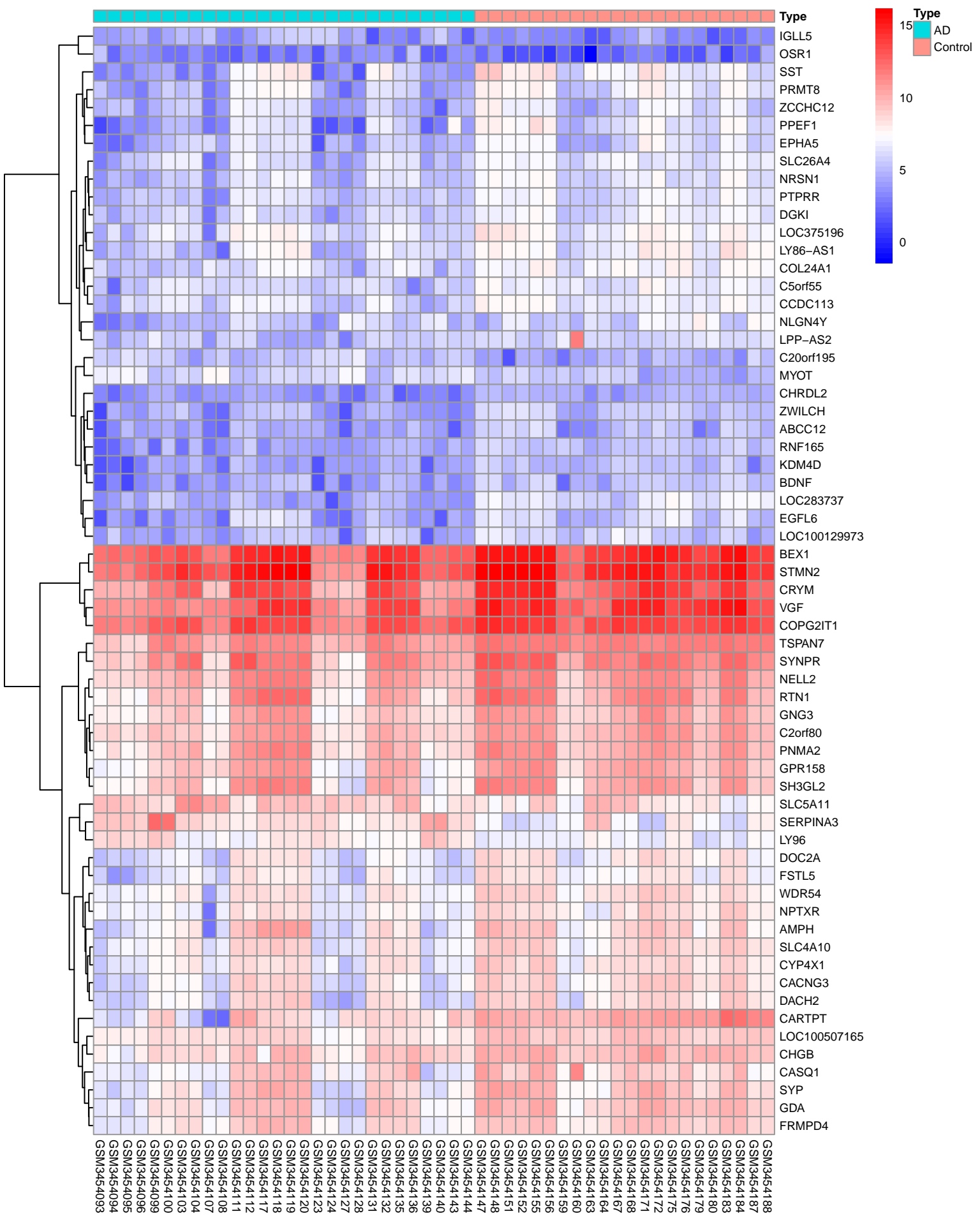

Supplement: Supplementary 4 — Supplementary Figure 1: the heatmap for the expression level of 62 overlapping genes in GSE122063. [file 8122532.f4.pdf]

CON AD

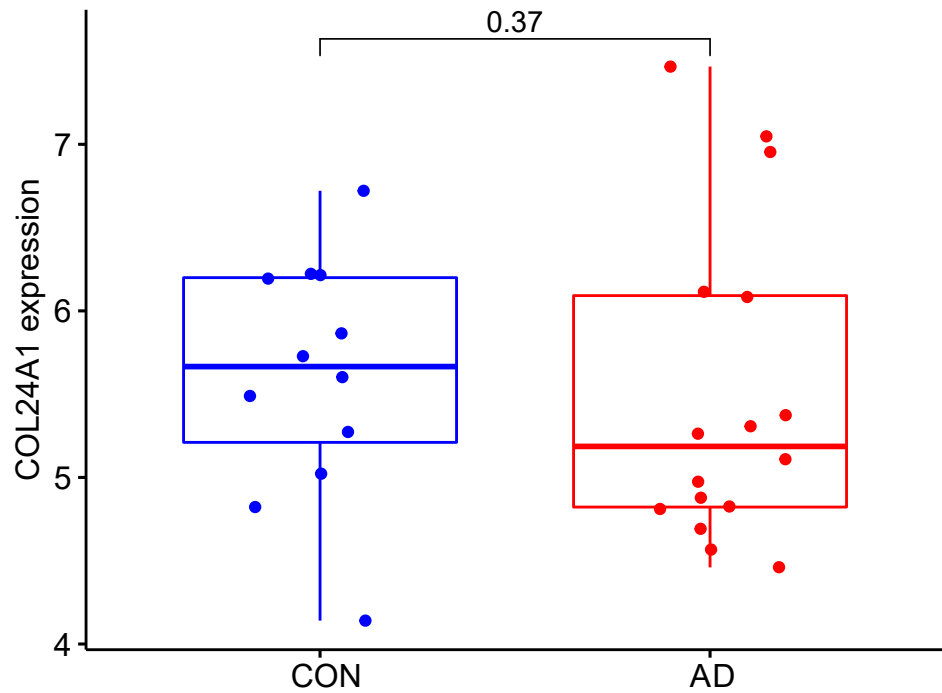

Supplement: Supplementary 5 — Supplementary Figure 2: the expression value of COL24A1 validated in the GSE5281 (no significance). [file 8122532.f5.pdf]

CON AD

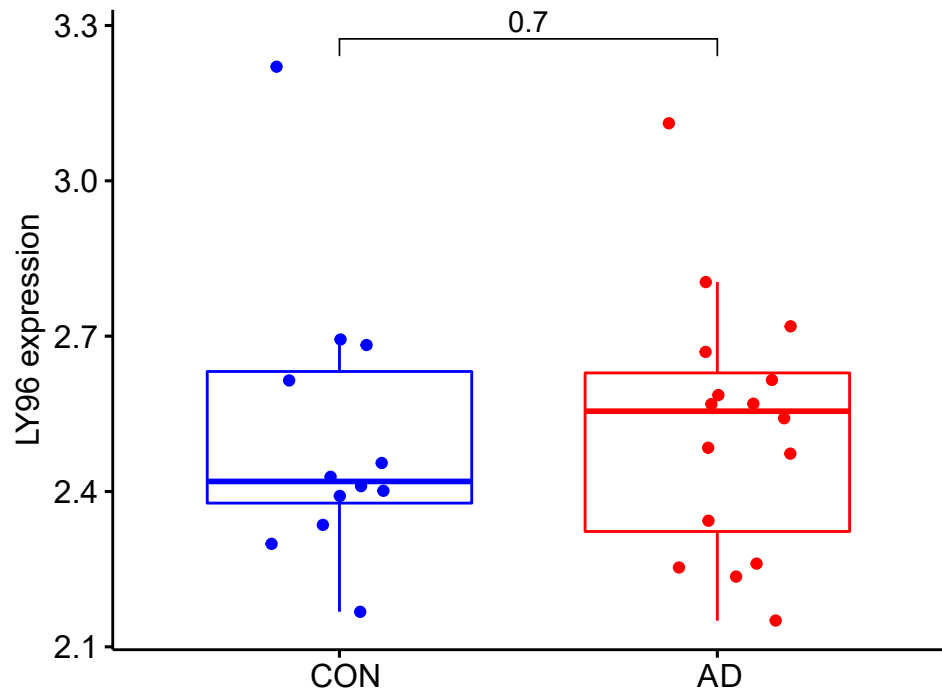

Supplement: Supplementary 6 — Supplementary Figure 3: the expression value of LY96 validated in the GSE5281 (no significance). [file 8122532.f6.pdf]
